# A PLA_2_ deletion mutant using CRISPR/Cas9 coupled to RNASeq reveals insect immune genes associated with eicosanoid signaling

**DOI:** 10.1371/journal.pone.0304958

**Published:** 2024-07-17

**Authors:** Mohammad Vatanparast, Mojtaba Esmaeily, David Stanley, Yonggyun Kim

**Affiliations:** 1 Department of Plant Medicals, Andong National University, Andong, Korea; 2 Federal Research Centre for Cultivated Plants, Epigenetics and RNAi Lab, Institute for Biosafety in Plant Biotechnology, Julius Kühn Institute (JKI), Quedlinburg, Germany; 3 USDA/Agricultural Research Service, Biological Control of Insects Research Laboratory, Columbia, MO, United States of America; University of Texas Medical Branch at Galveston, UNITED STATES

## Abstract

Eicosanoids mediate insect immune responses and synthesized by the catalytic activity of phospholipase A_2_ (PLA_2_). A uniquely encoded secretory PLA_2_ (sPLA_2_) is associated with immune responses of a lepidopteran insect, *Spodoptera exigua*. Its deletion mutant was generated using a CRISPR/Cas9 genome editing technology. Both wild and mutant lines were then immune-challenged, and the resulting transcripts were compared with their naïve transcripts by RNASeq using the Illumina-HiSeq platform. In total, 12,878 unigenes were further analyzed by differentially expressed gene tools. Over 69% of the expressed genes in *S*. *exigua* larvae are modulated in their expression levels by eicosanoids, recorded from CRISPR/Cas9 mutagenesis against an eicosanoid-synthetic gene, *Se-sPLA*_*2*_. Further, about 36% of the immune-associated genes are controlled by the eicosanoids in *S*. *exigua*. Indeed, the deletion mutant suffered significant immunosuppression in both cellular and humoral responses in response to bacterial challenge as well as severely reduced developmental and reproductive potentials.

## 1. Introduction

Prostaglandins and other eicosanoids are oxygenated derivatives of C20 polyunsaturated fatty acids (PUFAs). They mediate wide range of physiological processes in vertebrates and invertebrates [1–3]. They include three chemical groups: prostaglandins (PGs), leukotrienes (LTs), and epoxyeicosatrienoic acids (EETs). They are synthesized from C20 PUFAs, including arachidonic acid (AA, 20:4n-6), γ-linolenic acid (20:3n-6), and eicosapentaenoic acid (20:5n-3) [4]. AA and other PUFAs are preferentially associated with phospholipids (PLs) that make up biological membranes whereas they exist in much lower proportions in neutral, energy-storage lipids such as triacylglycerols [5]. Various phospholipase A_2_s (PLA_2_s) are responsible for hydrolyzing PUFAs from PLs in mammals and insects [6, 7]. In terrestrial insects, trace amounts of AA are detected in PLs presumably to avoid oxidative stress induced by massive oxygen entry through the well-developed tracheal system [8]. Instead, a large amount of linoleic acid (18:2n-6) is released by PLA_2_ and converted into AA by elongase and desaturases [9]. In mammals, PGs and other prostanoids are synthesized from AA by cyclooxygenase (COX). Insects do not have a COX ortholog [10], but they use specific peroxidases, called peroxynectins, to produce PGH_2_ [11, 12]. PGH_2_ is then isomerized into PGE_2_, PGD_2_, and PGI_2_ by cell-specific synthases [13–15]. LTs also mediate some insect immune responses, but their biosynthetic pathway requires further exploration because they do not encode any ortholog to the vertebrate lipoxygenase. However, a recent discovery of a specific double oxidase, presumably associated with LT biosynthesis in a mosquito, *Anopheles gambiae*, suggests an alternative LT biosynthetic pathway [16]. EETs occur in insects, and their biosynthetic enzymes catalyse epoxidation at a specific position of AA [17]. Thus, the catalytic activity of PLA_2_ is the first committed step for eicosanoid biosynthesis in insects as well as most metazoans.

PLA_2_ hydrolyses fatty acids from the *sn-2* position of PLs [18, 19]. Diverse gene structures of PLA_2_s originate from prokaryotes to eukaryotes and form a large gene superfamily consisting of at least 16 groups (I-XVI) that are categorized by their amino acid sequences, molecular weights, disulfide bonds, and Ca^2+^ requirements [20]. These PLA_2_s include the three classical types: Ca^2+^-dependent intracellular PLA_2_ (cPLA_2_), Ca^2+^-independent intracellular PLA_2_ (iPLA_2_), and secretory PLA_2_ (sPLA_2_) [21], all of which have been identified in insects [22]. sPLA_2_s are characterized by their relatively small size and multiple disulfide bonds. Several venomous sPLA_2_s have been identified in hymenopterans, including honey bee [23], and they occur in various insect orders and are classified into Group III, non-venomous sPLA_2_s. In addition to a dipteran species, *Drosophila melanogaster* [24], five sPLA_2_ have been reported in the coleopteran, *Tribolium castaneum*, in which four of these enzymes are known to act in cellular immune response [25]. Two different molecular forms of sPLA_2_s have been reported in a hemipteran insect, *Rhodnius prolixus*, and they are classified into groups II and XII [26] These two PLA_2_s act in immune functions associated with eicosanoid biosynthesis [27]. A single sPLA_2_, classified into Group III, is encoded in a lepidopteran, *Spodoptera exigua*, and its high enzyme activity in larval hemolymph and midgut is associated with immunity and digestion [28, 29].

Insect immunity is innate and it features the programmed responses from pathogen recognition to immune reactions [30]. Once pathogens are recognized by pattern recognition receptors, the nonself signal propagates to nearby immune effector tissues, the hemocytes and fat body [31]. PGs and other eicosanoids act as autocrine and paracrine signals and activate cellular and humoral immune responses [22]. PGs activate sessile hemocytes to increase total circulating hemocytes [32]. The eicosanoid signalling guides circulating hemocytes toward infection foci [33]. As the migrating hemocytes contact pathogens, they initiate cellular immune responses such as phagocytosis, nodulation, or encapsulation, depending on the size and density of the pathogens [34]. For these cellular immune responses, hemocytes exhibit spreading behavior through the F-actin growth in the cytoskeleton to form filopodia or lamellopodia [35]. This actin remodelling is mediated by Fascin, an actin-bundling protein, which is activated by PGE_2_ [36]. Subsequent melanin formation around pathogens is catalysed by phenoloxidase (PO). PGs signal release of an inactive pro-PO from a specific hemocyte type, oenocytoids, into hemolymph [37]. Aside from releasing pro-PO into hemolymph, other hemocytes directly attack infecting microbes and engulf them by phagocytosis. The internalized microbes are killed by eicosanoid-triggered reactive oxygen species [38]. Eicosanoids also mediate antimicrobial peptide synthesis in insects [39, 40]. Overall, eicosanoids are essential signals that mediate insect immune responses. Other invertebrates may have also evolved eicosanoid-mediated immune signalling [41]. Some bacterial species protect themselves from insect host immune reactions. Members of two entomopathogenic bacterial genera, *Xenorhabdus* and *Photorhabdus*, produce secondary metabolites that inhibit insect PLA_2_-derived signalling to suppress host insect immunity [42, 43].

Various immune factors are involved in defending from pathogen infection in insects. For example, the diamondback moth, *Plutella xylostella*, expresses 129 immune-associated genes to defend from destruxin A, a mycotoxin of the entomopathogenic fungus, *Metarhizium anisopliae* [44]. *S*. *exigua* modulates the expressions of 1,785 midgut genes in defending against viral infections [45]. These findings suggest that eicosanoids may influence the expression of a large number of target genes to modulate insect immunity. Determining the target genes would be very useful in understanding eicosanoid-mediated immunological processes. To identify the immunity-related target genes modulated by eicosanoids, we developed a strategy involving knocking-out a *S*. *exigua* PLA_2_ gene using a clustered regularly interspaced short palindromic repeats (CRISPR)/CRISPR-associated protein 9 (Cas9) protocol, then monitoring the resulting alterations in transcriptomes using differentially expressed gene (DEG) analysis through RNA-sequencing (RNASeq).

## 2. Materials and methods

### 2.1. Insect rearing and egg collection

*S*. *exigua* larvae developed through five instars (L1-L5) on an artificial diet [46], under our standard laboratory conditions (25 ± 2°C, 16:8 h (L:D) photoperiod, 60 ± 5% relative humidity (RH)). Pupae were obtained from the colony, and freshly emerged adults were maintained in a cage (30 × 25 × 25 cm) and provided with 10% sugar solution. Female *S*. *exigua* moths were allowed to lay eggs on a plastic petri dish. All results reported in this paper emerged from *S*. *exigua*, which obviates further mention of the species name.

### 2.2. Chemicals

A PLA_2_ assay kit was purchased from Cayman Chemical (Ann Arbor, MI, USA). Bovine serum albumin (BSA) and arachidonic acid (AA: 5,8,11,14-eicosatetraenoic acid) were purchased from Sigma Aldrich Korea (Seoul, Korea). Phosphate-buffered saline (PBS) was prepared with 100 mM phosphoric acid, and its pH was calibrated to 7.4 using 1 N NaOH.

### 2.3. Immune challenge

For immune challenge, an entomopathogenic bacterium, *Xenorhabdus nematophila* ANU101 [47], was cultured overnight in Luria-Bertani broth. The cultured bacteria were then killed by heat treatment at 95°C for 5 min for the immune challenge. Individual L5 larvae were injected with 5 × 10^4^ cells of the heat-killed bacteria in a 1 μL volume using a microsyringe (Hamilton, Reno, NV, USA).

### 2.4. RNA extraction, PCR, and RT-qPCR

RNA samples were extracted from the whole L5 larvae using a Trizol reagent (Invitrogen, Carlsbad, CA, USA). After extraction, the RNA was resuspended in nuclease-free water and quantified using a spectrophotometer (NanoDrop, Thermo Fisher Scientific, Wilmington, DE, USA). cDNA was then synthesized from RNA (1 μg) using RT PreMix (Intron Biotechnology, Seoul, Korea) containing an oligo dT primer according to the manufacturer’s instruction. All quantitative PCRs (qPCRs) in this study were determined using a real-time PCR instrument (Step One Plus Real-Time PCR System, Applied Biosystems, Singapore) and a Power SYBR Green PCR Master Mix (Life Technologies, Carlsbad, CA, USA) according to the guidelines outlined by Bustin et al [48]. The reaction mixture contained 10 μL of Power SYBR Green PCR Master Mix, 5 μL of cDNA template (50 ng), and 1 μL each of the forward and reverse primers (S1 Table). qPCR temperature cycling began with 95°C heat treatment for 10 min, followed by 40 cycles of denaturation at 94°C for 30 s, annealing at different temperatures (S1 Table) for 30 s, and extension at 72°C for 30 s. The expression level of a ribosomal protein, *RL32*, as a reference gene was used to normalize target gene expression levels under different treatments. PCR products were assessed through melting curve analysis. Quantitative analysis was performed using the comparative CT (2^−ΔΔCT^) method [49].

### 2.5. Western blotting against Se-sPLA_2_

Extracted proteins (100 μg per sample) were separated on 10% SDS-PAGE. The separated proteins in the gel were transferred onto a 0.2 μm pore nitrocellulose membrane (BioRad) for 45 min at 100 V in a chilled transfer buffer (25 mM Tris base, 190 mM glycine, 20% methanol, pH 8.5). The membrane was briefly rinsed with Tris-buffered saline containing Tween 20 (TBST) (20 mM Tris, 150 mM NaCl, and 0.1% Tween 20, pH 7.5) and then blocked with 3% BSA in TBST at room temperature for 1 h. Next, membranes were incubated at 4°C for 2 h with a polyclonal antibody raised against a recombinant Se-sPLA_2_ protein [50], as the primary antibody diluted 5,000 times with TBST containing 3% BSA. The membranes were then washed three times with TBST (5 min per wash) and incubated with anti-rabbit IgG-alkaline phosphatase secondary antibody (Sigma-Aldrich Korea) at a dilution of 1:30,000 in TBST containing 3% BSA for 1 h at room temperature. Blots were rinsed three times with TBST. To detect alkaline phosphatase activity, nitrocellulose membrane blots were incubated with a substrate (5-bromo-4-chloro-3-indolyl phosphate/nitro blue tetrazolium, Sigma-Aldrich Korea).

### 2.6. PLA_2_ enzyme assay

PLA_2_ enzyme activities were measured from the whole bodies of selected developmental stages. Each protein extract was obtained from > 1,000 eggs, > 10 individuals from L1-L3, individual L4 and L5 larvae, or individual pupae and adults using PBS. The enzyme activities of the extracts were measured using a sPLA_2_ assay kit (Cayman Chemical) containing a diheptanoyl thio-phosphatidyl choline substrate. L5 larvae were also used to collect hemolymph, which was then separated into hemocytes and plasma by centrifugation at 800 × *g* for 3 min at 4°C. The resulting plasma was used to measure sPLA_2_ enzyme activity. All treatments included three biologically independent replicates. Lastly, protein concentrations were determined by Bradford [51] assay using BSA as standard.

### 2.7. Guide RNA design and ribonucleoprotein complex preparation

The CRISPR RNA (crRNA) used in this study was designed using tools available at http://chopchop.cbu.uib.no/ with the aim of determining the optimal target sites. For single strand guide RNA (sgRNA) *in vitro* transcription, a specific oligonucleotide encoding a T7 polymerase-binding site (grey-shaded), target sequence GGN_20_ (bolded), and scaffold template-specific sequence (underlined) was designed as the forward primer sgRNA-F1 (57 bp) (5′-CCTCTA ATA CGA CTC ACT ATA G**GT GTG CTG GCA ATG TGG G**GT TTA AGA GCT ATG C-3′), and sgRNA-F2 (58 bp) (5′-CCTCTA ATA CGA CTC ACT ATA GG**T GTC CCG ATT TGA TCC CCG G**GT TTA AGA GCT ATG C-3′) was designed according to the Guide-it sgRNA In Vitro Transcription kit (Takara Korea Biomedical, Seoul, Korea). Briefly, PCR was performed with a customer-based forward primer and a company-provided reverse primer. The PCR product (~130 bp) was used for *in vitro* transcription using T7 RNA polymerase to produce sgRNAs. The sgRNA produced in this way was purified using a spin column provided in the kit; the final amount was quantified using the NanoDrop spectrophotometer. Guide-it Recombinant Cas9 Nuclease protein with a nuclear localization site signal (Takara Korea Biomedical) was used to make ribonucleoprotein complex with the purified sgRNA.

### 2.8. Preparation of *S*. *exigua* eggs for microinjection

To obtain the freshly laid eggs for mutagenesis, a plastic petri dish was replaced every 0.5 h. The egg masses on the dishes were cleaned with a brush to remove female hair-pencils. Only the dish area having an egg mass was cut out using a hot scalpel, while the resulting plastic piece was fixed on a glass slide with one small droplet of instant glue. The glass slides were placed in a desiccator containing silica gel, and the eggs were dehydrated with the application of approximately -100 kPa vacuum for 10 min.

### 2.9. Microinjection of gRNA and Cas9 into eggs

Injection needles were prepared by pulling borosilicate glass capillary tubes (TW100-4, World Precision Instrument, Sarasota, FL, USA) using a sutter nitrogen picopump injector (PV830, World Precision Instrument) under a stereomicroscope (SZX9-ILLK200, Olympus, Japan)-based horizontal glass capillary puller (PN-30, Narishige, Tokyo, Japan). After back-filling approximately 3 μL of each injection mixture of Cas9 (300 ng/μL) plus sgRNA (100 ng/μL) in RNase-free water, about 5 nL of the mixture was injected into an egg through a micropyle using a Narishige micromanipulator model MM-33. All microinjections were completed within 1.5 h after oviposition and within 1 h after egg collection. Slides containing injected eggs were put into a 100 mm-diameter petri dish layered with a moist filter paper and covered with a lid. These petri dishes were sealed with parafilm and placed into a plastic box designated “secondary containment”. Eggs were incubated at room temperature for approximately 4 h before transfer to a designated incubator set to 25°C with 70% RH. Hatched neonates were transferred to a fresh dish and fed with artificial diet.

### 2.10. Genotyping and DNA analysis

Genomic DNA was extracted from the final instar ecdysed cuticle (= exuviae) using 10% Chelex 100 (Bio-Rad, Richmond, CA, USA) in 10 mM Tris-HCl buffer (pH 8.0) containing 0.1 mM EDTA according to Nguyen et al [52]. Briefly, in a 1.5 mL tube, 500 μL of 10% Chelex was added on each larval exuviae and each tube was vortexed vigorously for 20 s. After incubation at 56°C for 10 min, the samples were incubated again at 100°C for 15 min, then centrifuged at 14,000 × *g* for 4 min the resulting supernatant was transferred to a new 1.5 mL tube. Forward and reverse diagnostic primers were designed to cover both sgRNA target sites of the sPLA_2_ gene to obtain a 230 bp PCR product as the wild type (S1 Table). PCR amplification was conducted using Taq DNA polymerase (GeneALL, Seoul, Korea) with an initial heat treatment at 94°C for 5 min followed by 35 cycles of DNA denaturation at 94°C for 30 s, primer annealing at 52°C for 30 s (S1 Table), and extension at 72°C for 30 s. The PCR reaction was completed with a final chain extension step at 72°C for 10 min.

### 2.11. Purifying homozygotes for the CRISPR mutant

Based on the CRISPR mutant deleting the target region (= 85 bp) between two sgRNA differences in the CRISPR target region, a 230 bp region covering the target region was amplified with diagnostic primers (S1 Table). This diagnostic PCR produces a single band at 230 bp in wild type individuals while it also produces 145 bp PCR product in the CRISPR mutant. For the same reason, heterozygote mutants should show two bands at 145 and 230 bp. Based on this PCR molecular marker, the parent generation (G0) subjected to the CRISPR/Cas9 treatment was scored, and mutants were selected from this. The mutants were then inbred to produce the G1 generation, which was diagnosed using the PCR marker. The selected homozygote mutants were inbred to make progenies and this G2 generation was used for subsequent physiological and molecular analyses.

### 2.12. RNA-sequencing (RNASeq) of the CRISPR mutant for transcriptome analysis

There were four treatments that consisted of a combination of CRISPR mutagenesis and immune challenge. Each treatment was replicated three times. The resulting 12 samples were subject to RNASeq. To obtain short-read RNA sequences, Illumina sequencing was performed at Macrogen (Seoul, Korea). Each library was constructed from 1 μg of total RNA from the fat body of five L5 larvae per treatment (S1 Table) using the TruSeq stranded Library Preparation kit (Illumina, San Diego, USA) and sequenced using the Illumina NovaSeq 6000 instrument with a 101 bp pair end read.

Illumina short reads were quality-filtered and adapter-trimmed using Trimmomatic 0.38 (http://www.usadellab.org/cms/?page=trimmomatic). FastQC v0.11.7 (http://www.bioinformatics.babraham.ac.uk/projects/fastqc/) was used to check data quality before and after trimming. After removing low-quality reads, an Illumina-based *de novo* transcriptome assembly was performed using Trinity version trinityrnaseq_r20140717, bowtie 1.1.2. Trimmed reads for all fat body samples were merged into one file to construct the combined reference. The *de novo* assembly of merged data was carried out using Trinity with default parameters such that it was assembled into transcript contigs. The total number of genes, transcripts, GC content max/min/median/average contig length, and total assembled bases were summarized. Trinity groups transcripts into clusters based on shared sequence content. The assembled contigs are filtered and selected to obtain the non-redundant transcripts using CD-HIT version 4.6 (http://weizhongli-lab.org/cd-hit). These transcripts were defined as ‘unigenes’, which are used to predict open reading frames (ORFs), annotate against several known sequence databases, and analyze differentially expressed genes (DEGs). ORF prediction for unigenes was performed using TransDecoder version 3.0.1 (https://github.com/TransDecoder/TransDecoder/wiki) to identify candidate coding regions within the transcript sequence. After extracting ORFs that were at least 100 amino acids long, the TransDecoder program was used to predict the likely coding regions. Trimmed reads for each sample were aligned to the assembled reference using the Bowtie program. For differentially expressed gene analysis, the abundances of unigenes across samples were estimated into read count as an expression measure using the RSEM algorithm (RSEM version v1.2.29, bowtie 1.1.2, http://deweylab.github.io/RSEM/). For functional annotation, unigenes were searched against the Kyoto Encyclopedia of Genes and Genomes (KEGG) v 20190104 (http://www.genome.jp/kegg/ko.html), NCBI Nucleotide (NT) v20180116 (https://www.ncbi.nlm.nih.gov/nucleotide/), Pfam v20160316 (https://pfam.xfam.org/), Gene ontology (GO) v20180319 (http://www.geneontology.org/), NCBI non-redundant Protein (NR) v20180503 (https://www.ncbi.nlm.nih.gov/protein/), UniProt v20180116 (http://www.uniprot.org/), and EggNOG (http://eggnogdb.embl.de/) using BLASTN of NCBI BLAST and BLASTX of DIAMOND version 0.9.21 (https://github.com/bbuchfink/diamond) with an E-value default cutoff of 1.0E-5.

### 2.13. DEG analysis

Contigs were collected from all 12 samples (4 treatments x 3 replications). If more than one read count value was 0, it was not included in the analysis. Therefore, from a total of 174,839 contigs, 157,312 were excluded, and 17,527 contigs were used for statistical analysis. To reduce systematic bias, the size factors were estimated from the count data and normalized by relative Log expression with the DESeq2 R library. Using each sample’s normalized value, the high expression similarities were grouped together by a hierarchical clustering analysis and graphically shown in a 2D plot to depict the variability of the total data using a multidimensional scaling analysis. Significant unigenes were analyzed as up/down-regulated count based on the fold change. A heatmap was prepared using GraphPad Prism version 8.2.0 (La Jolla, CA, USA).

### 2.14. Nodulation assay

A nodulation assay was performed at 24 h after IVTE (1 μg larva-1) treatment. Briefly, heat-killed *X*. *nematophila* (5 × 10^4^ cells per larva) was injected into larvae through an abdominal proleg. The treated larvae were incubated for 8 h at 25°C, after which they were dissected to count nodules in the hemocoel. The melanized nodules were counted on gut and fat body under a stereoscopic microscope (Stemi SV11, Zeiss, Jena, Germany) at 50× magnification. Each treatment used 10 larvae, and each treatment was independently replicated three times.

### 2.15. Developmental and fecundity tests

Wild type and mutant insects were compared in pupal weight and pupation rate. The larvae in both treatments were reared in standard laboratory conditions with artificial diet. Pupation rate and pupal weight were measured on the first day after pupation. Each treatment was replicated three times, and each replicate consisted of 10 insects. Upon the adult emergence, male and female adults were housed together for mating. The total numbers of eggs laid were counted for 3 days. Each treatment used five pairs and was replicated three times.

### 2.16. Data analysis

All data are presented as the results of three independent biological replicates. Data were plotted using Sigma plot 10.0. Means were compared by least squared difference (LSD) test of one-way analysis of variance (ANOVA) using PROC GLM of SAS program [53] and discriminated at Type I error = 0.05.

## 3. Results

### 3.1. Positive correlation between *Se-sPLA*_*2*_ expression and its enzyme activity

**Fig 1:**
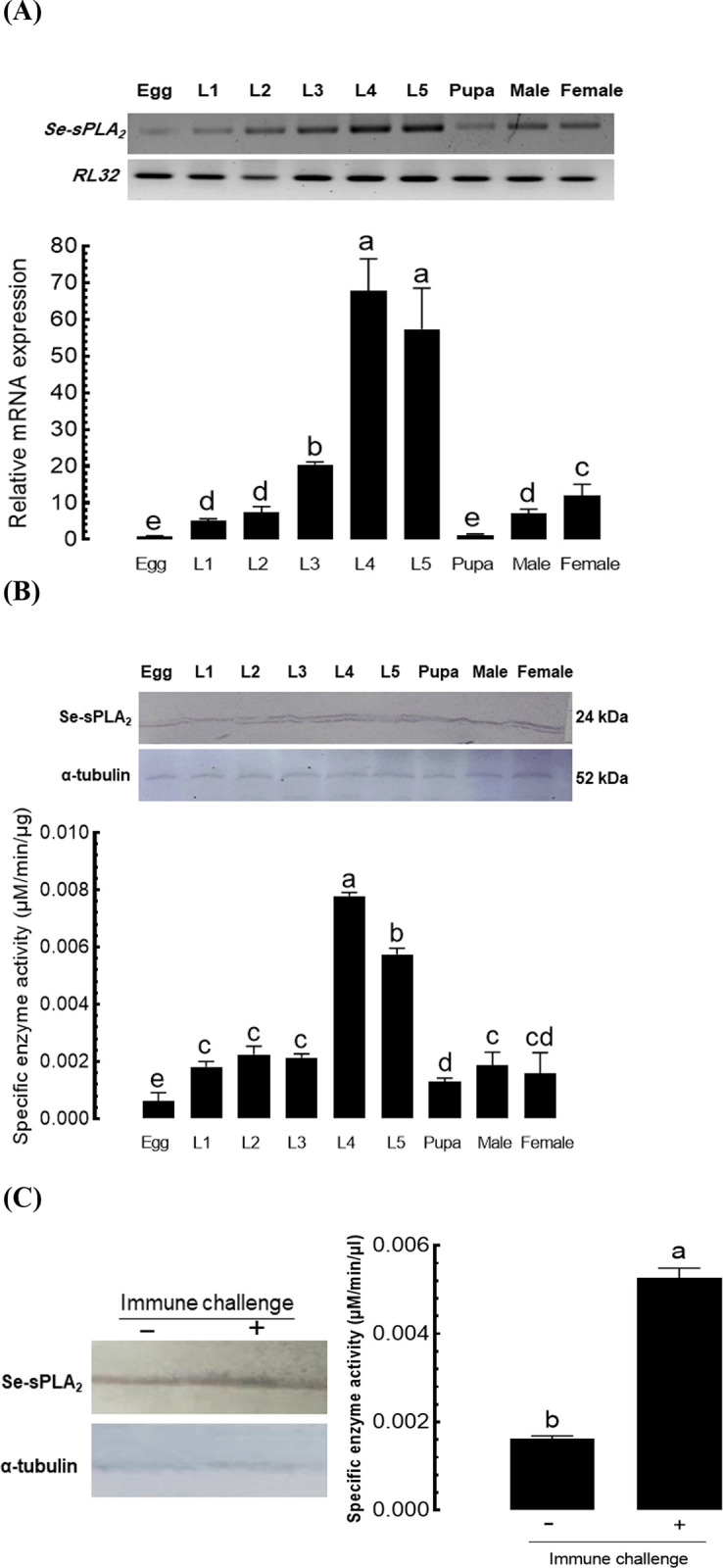
Variation in gene (*Se-sPLA*_*2*_) expression and enzyme activity of sPLA_2_ in different developmental stages of *S*. *exigua*. (A) Expression profile of *Se-sPLA*_*2*_ in different stages using RT-PCR (upper panel) and RT-qPCR (lower panel). A ribosomal protein, *RL32*, expression was used for control for RT-PCR and normalization for RT-qPCR. (B) Its enzyme activities from the whole bodies of different stages, in which the upper panel shows a western blot. Monoclonal antibody specific to α-tubulin was used for control to confirm the same amount protein loading. (C) Up-regulation in sPLA_2_ activity in the hemolymph upon an immune challenge with 5 × 10^4^ *X*. *nematophila* killed at 95°C for 5 min. Left panel shows the Se-sPLA_2_ protein level in the plasma by a western blot. All treatments were replicated three times. Different letters above the standard deviation bars indicate the significant difference among means at Type I error = 0.05 (LSD test).

Our RT-PCR results show that *Se-sPLA*_*2*_ was expressed in all developmental stages (Fig 1A) and that its expression levels significantly varied among them. Its translated protein was also detected in all developmental stages (Fig 1B) and the specific enzyme activities significantly varied among them, particularly in L4 and L5. The expression levels measured by RT-qPCR were positively correlated (*r* = 0.9760; *P* < 0.0001) with the enzyme activities among developmental stages. Under this positive correlation, an immune challenge led to increased Se-sPLA_2_ protein level, shown on an immunoblot and it led to significant up-regulation of the enzyme activity (Fig 1C).

### 3.2. CRISPR target site for *Se-sPLA*_*2*_ on *S*. *exigua* genome

**Fig 2:**
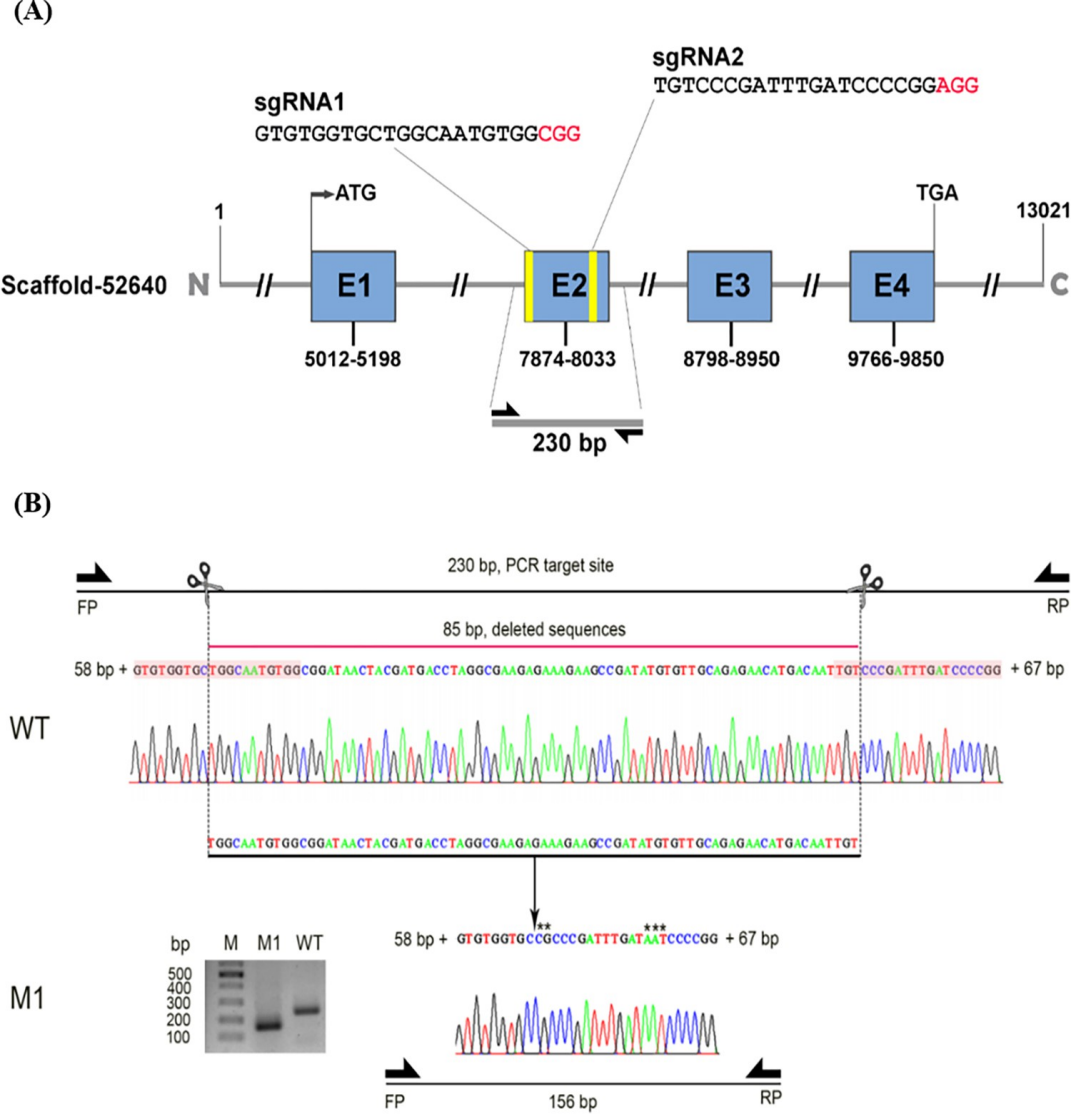
Mutagenesis using CRISPR/Cas9 against *Se-sPLA*_*2*_. (A) Design of single stranded guide RNAs (sgRNA1 and sgRNA2) from exon 2 (E2) on the genomic DNA of *Se-sPLA*_*2*_. The genome map (scaffold 52640) was obtained from GenBank accession number of GCA_022117675.1. Two sgRNAs are 85 bp apart based on their cleavage sites. (B) Demonstration of CRISPR mutagenesis using two sgRNAs and Cas9. Two arrows indicate diagnostic primers (FP and RP), which produce 230 bp PCR product including 58 bp and 67 bp neighboring the cleavage sites. In contrast to wild type (WT), a mutant (M1) produced 156 bp by 85 bp loss and some indels.

The section 3.1 result indicates that sPLA_2_ enzyme activity is regulated by the gene expression. This suggests that any *Se-sPLA*_*2*_ knock-out mutant, a unique sPLA_2_ gene in the genome [28] would result in reduced enzyme activity. An interrogation of the genome database (GenBank accession number: GCA_022117675.1) with the sPLA_2_ cDNA sequence (GenBank accession number: MH061374) identified its genomic DNA scaffold (#52640). The *Se-sPLA*_*2*_ genomic DNA (13,021 bp) was predicted to have four exons ranging from 84 to 186 bp (Fig 2A). The nucleotide sequence of the second exon (‘E2’, 160 bp) was selected for the target of CRISPR/Cas9 mutagenesis and used to design two sgRNA, separated by 85 bp. To confirm the mutagenesis, PCR primers were designed to amplify the region (230 bp) covering the CRISPR target site (Fig 2B). Indeed, one mutant (‘M1’) produced 156 bp by loss of the 85 bp and some indels at the cleavage sites compared to wild type (WT) (see the inset gel photo).

### 3.3. Genome editing in Se-sPLA_2_ using CRISPR/Cas9

**Fig 3:**
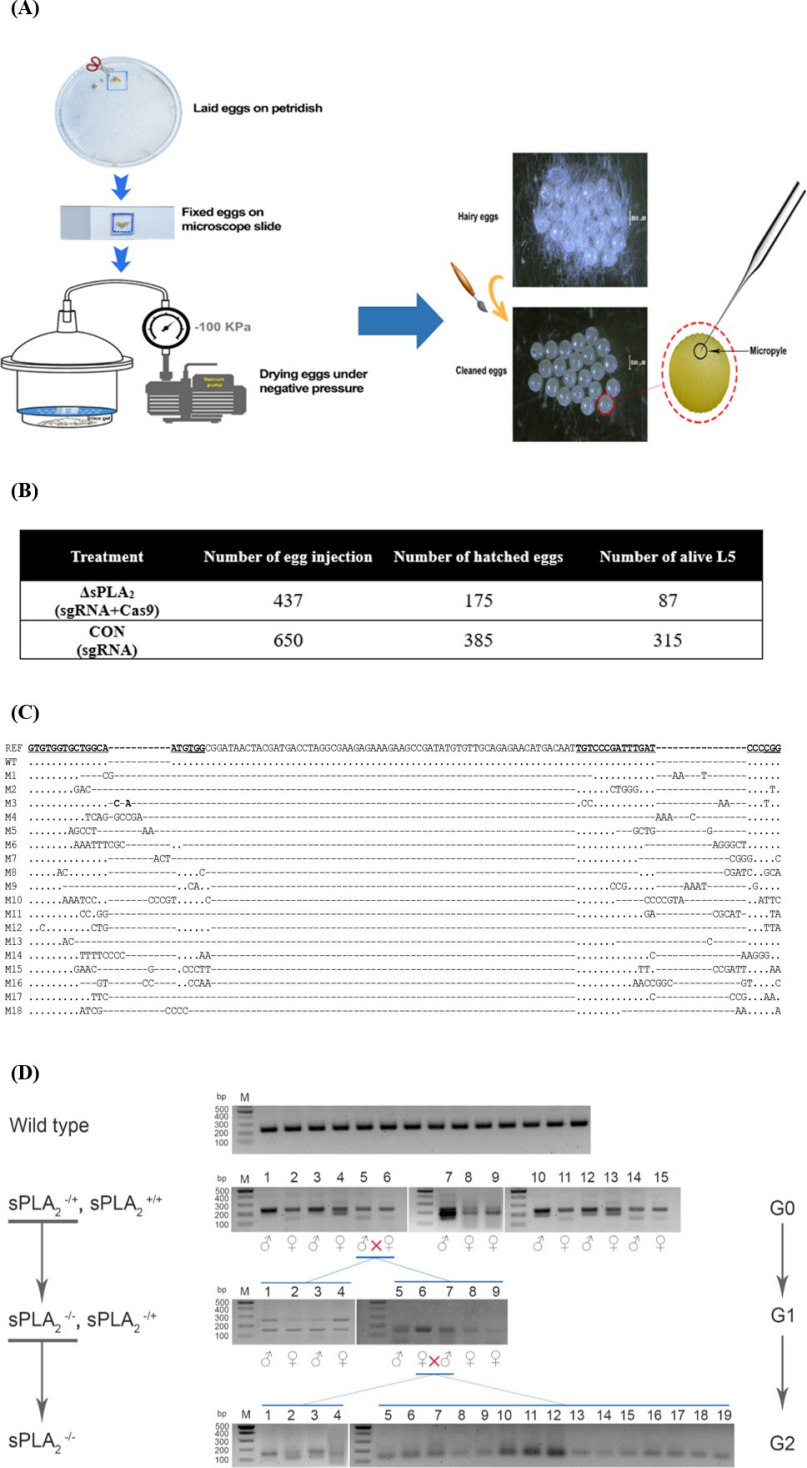
Construction of the deletion mutants in Se-sPLA_2_ of *S*. *exigua* using CRISPR/Cas9. (A) Preparation of the newly laid eggs for microinjection of sgRNAs and Cas9. (B) Outcomes of the mutagenesis. Mutagenic treatment (ΔsPLA_2_) used both sgRNA and Cas9 for the injection while the control (CON) used only sgRNA. After CRISPR mutagenesis, the final live larvae were counted at fifth instar (L5). (C) Confirmation of the mutagenesis by sequencing at the target sites (230 bp) produced by diagnostic primers. All 18 mutants (M1-M18) were obtained based on the sequence comparison with the wild type (WT) sequence and reference (REF) sequence reported in GenBank (MH061374). (D) Selection of homozygote (-/-) mutants. At the first generation (G0), all the mutants were heterozygote (-/+). These inbred line (5 × 6 male and female pair) gave the homozygote mutants at the next generation (G1). The homozygotes (6 × 7 male and female pair) were inbred to get all homozygote mutants at G2 generation.

The newly laid eggs were used to inject a mixture of sgRNA and Cas9 (Fig 3A). The dehydrated eggs were injected with a ~5 nL volume through a micropyle (= a pore for sperm entry during fertilization) on the top of the egg. The control injection included the same volume of solution without Cas9 protein. A slightly lower hatching rate (40.0%) was recorded in the mutagenic treatment compared to the control (59.2%; Fig 3B). The mutagenic treatment led a much lower survival rate (49.7%) than the control (81.8%). To investigate mutations, genomic DNA was extracted from all 87 larval exuviae, and PCR was performed specifically at the 230 bp CRISPR target region. When the sizes of the PCR products of 87 individuals were examined, 18 samples contained their products near the expected mutant size (145 bp). These mutant PCR products were sequenced and aligned with non-mutant (= wild) sequence (Fig 3C). The sequence data of 18 mutant individuals showed that Cas9 cut the target DNA at the sgRNA-binding site, where nucleotide sequences showed substantial variation. The deletion sizes varied among 18 mutant individuals from 80 bp (M5) and 90 bp (‘M17’). To select mutants, single pair matings of mutants were performed (Fig 3D). Two heterozygote mutants (‘M5’ and ‘M6’) were crossed at the G0 generation, and these produced the mixed progenies of wild and mutant types in the G1 generation. Two homozygotes, a female (‘M6’) and a male (‘M7’), were then selected and crossed together to produce the G2 generation. All the progeny in G2 were homozygotes in the deletion mutant genotype because the 19 randomly chosen larvae turned out to be homozygous in the mutant genotype.

### 3.4. RNASeq of the CRISPR mutant and wild types upon immune challenge

**Fig 4:**
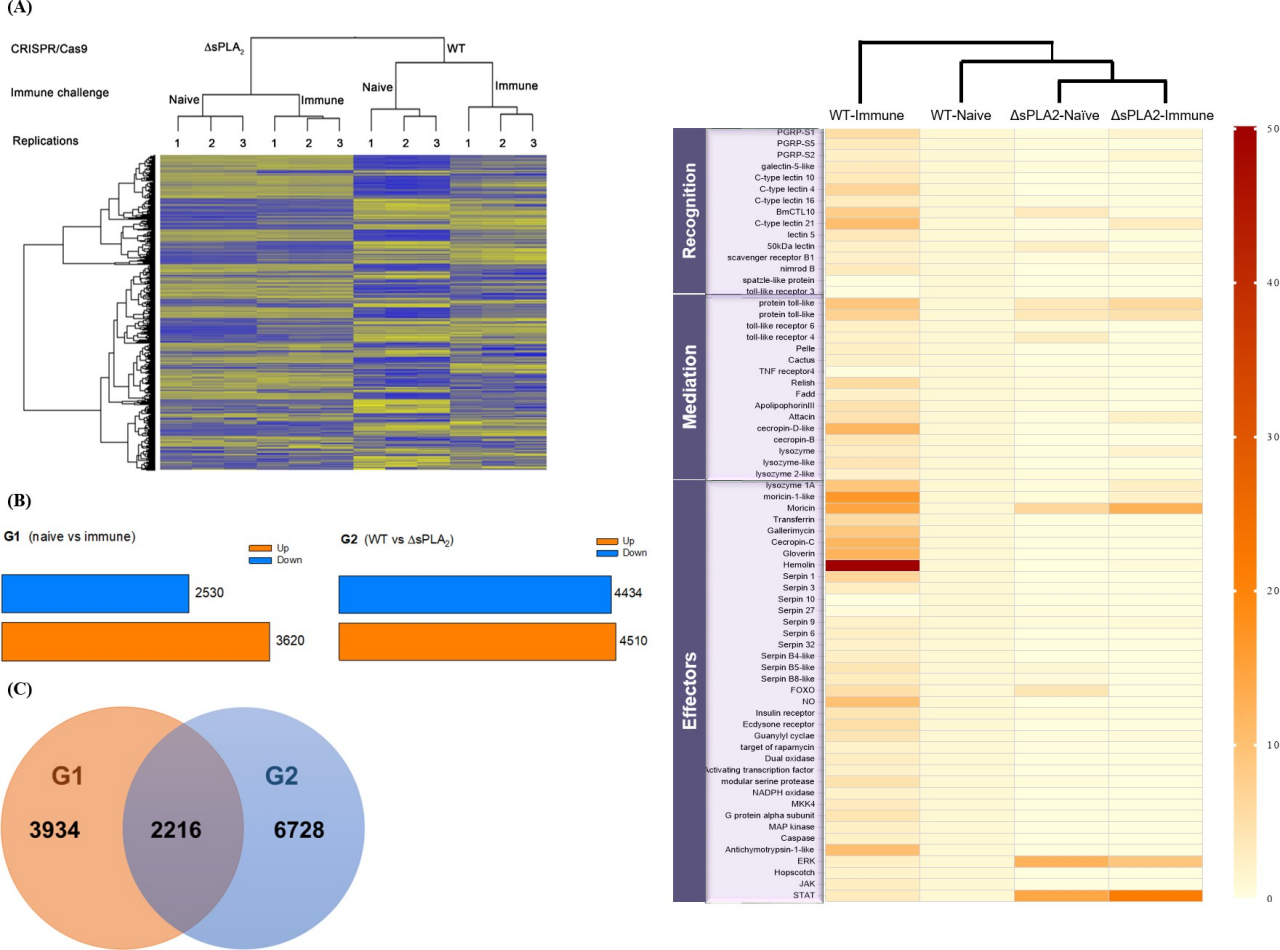
RNASeq analysis of CRISPR mutants (ΔsPLA_2_) of *S*. *exigua* in *Se-sPLA*_*2*_. Both mutant wild type (WT) larvae were immune challenged with 5 × 10^4^ *X*. *nematophila* killed at 95°C for 5 min. These four treatments are WT-naïve, WT-immune, ΔsPLA_2_-naïve, and ΔsPLA_2_-immune. RNASeq was performed three replications in each treatment. (A) Hierarchical clustering analysis of 12,878 unigenes among 12 RNASeq samples. (B) Differentially expressed genes (DEGs) selected from two comparison groups (G1 and G2). DEGs of G1 represent immune-associated genes by comparing transcripts of naïve and immune challenge in WT. DEGs of G2 represent the target genes associated with *sPLA*_*2*_ expression by comparing wild type and mutant transcripts. (C) Venn diagram to indicate the immune-associated genes controlled by *sPLA*_*2*_ expression. (D) Heatmap analysis depicting the expression profiles of 55 canonical immune-associated genes among four treatments. A phylogeny analysis was obtained using ClustVis online tool (https://biit.cs.us.ee/clustvis/). It indicates little responsiveness of the CRISPR mutant to the immune challenge in the canonical immune genes compared to wild type larvae.

The homozygote mutant in sPLA_2_ was referred to as ΔsPLA_2_ compared to WT. To determine the immunity-associated genes related to *sPLA*_*2*_ expression, larvae of ΔsPLA_2_ and WT were subject to immune challenge and called ΔsPLA_2_-immune and WT-immune, respectively. The corresponding controls without immune challenge were respectively called ΔsPLA_2_-naïve and WT-naïve. These four groups were subject to RNASeq analysis with three replications (Fig 4A). The raw read sizes were 10.6–14.7 Gb among four different treatments (S2 Table). After trimming, the clean reads were mapped to the genome in a range from 74.6 to 87.5% among four treatments. The clean reads were assembled to generate contigs with average sizes of 511~611 bp, and 174,839 unigenes were predicted in total (S3 Table). For DEG analysis, 157,312 unigenes were excluded because of a lack of expression in at least one replication, while the remaining 12,878 unigenes were further analyzed.

### 3.5. Transcriptome analysis of a deletion mutant in ΔsPLA_2_

The four treatments were compared in terms of their expression profiles on 12,878 unigenes (Fig 4A). The results of the clustering analysis showed that the four different treatments were separated in their expression profiles. In particular, the mutant groups (ΔsPLA_2_ with or without immune challenge) were found to be distinct from the wild type (WT with or without immune challenge). To assess the effect of immune challenge, WT-naïve and WT-immune were compared (G1 comparison), and the results showed that 6,150 DEGs (3,620 up-regulated genes and 2,530 down-regulated genes) showed over two-fold alterations in their expression levels (Fig 4B). S4 Table lists the DEGs that were highly regulated by > 10 folds. To assess the mutagenesis effect, WT-naïve and ΔsPLA_2_–naïve were compared (G2 comparison), which showed that 8,944 DEGs (4,510 up-regulated genes and 4,434 down-regulated genes) were altered in their expression levels by over two folds. S5 Table lists these DEGs that were regulated by > 10 folds. The genes controlled by *sPLA*_*2*_ expression were annotated using KEGG analysis (Table 1) and functionally categorized into metabolism, genetic information processing, environmental information processing, cellular processes, and organismal systems. To determine the genes that were associated with immunity controlled by *sPLA*_*2*_ expression, G1 and G2 DEGs were compared (Fig 4C). In total, 2,216 genes were overlapped between these two DEGs, including 1,203 down-regulated genes and 1,013 up-regulated genes by deletion mutagenesis upon the immune challenge. Among the DEGs exhibiting more than a 10-fold change in FPKM (S6 Table), the expression level of *anionic antimicrobial peptide 2* (GenBank accession number = XP_022826185.1) was found to decrease 58.9-fold when compared between WT and the mutant upon immune challenge. By contrast, *serpin* (XP_022837525.1), which suppresses immune responses mediated by serine proteases, increased 695.1-fold in the mutant.

**Table 1:** KEGG analysis of 8,944 DEGs (G2 in Fig 4B) associated with *sPLA*_*2*_ expression in *S*. *exigua*.

Category	Subgroup	Metabolic pathway	Total	Genes (> 2 folds)		
				Up	Down	Neutral
**Metabolism**	**Carbohydrate metabolism**	Glycolysis / Gluconeogenesis	43	5	15	23
		Citrate cycle (TCA cycle)	33	2	6	25
		Pentose phosphate pathway	27	2	12	13
		Pentose and glucuronate interconversions	42	4	18	20
		Fructose and mannose metabolism	27	3	14	10
		Galactose metabolism	26	4	12	10
		Ascorbate and aldarate metabolism	33	5	17	11
		Starch and sucrose metabolism	17	6	5	6
		Amino sugar and nucleotide sugar metabolism	37	5	14	18
		Pyruvate metabolism	37	4	10	23
		Glyoxylate and dicarboxylate metabolism	35	3	16	16
		Propanoate metabolism	34	5	11	18
		Butanoate metabolism	13	1	6	6
		Inositol phosphate metabolism	50	33	6	11
	**Energy metabolism**	Oxidative phosphorylation	185	4	118	63
		Nitrogen metabolism	13	4	5	4
		Sulfur metabolism	12	0	4	8
	**Lipid metabolism**	Fatty acid biosynthesis	18	6	3	9
		Fatty acid elongation	33	3	12	18
		Fatty acid degradation	33	3	13	17
		Synthesis and degradation of ketone bodies	4	0	3	1
		Steroid biosynthesis	12	5	7	0
		Glycerolipid metabolism	53	16	20	17
		Glycerophospholipid metabolism	79	33	19	27
		Ether lipid metabolism	24	12	4	8
		Sphingolipid metabolism	30	8	7	15
		Arachidonic acid metabolism	26	7	12	7
		Linoleic acid metabolism	9	6	2	1
		alpha-Linolenic acid metabolism	17	9	2	6
		Biosynthesis of unsaturated fatty acids	27	4	14	9
	**Nucleotide metabolism**	Purine metabolism	112	28	34	50
		Pyrimidine metabolism	33	8	8	17
	**Amino acid metabolism**	Alanine, aspartate and glutamate metabolism	29	4	12	13
		Glycine, serine and threonine metabolism	29	0	21	8
		Cysteine and methionine metabolism	40	3	12	25
		Valine, leucine and isoleucine degradation	44	1	20	23
		Valine, leucine and isoleucine biosynthesis	-	ND	ND	ND
		Lysine degradation	37	8	15	14
		Arginine biosynthesis	22	1	13	8
		Arginine and proline metabolism	27	1	11	15
		Histidine metabolism	9	1	4	4
		Tyrosine metabolism	31	3	25	3
		Phenylalanine metabolism	11	0	7	4
		Tryptophan metabolism	33	4	19	10
		Phenylalanine, tyrosine and tryptophan biosynthesis	7	0	4	3
		beta-Alanine metabolism	21	2	9	10
		Taurine and hypotaurine metabolism	6	3	1	2
		Phosphonate and phosphonate metabolism	5	1	0	4
		Selenocompound metabolism	9	2	1	6
		D-Glutamine and D-glutamate metabolism	3	0	1	2
		D-Arginine and D-ornithine metabolism	2	0	2	0
		Glutathione metabolism	35	7	24	14
	**Glycan biosynthesis and metabolism**	N-Glycan biosynthesis	53	26	11	16
		Mucin type O-glycan biosynthesis	21	16	0	5
		Mannose type O-glycan biosynthesis	16	8	3	5
		Other types of O-glycan biosynthesis	11	6	3	2
		Glycosaminoglycan biosynthesis—chondroitin sulfate / dermatan sulfate	14	5	3	6
		Glycosaminoglycan biosynthesis—heparan sulfate / heparin	14	6	0	8
		Glycosaminoglycan biosynthesis—keratan sulfate	2	1	1	0
		Glycosaminoglycan degradation	27	6	0	21
		Glycosylphosphatidylinositol (GPI)-anchor biosynthesis	16	4	1	11
		Glycosphingolipid biosynthesis—lacto and neolacto series	4	0	2	2
		Glycosphingolipid biosynthesis—globo and isoglobo series	10	2	2	6
		Glycosphingolipid biosynthesis—ganglio series	10	1	0	9
		Other glycan degradation	29	11	3	15
	**Metabolism of cofactors and vitamins**	Thiamine metabolism	17	5	8	4
		Riboflavin metabolism	6	1	3	2
		Vitamin B6 metabolism	4	0	2	2
		Nicotinate and nicotinamide metabolism	16	3	3	10
		Pantothenate and CoA biosynthesis	12	5	2	5
		Biotin metabolism	5	4	0	1
		Lipoic acid metabolism	3	0	1	2
		Folate biosynthesis	31	10	20	11
		One carbon pool by folate	14	0	5	9
		Retinol metabolism	21	4	5	12
		Porphyrin and chlorophyll metabolism	31	12	6	13
		Ubiquinone and other terpenoid-quinone biosynthesis	10	1	2	7
	**Metabolism of terpenoids and polyketides**	Terpenoid backbone biosynthesis	30	8	8	14
		Insect hormone biosynthesis	21	3	11	7
	**Biosynthesis of other secondary metabolites**	Caffeine metabolism	12	1	10	1
	**Xenobiotics degradation and metabolism**	Metabolism of xenobiotics by cytochrome P450	37	2	24	11
		Drug metabolism—cytochrome P450	33	5	20	8
		Drug metabolism—other enzymes	60	7	28	25
**Genetic Information Processing**	**Transcription**	RNA polymerase	28	7	2	19
		Basal transcription factors	28	8	2	18
		Spliceosome	108	32	0	76
	**Translation**	Ribosome	124	0	33	91
		Aminoacyl-tRNA biosynthesis	36	15	3	18
		RNA transport	65	3	4	59
		mRNA surveillance pathway	59	21	1	37
		Ribosome biogenesis in eukaryotes	64	23	3	38
	**Folding, sorting and degradation**	Protein export	22	1	10	11
		Protein processing in endoplasmic reticulum	118	32	26	60
		SNARE interactions in vesicular transport	20	2	4	14
		Ubiquitin mediated proteolysis	78	22	4	52
		Sulfur relay system	9	3	0	6
		Proteasome	32	1	1	30
		RNA degradation	67	10	2	55
	**Replication and repair**	DNA replication	35	14	3	18
		Base excision repair	23	6	1	16
		Nucleotide excision repair	30	11	1	18
		Mismatch repair	16	4	1	11
		Homologous recombination	30	12	1	17
		Non-homologous end-joining	7	4	0	3
		Fanconi anemia pathway	30	13	2	15
**Environment Information Processing**	**Membrane transport**	ABC transporters	23	14	3	6
	**Signal transduction**	MAPK signaling pathway—fly	75	31	10	34
		Wnt signaling pathway	54	24	12	28
		Notch signaling pathway	21	11	1	9
		Hedgehog signaling pathway—fly	29	12	3	14
		TGF-beta signaling pathway	33	15	3	15
		Hippo signaling pathway—fly	53	23	8	22
		Hippo signaling pathway—multiple species	40	15	6	19
		FoxO signaling pathway	55	23	5	27
		Phosphatidylinositol signaling system	47	32	1	14
		mTOR signaling pathway	87	32	25	30
	**Signaling molecules and interaction**	Neuroactive ligand-receptor interaction	39	10	22	7
		ECM-receptor interaction	27	10	4	13
**Cellular Processes**	**Transport and catabolism**	Endocytosis	110	47	2	61
		Phagosome	62	15	17	30
		Lysosome	95	24	34	37
		Peroxisome	97	21	42	34
		Autophagy—animal	79	38	9	32
		Autophagy—other	21	10	0	11
		Mitophagy—animal	31	20	0	11
	**Cell growth and death**	Apoptosis—fly	47	25	5	17
		Apoptosis—multiple species	24	7	3	14
**Organismal Systems**	**Immune system**	Toll signaling pathway	15	3	3	9
		Imd signaling pathway	22	6	3	13
	**Sensory system**	Phototransduction	21	7	4	10
	**Development and regeneration**	Dorso-ventral axis formation	22	14	1	7
	**Aging**	Longevity regulating pathway—multiple species	100	32	36	32
	**Environment adaptation**	Circadian rhythm—fly	8	5	1	2

To further analyze the deletion mutant in immune function, the canonical immune genes were selected in three categories: 13 genes in recognition, 13 genes in mediation, and 33 genes in effectors. Their FPRK values were compared among four treatments: WT-naïve, WT-immune, ΔsPLA_2_-naïve, and ΔsPLA_2_-immune (S1 Fig). These expression patterns were illustrated in a heatmap (Fig 4D). The immune challenge up-regulated most of the immune genes in WT larvae, while the mutant larvae failed to increase the immune genes upon immune challenge in all three categories. This is further supported by a phylogeny tree clustering the similar expression patterns of the four treatments, in which the immune-challenged mutant line (‘ΔsPLA_2_-immune’) was co-clustered with naïve WT line (‘WT-naïve’).

### 3.6. Biological characters of the deletion mutant in ΔsPLA_2_

**Fig 5:**
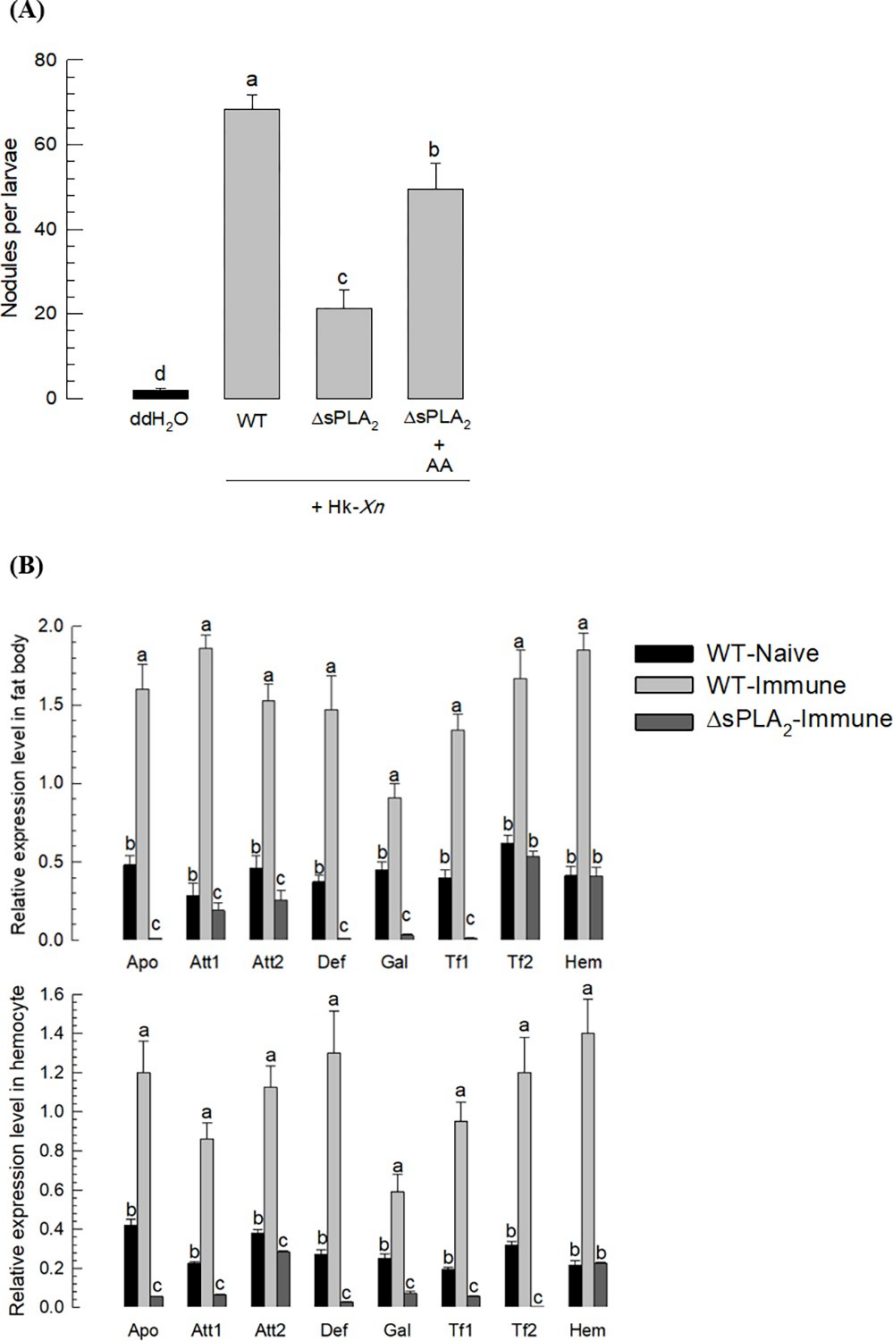
Suppression in immune responses of the CRISPR mutants (ΔsPLA_2_) in *Se-sPLA*_*2*_. (A) Suppression in a cellular immune response assessed by nodule formation. L5 larvae were injected with 5 × 10^4^ cells of Heat-killed *X*. *nematophila* (HK-Xn). After 8 h, the larvae were dissected for counting nodules. Each treatment was replicated with 10 larvae. Arachidonic acid (AA) was injected to the larvae 1 μg per larvae. (B) Suppression in humoral immune response. In two immune-associated tissues, eight AMP genes were assessed in RT-qPCR after immune challenge: Apo (apolipophorin III), Att1 (attacin 1), Att2 (attacin 2), Def (defensing), Gal (gallerimycin), Tf1 (transferrin 1), Tf2 (transferrin 2), and Hem (hemolin). Different letters above the standard deviation bars indicate significant difference among means in each AMP at Type I error = 0.05 (LSD test).

The *sPLA*_*2*_ mutant showed a loss of immune responses to the bacterial infection (Fig 5). In a cellular response, nodule formation in the mutant was severely reduced compared to the WT because the mutant showed the marked decrease in the nodule formation (about 20 nodules/larva) compared to about 68 nodules/larva in the control wild type (Fig 5A). However, the addition of arachidonic acid, a catalytic product of sPLA_2_, significantly rescued the nodule formation, up to about 48 nodules/larvae. Humoral immunity was analyzed by assessing the expression levels of eight antimicrobial peptides (AMPs) in two larval tissues associated with immunity (Fig 5B). All these AMP genes were highly upregulated upon the bacterial infection. However, the mutant larvae did not up-regulate expression of the AMP genes in response to infection.

**Fig 6:**
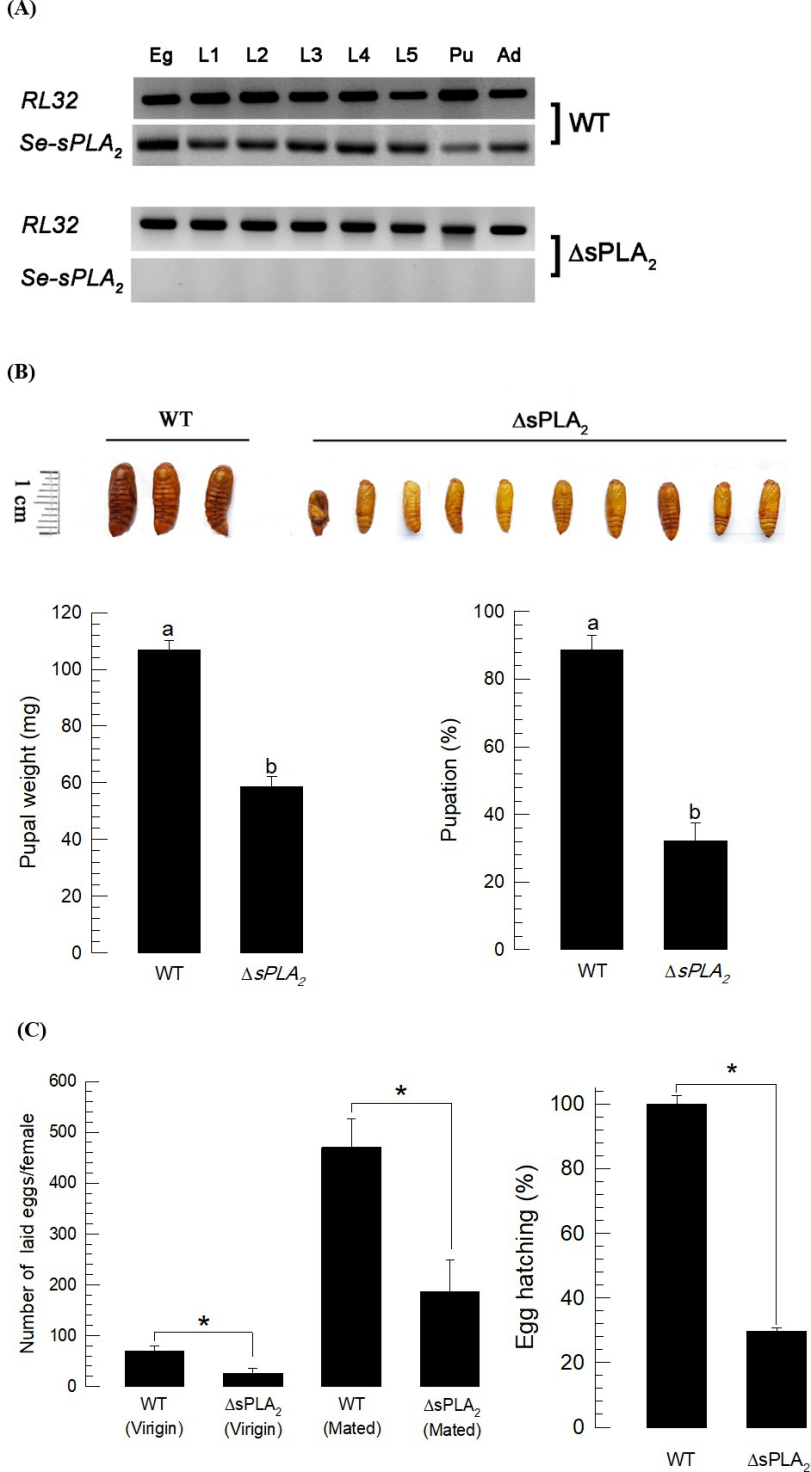
Developmental and reproductive alterations of the CRISPR mutants (ΔsPLA_2_) in *Se-sPLA*_*2*_. (A) Expression analysis of *Se-sPLA*_*2*_ in mutant and wild type (WT) during entire developmental stages of *S*. *exigua*. (B) Alteration in immature development. Reduced pupal size (*n* = 10) and pupation percentage. Pupal weight was measured within 10 h after pupation. (C) Alteration in reproduction assessed by fecundity (egg laying number) and fertility (egg hatch). Different letters or asterisk above the standard deviation bars indicate significant difference among means at Type I error = 0.05 (LSD test).

The *sPLA*_*2*_ mutant exhibited abnormal immature development and adult fecundity (Fig 6). As expected, throughout all developmental stages, *sPLA*_*2*_ was expressed in WT larvae but not in the mutant (Fig 6A). This mutation led to malformed or hypotrophied pupae, seen as severely reduced pupal weights and reduced pupation compared to WT larvae (Fig 6B). In WT females, mating significantly stimulated egg-laying behavior (Fig 6C), which did not occur in the PLA_2_ mutants. Further, the progeny from the mutant females suffered severely reduced egg hatching, down from 100% in eggs from WTs to about 28% in the PLA_2_ mutants.

## 4. Discussion

Eicosanoids make up a substantial group of functional oxylipins. They were first identified in human biomedicine. Over the past few decades a wide range of discoveries revealed the broad biological significance of prostaglandins (PGs) and other eicosanoids in insects and other invertebrates, particularly in immunology [2, 3, 22]. They are typically biosynthesized from arachidonic acid, which is often released from membrane phospholipids by the catalytic activity of various PLA_2_s. To understand the molecular actions of the eicosanoids associated with the various physiological processes, we used a loss-of-function strategy through knocking-out a gene encoding PLA_2_ from the germline using the CRISPR/Cas9 technique. Here, we prepared a *Se-sPLA*_*2*_ deletion mutant, which is a unique sPLA_2_ in *S*. *exigua*, and monitored the expressed genes by RNASeq to determine the target genes influenced by the mutagenesis.

Mutagenesis using two sgRNAs deleted 80–90 bp in exon 2 of sPLA_2_ from the genomic DNA of *S*. *exigua*. These deletion mutants were expected, because it was estimated by 85 bp between the two sgRNA target sites, at which the Cas9 nuclease activity would occur three bases away from the protospacer adjacent motif–NGG (PAM) and ultimately result in a double-strand break [54]. Our CRISPR demonstration supported the expectation of the deletion size. After Cas9 cleavage at 3 bp away from PAM, DNA repair to re-join the breaks would occur due to cell machinery in an error-prone non-homologous end joining manner, thus leading to small insertion, deletion, or nucleotide substitutions [55]. These results suggest that the CRISPR/Cas9 gene editing system can be used as a potential tool to produce heritable mutations in *S*. *exigua* as well as other non-model insects [56]. However, the CRISPR/Cas9 mutants at the first generation were all heterozygotes, where only one allele was mutated. Subsequent selective inbreeding resulted in 100% homozygote mutants which were then used for subsequent RNASeq and selected bioassays.

In WT larvae, we found in our current RNASeq, over 47% of the expressed genes were differentially expressed after bacterial infection. This estimate, which was analysed by the Illumina HiSeq platform, was slightly higher than the previous record (36%) assessed by 454 pyrosequencing platform in the same species [57]. However, DEGs obtained from both RNASeq techniques share the same functional categories in immunity, classified into recognition, signal mediation, and effectors. Based on the expression profiles derived from the immune-effector tissues, Gunaratna and Jiang [58] determined a repertoire of 232 immunity-related gene-encoding proteins for pathogen recognition (16%), signal transduction (53%), microbe killing (13%), and other purposes (18%). This functional refinement of immune-associated genes supports our view that the DEGs of the current assays include the genetic factors associated with immunity of *S*. *exigua*.

The *Se-sPLA*_*2*_ deletion mutant showed differential expression patterns in 8,944 genes, and these were assorted in most cellular processes, including immunity. Even though *S*. *exigua* expresses two other types of PLA_2_ genes, *Se-iPLA*_*2*_*A* and *Se-iPLA*_*2*_*B* [59, 60] the *Se-sPLA*_*2*_ deletion mutant must lead to reduced eicosanoid biosynthesis. Thus, over 69% of the expressed genes would be influenced in their expression either directly or indirectly by eicosanoids. A unique eicosanoid receptor specific to PGE_2_ was identified in *S*. *exigua* [61]. PGE_2_ binding to the receptor triggers intracellular signalling via cAMP and Ca^2+^ [36]. This suggests that the downstream signal of the secondary messengers induces the expression of a number of specific genes, expected to be associated with various physiological processes. Further, *REPAT33* is a downstream gene induced by eicosanoids in *S*. *exigua* [62]. REPAT33 is a transcriptional factor that regulates other target genes associated with immunity [63].

In this paper, immune-associated genes modulated by eicosanoids were determined by comparative transcriptome analysis between the immune-associated DEGs from WT and the CRISPR mutant specific DEGs. The results of this analysis showed that eicosanoids influenced about 36% (= 2,216 genes) of the immune-associated genes. Thus, the *Se-sPLA*_*2*_ deletion mutant did not induce the expression of most canonical immune-associated genes, which are classified into recognition, mediation, and effector. The mutant was also impaired in terms of cellular and humoral immune responses against the entomopathogenic bacterial infection. However, the addition of arachidonic acid (AA), a catalytic product of sPLA_2_, significantly reversed the immunosuppression. AA is used for the biosynthesis of different eicosanoids, which mediate the immune responses [9, 22].

The sPLA_2_-mutant suffered from immature mortality and poor development. Aside from the broad range of physiological actions mediated by eicosanoids, sPLA_2_ plays a crucial role in lipid digestion [64]. An inhibitor specific to sPLA_2_ significantly inhibited larval development by suppressing digestion efficiency in *S*. *exigua* [65]. This indicates that Se-sPLA_2_ is secreted into the midgut lumen to play act in digestion of dietary lipids. RNAi against Se-sPLA_2_ resulted in reduced digestibility, which was then significantly rescued by the addition of a catalytic product—1-palmitoyl-*sn*-glycero-3-phosphocholine—of sPLA_2_ [29]. These results suggest that Se-sPLA_2_ secreted from the midgut acts in lipid digestion by solubilizing dietary neutral lipids. While literature on the topic is rather thin, most invertebrates, including insects, do not produce bile salts [66] which in vertebrates solubilize neutral lipids for digestion. The sPLA_2_-mutant larvae might have suffered from poor lipid digestion, resulting in poorly developed pupae.

sPLA_2_-mutagenesis also led to severe fecundity impairment. Among eicosanoids, PGs particularly mediate chorion production in *S*. *exigua* along with other insects such as *B*. *mori* and *D*. *melanogaster* by coordinating the expression of separate chorion genes [13, 67, 68]. During vitellogenesis, nurse cells degenerate by delivering their cytoplasm to the near-by oocyte [69] in a process called nurse cell dumping, which is also driven by cytoskeletal rearrangement. In particular, the protein fascin, which is associated with actin filament-bundling, is activated by PGE_2_ in *S*. *exigua* [70]. Thus, a lack of sPLA_2_ activity in the deletion mutant would fail to produce enough PGE_2_ and lead to poor oocyte development. This may result in a significant decrease in the egg hatch rate in mutant progeny.

Altogether, the results of this study indicate that over 69% of the expressed genes in *S*. *exigua* larvae are under the control of eicosanoids. Further, about 36% of the immune-associated genes are controlled by the eicosanoids in *S*. *exigua*.

## Supporting information

S1 Fig Immune genes associated with *sPLA*_*2*_ expression. (A) AMPs. (B) Serpins. (C) Phenoloxidase (PO). (D) Toll immune signal components. (E) Pattern recognition receptors. (F) Immune mediators. (G) Actin polymerization factors. (H) IMD immune signal components.
(DOCX)

S1 Table Primers used in this study. (DOCX)

S2 Table Summary of RNA-seq analysis. (DOCX)

S3 Table Assembly statistics of clustered contigs. (DOCX)

S4 Table Immune-associated genes of *S*. *exigua*. The genes with at least 30 times fold change (up or down) were selected from 6,150 DEGs in G1 of Fig 4B.
(DOCX)

S5 Table Genes associated with sPLA_2_ in *S*. *exigua*. The genes with at least 50 times fold change (up or down) were selected from 8,944 DEGs in G2 of Fig 4B.
(DOCX)

S6 Table Immune genes associated with sPLA_2_ in *S*. *exigua*. The genes with at least ten times fold change (up or down) were selected from 2,216 DEGs common in both G1 and G2 in Fig 4B.
(DOCX)

S1 Raw images (PDF)

S2 Raw images (PDF)
