# Supplementary figures and images for: A PLA2 deletion mutant using CRISPR/Cas9 coupled to RNASeq reveals insect immune genes associated with eicosanoid signaling
Source: PLoS One. 2024 Jul 17;19(7):e0304958. doi: 10.1371/journal.pone.0304958 (PMC11253937; doi:10.1371/journal.pone.0304958)

**(A)**


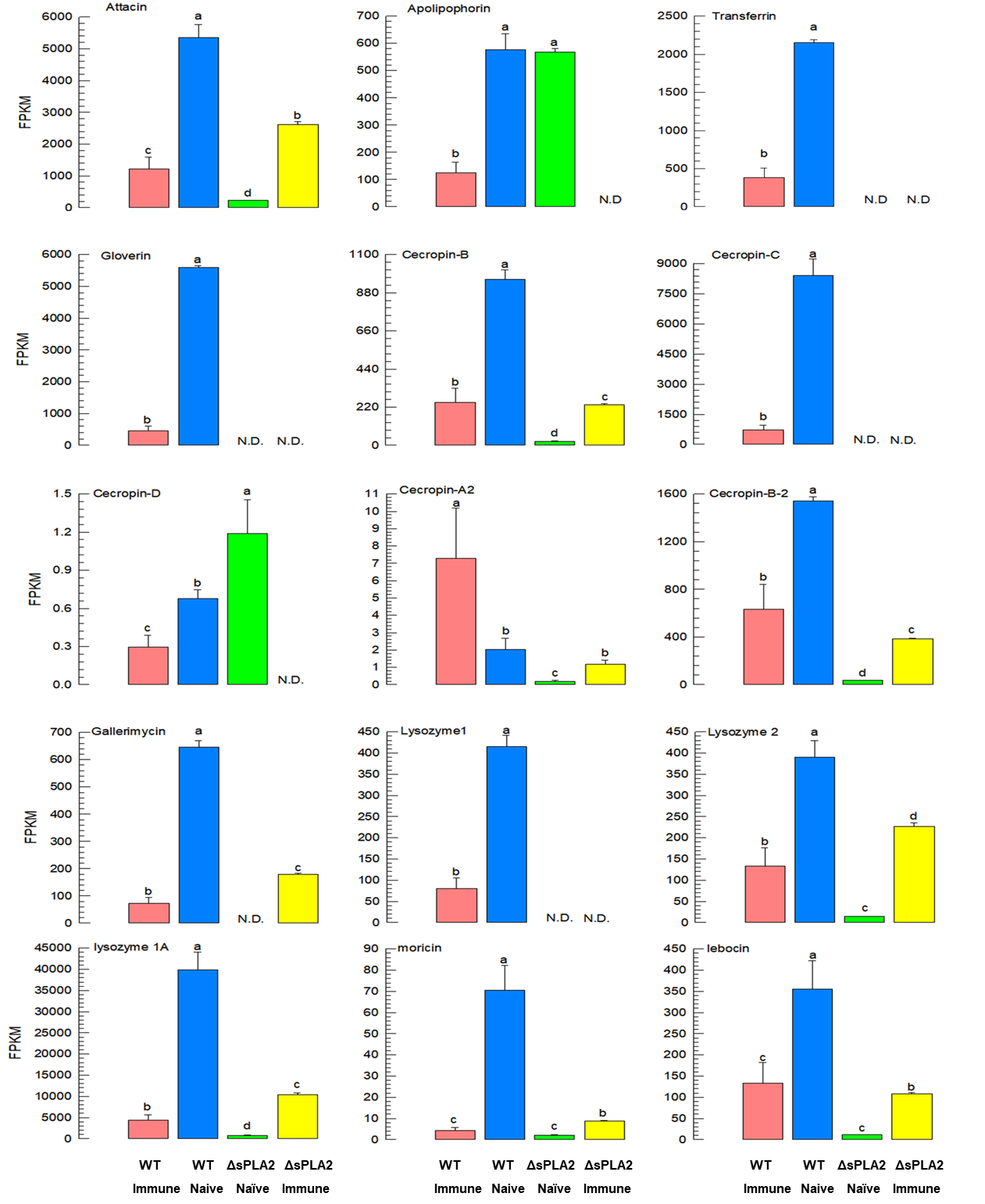


**(B)**

**
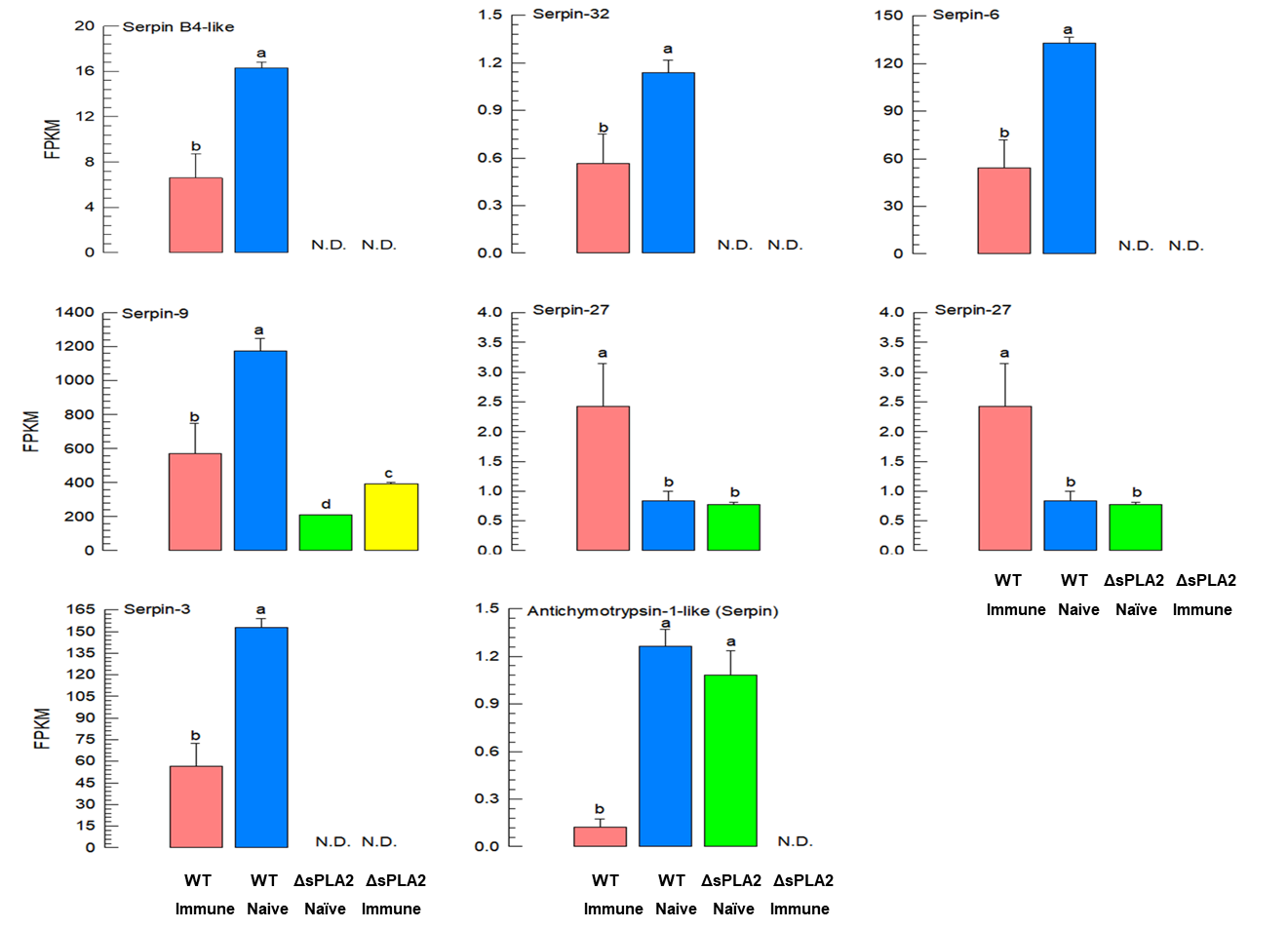
**

**(C)**


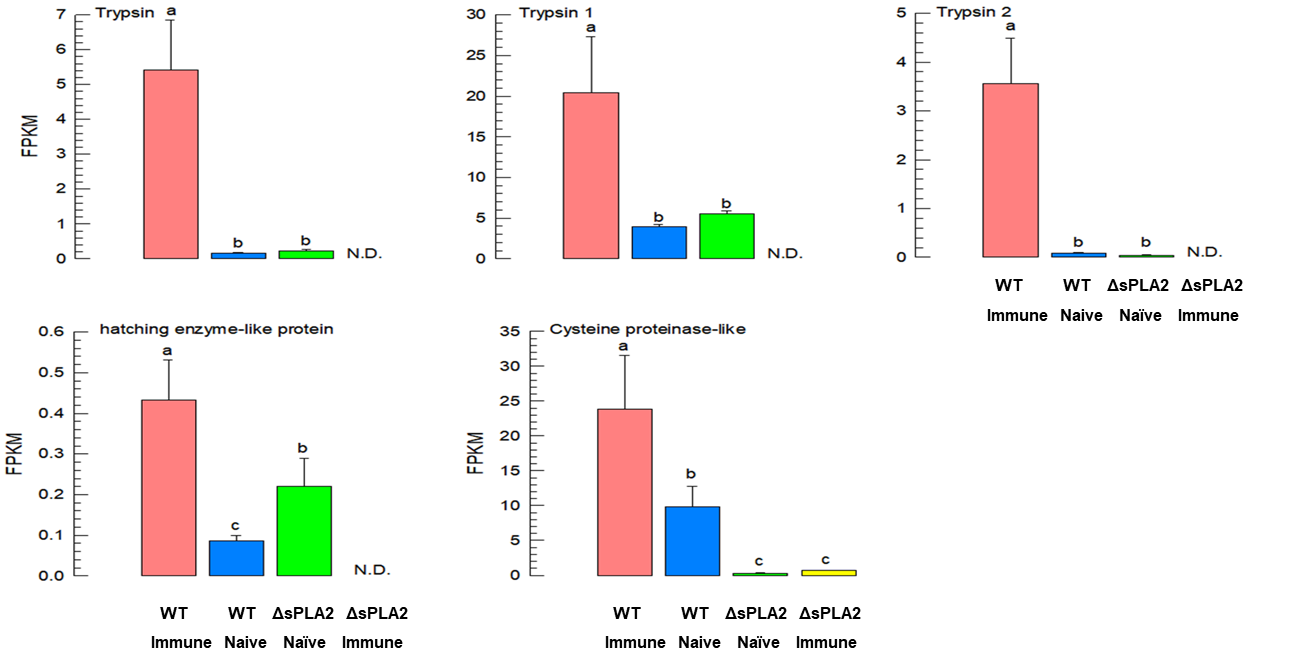


**(D)**

**
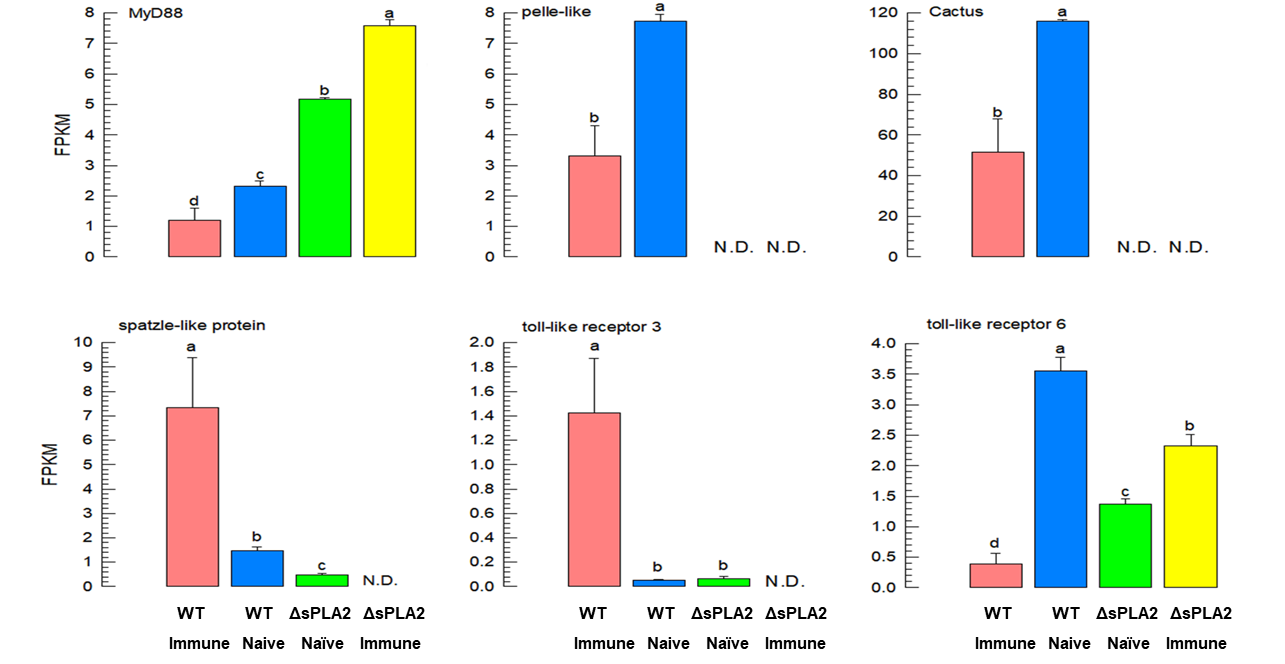
**

**(E)**  **
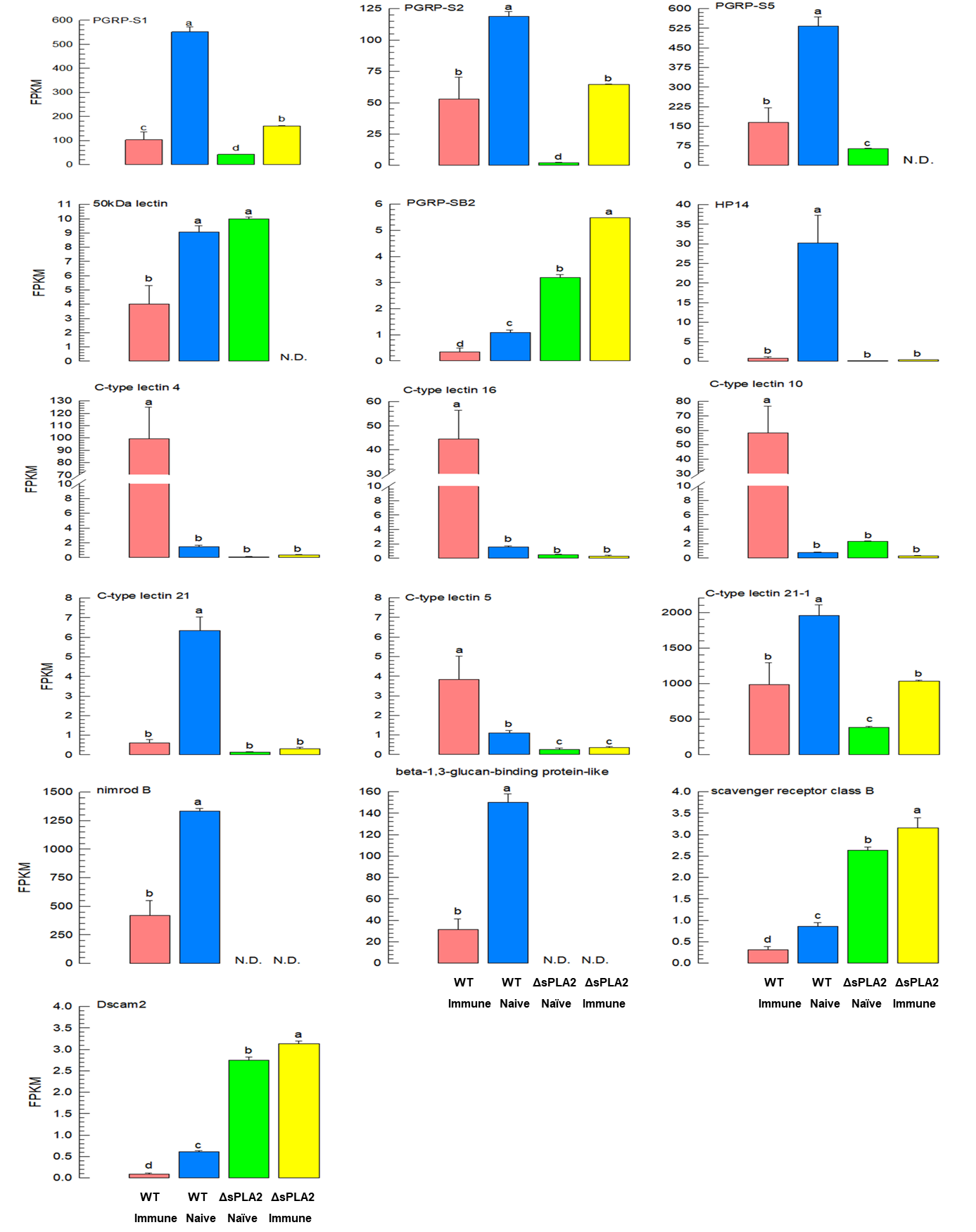
**

**(F)**


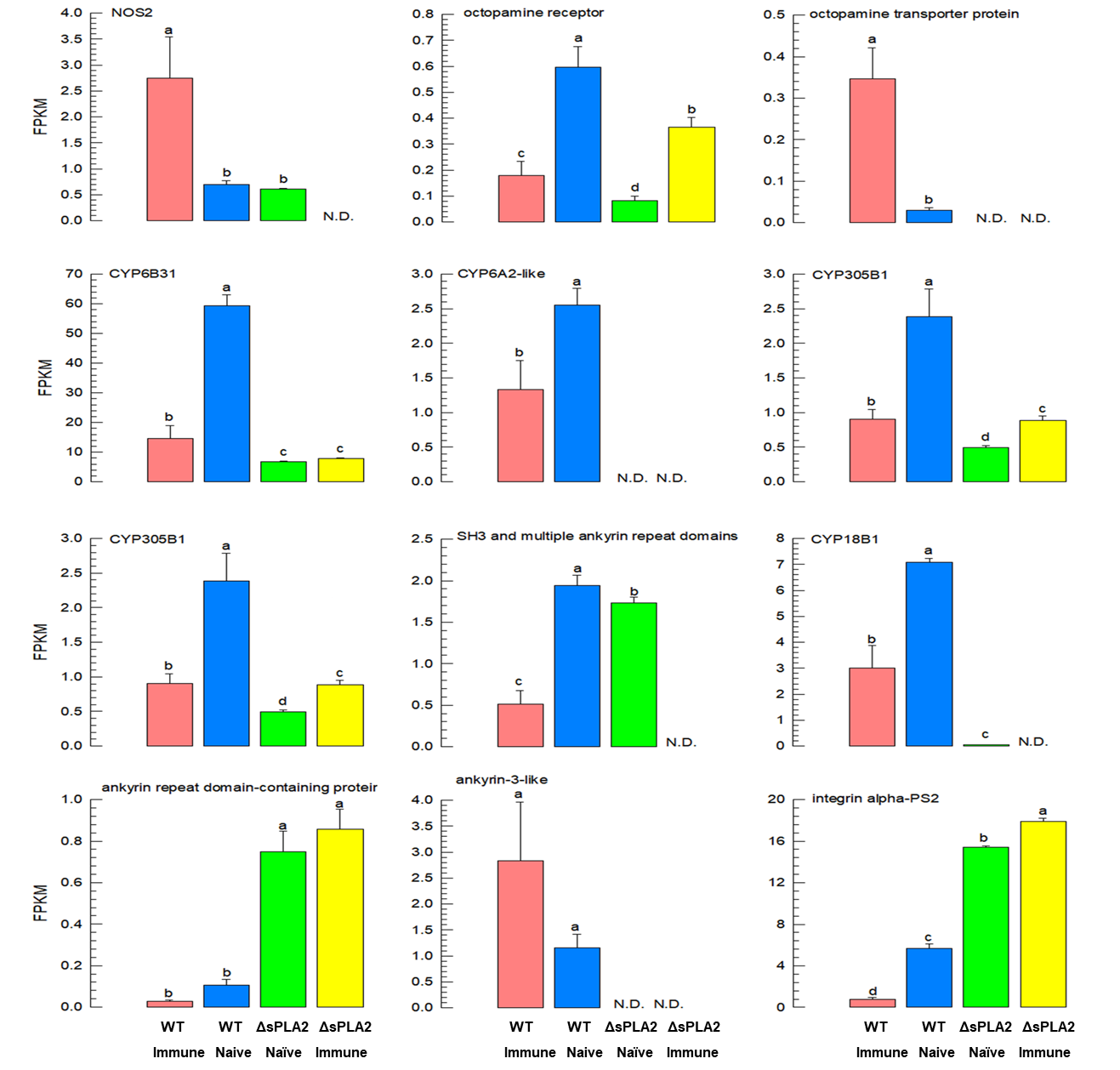


**(G)**


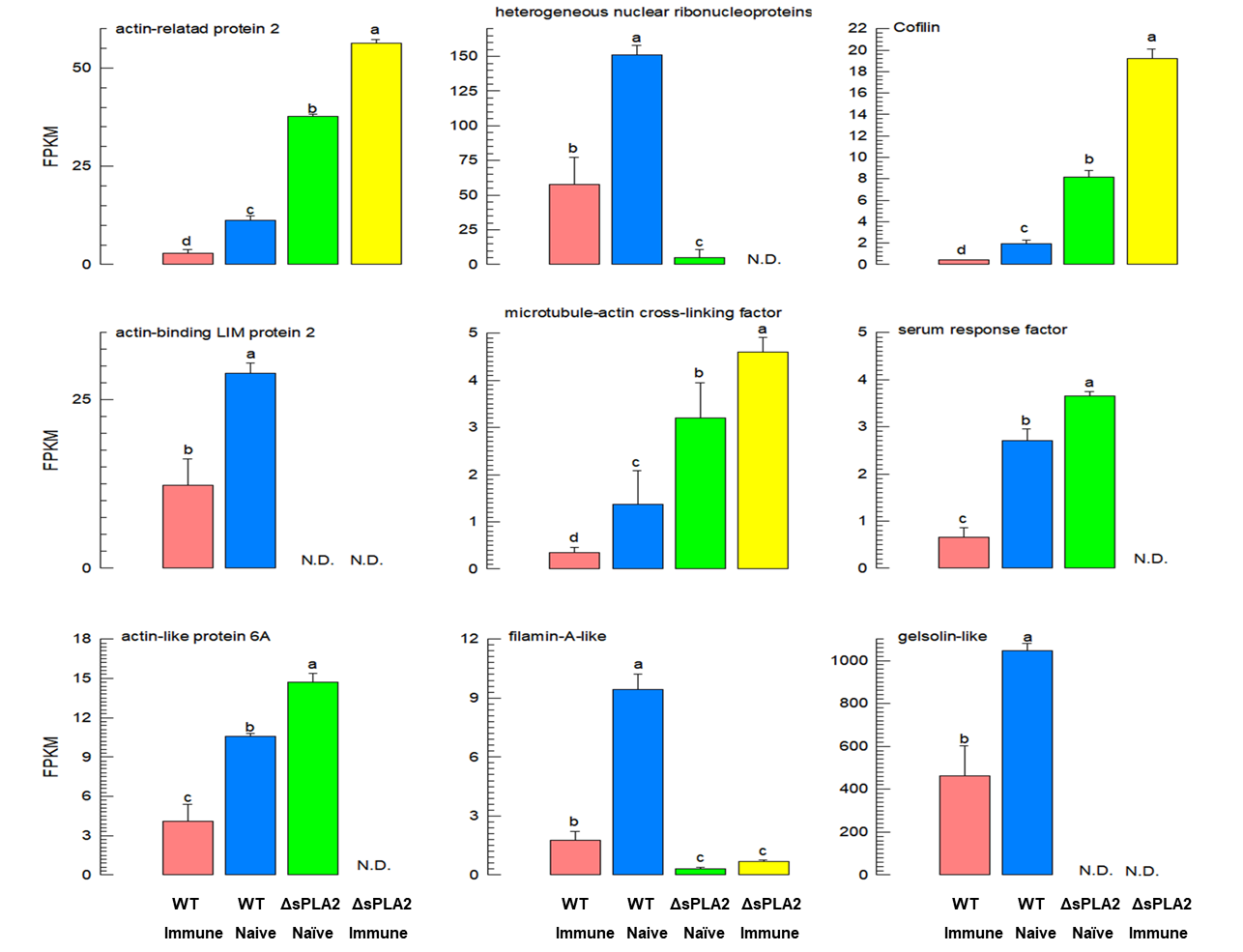


**(H)**


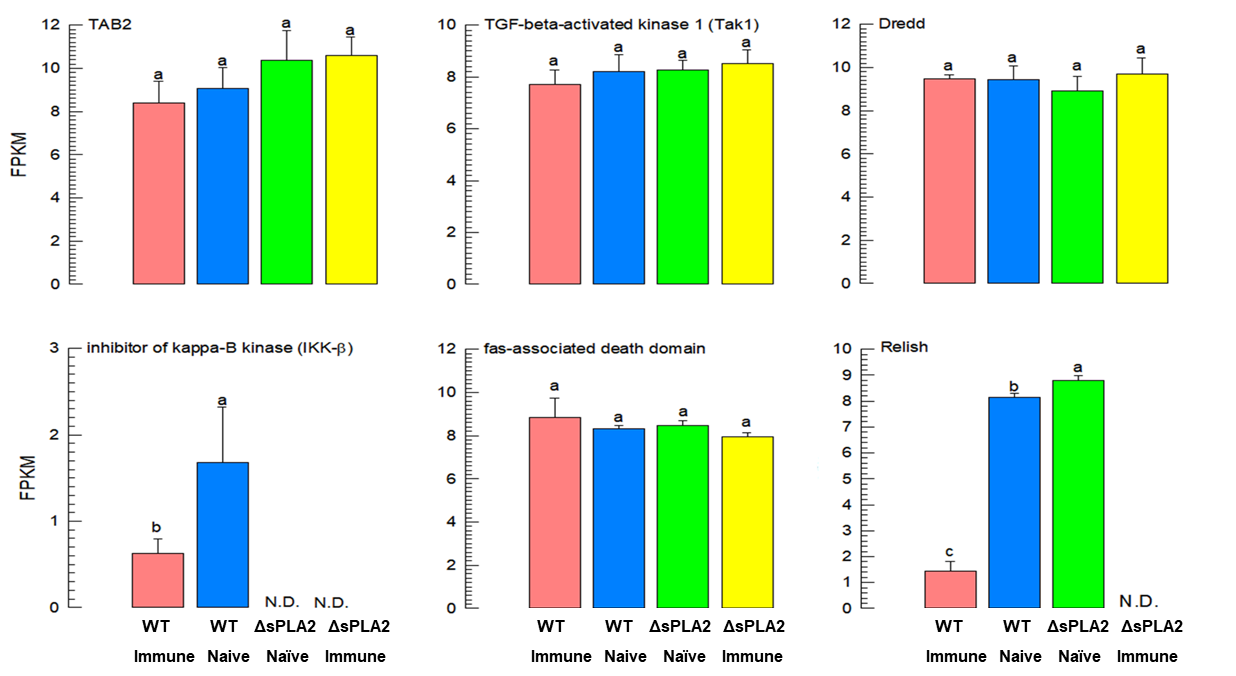

Supplement: S1 Fig — (A) AMPs. (B) Serpins. (C) Phenoloxidase (PO). (D) Toll immune signal components. (E) Pattern recognition receptors. (F) Immune mediators. (G) Actin polymerization factors. (H) IMD immune signal components. (DOCX) [file pone.0304958.s001.docx]

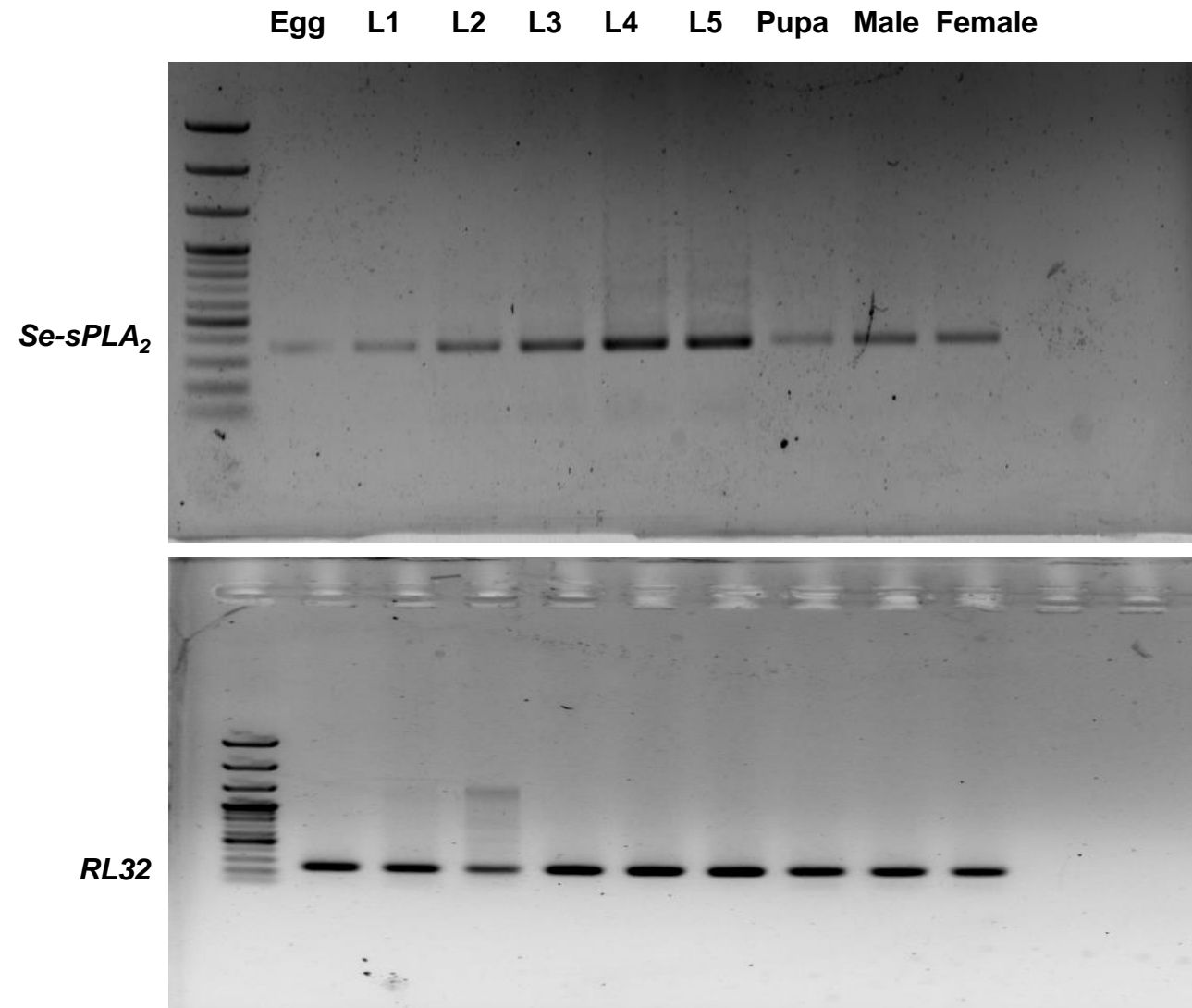

Figure 1A

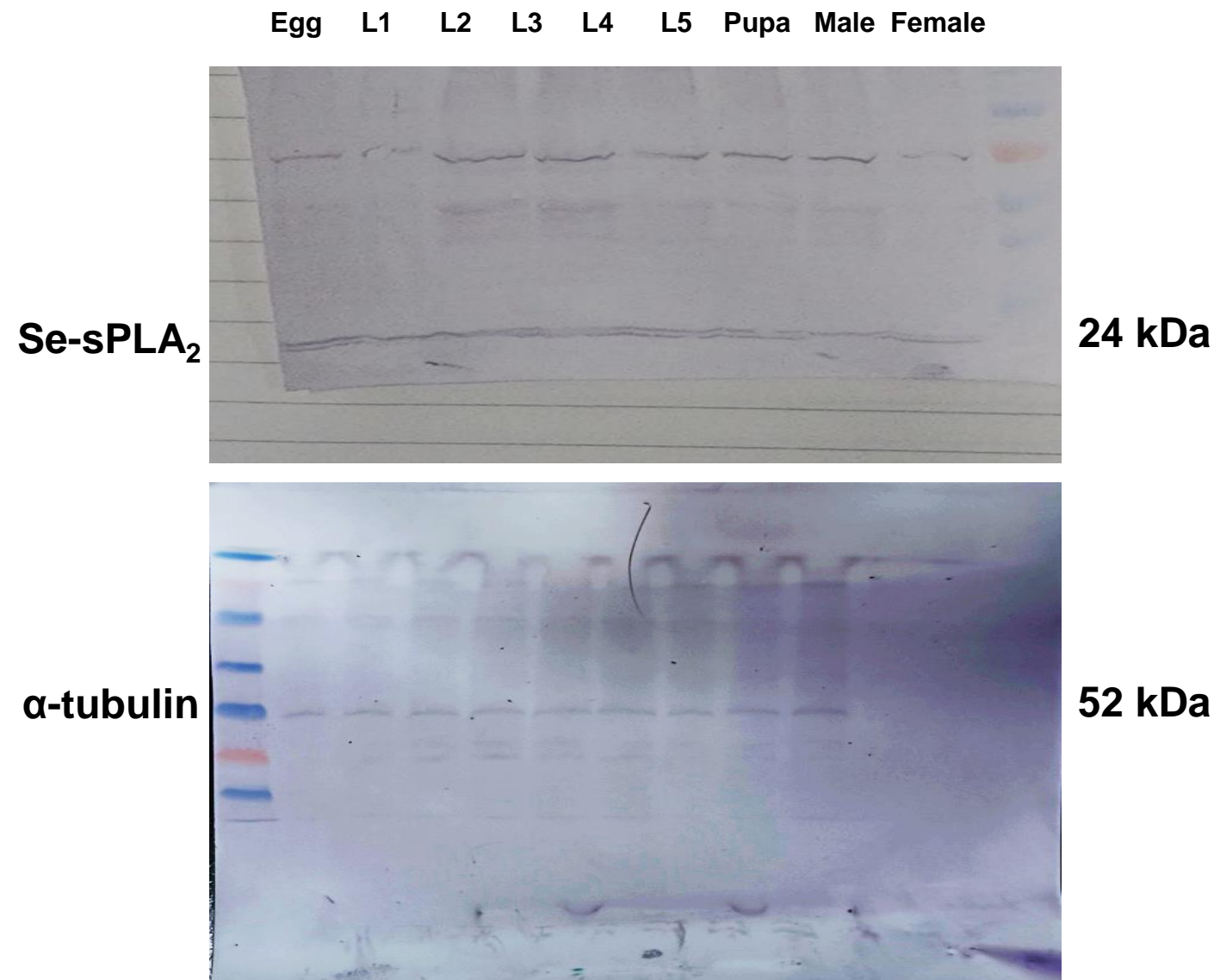

Figure 1B

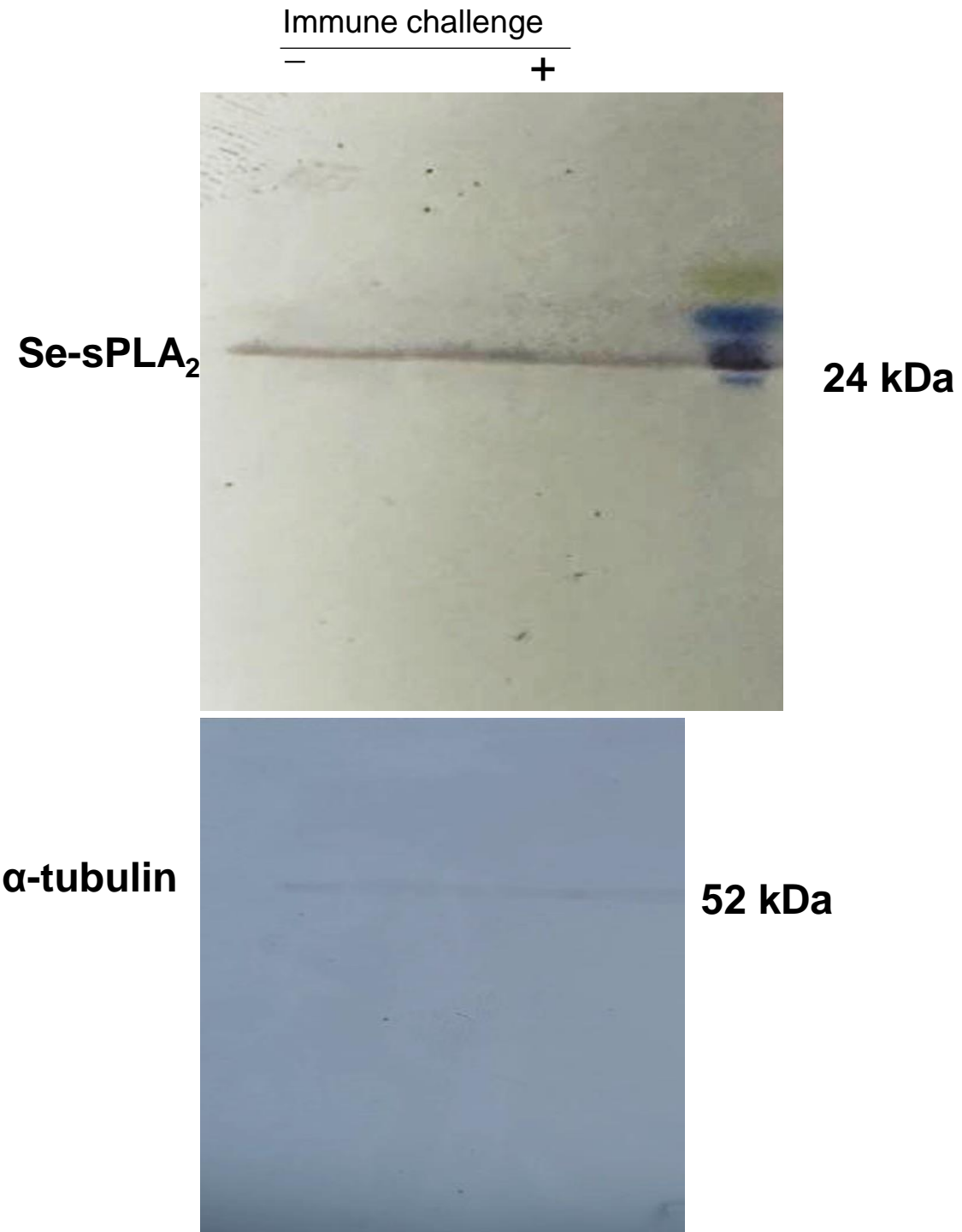

Figure 1C

Supplement: S2 Raw images — (PDF) [file pone.0304958.s009.pdf]
