# Supplementary material for: A PLA2 deletion mutant using CRISPR/Cas9 coupled to RNASeq reveals insect immune genes associated with eicosanoid signaling
Source: PLoS One. 2024 Jul 17;19(7):e0304958. doi: 10.1371/journal.pone.0304958 (PMC11253937; doi:10.1371/journal.pone.0304958)
Supplement: S1 Table — (DOCX) [file pone.0304958.s002.docx]

**S1 Table. Primers used in this study**

| **Primer** | **Use** | **Orientation** | **Sequence (5ʹ - 3ʹ)** | **Annealing temperature (°C)** | **Amplicon**  **(bp)** |
| --- | --- | --- | --- | --- | --- |
| ΔsPLA_2_ | Mutant  diagnosis | Forward  Reverse | CGGCAGCGAGTACCTTATT  GATTGAGTTGTGCTTGTGATGAG | 57 | 230 |
| RL32 | qPCR | Forward  Reverse | ATGCCCAACATTGGTTACGG  TTCGTTCTCCTGGCTGCGGA | 52 | 270 |
| Apolipophorin III | qPCR | Forward  Reverse | AGTGTCGCCAAGTTGTTCGTG  CTCCTGCGCGGTGTTCTGCA | 52 | 420 |
| Attacin1 | qPCR | Forward  Reverse | GCTTTCCTCTCCAGGAATATG  CCTTAGAGTAAATCCAGTGG | 52 | 276 |
| Attacin2 | qPCR | Forward  Reverse | TCCCGAATGTGCCCAACTTC  GAAAGATCTGCCGAAAGTAAG | 52 | 254 |
| Defensin | qPCR | Forward  Reverse | ATGGGTGTTAAGGTAATAAATGTG  GCAACTACATGTATGACTAACGC | 52 | 303 |
| Gallerimycin | qPCR | Forward  Reverse | TCAGTCATGAAAGCTTGCGTA  TCGCACACATTGGCATCCATTC | 52 | 222 |
| Hemolin | qPCR | Forward  Reverse | AAGACCAGGGCGAGTACAAG  AGCGACATGAACCAAGGTTTC | 52 | 347 |
| Transferrin1 | qPCR | Forward  Reverse | GTCCCTCTCTGTCCTGAAGG  CAGAAACACGAAGAAAGATGG | 52 | 370 |
| Transferrin2 | qPCR | Forward  Reverse | GATGTTCTGGCGCAGCTGTC  CCGGCTGAACGCAAACACAG | 52 | 288 |
