# Supplementary material for: A PLA2 deletion mutant using CRISPR/Cas9 coupled to RNASeq reveals insect immune genes associated with eicosanoid signaling
Source: PLoS One. 2024 Jul 17;19(7):e0304958. doi: 10.1371/journal.pone.0304958 (PMC11253937; doi:10.1371/journal.pone.0304958)
Supplement: S2 Table — (DOCX) [file pone.0304958.s003.docx]

**S2 Table. Summary of RNASeq analysis**

| Treatment | Total raw read bases (bp) | Total trimmed  read bases (bp) | # of processed reads | # of mapped reads |
| --- | --- | --- | --- | --- |
| ΔsPLA_2_-naïve-1 | 11,102,457,724 | 10,905,019,920 | 108,522,932 | 94,254,584 (86.85%) |
| ΔsPLA_2_-naïve-2 | 13,537,947,080 | 13,317,843,143 | 132,546,840 | 115,925,388 (87.46%) |
| ΔsPLA_2_-naïve-3 | 13,432,106,958 | 13,230,770,315 | 131,633,522 | 115,068,646 (87.42%) |
| ΔsPLA_2_-immune-1 | 11,266,768,766 | 11,079,182,052 | 110,238,148 | 90,549,710 (82.14%) |
| ΔsPLA_2_-immune-2 | 13,197,074,302 | 13,001,392,662 | 129,345,692 | 106,165,022 (82.08%) |
| ΔsPLA_2_-immune-3 | 14,722,893,624 | 14,482,033,600 | 144,164,570 | 118,587,008 (82.26%) |
| WT-naïve-1 | 11,030,090,214 | 10,829,628,831 | 107,995,714 | 80,888,334 (74.9%) |
| WT-naïve-2 | 10,571,785,746 | 10,419,223,975 | 103,597,880 | 79,537,530 (76.78%) |
| WT-naïve-3 | 11,353,029,028 | 11,129,109,048 | 110,862,812 | 96,906,268 (87.41%) |
| WT-immune-1 | 12,676,655,844 | 12,494,384,968 | 124,393,856 | 93,666,738 (75.3%) |
| WT-immune-2 | 10,933,765,908 | 10,757,170,374 | 107,037,980 | 79,848,574 (74.6%) |
| WT-immune-3 | 11,672,324,570 | 11,469,825,089 | 114,410,422 | 86,075,276 (75.23%) |
