# Supplementary material for: A PLA2 deletion mutant using CRISPR/Cas9 coupled to RNASeq reveals insect immune genes associated with eicosanoid signaling
Source: PLoS One. 2024 Jul 17;19(7):e0304958. doi: 10.1371/journal.pone.0304958 (PMC11253937; doi:10.1371/journal.pone.0304958)
Supplement: S3 Table — (DOCX) [file pone.0304958.s004.docx]

**S3 Table**. **Assembly statistics of clustered contigs**

| Assembly | Number of  contigs | Largest  contig (bp) | Number of  unigenes |
| --- | --- | --- | --- |
| Total trinity 'genes' | 193,225 | 193,225 | 174,839 |
| GC percent | 39.91 | 39,16 | 39.04 |
| N50 | 984 | 600 | 651 |
| Maximum contig length | 27,920 | 27,920 | 27,920 |
| Average contig length | 611.08 | 511.81 | 528.83 |
| Total assembled bases | 137,323,928 | 98,893,599 | 92,460,674 |
