# Supplementary material for: A PLA2 deletion mutant using CRISPR/Cas9 coupled to RNASeq reveals insect immune genes associated with eicosanoid signaling
Source: PLoS One. 2024 Jul 17;19(7):e0304958. doi: 10.1371/journal.pone.0304958 (PMC11253937; doi:10.1371/journal.pone.0304958)
Supplement: S4 Table — The genes with at least 30 times fold change (up or down) were selected from 6,150 DEGs in G1 of Fig 4B. (DOCX) [file pone.0304958.s005.docx]

**S4 Table**. **Immune-associated genes of *S. exigua*.** The genes with at least 30 times fold change (up or down) were selected from 6,150 DEGs in G1 of Fig 4B.

| **Contig** | **Gene** | **Gene bank accession number** | **Fold change** | **FPKM** | |
| --- | --- | --- | --- | --- | --- |
|  |  |  |  | **WT+EC** | **WT** |
| c90624_g7_i1 | Hypothetical protein | CCQ19275.1 | 538.61 | 3.96 ± 1.07 | 1990.69 ± 218.31 |
| c90413_g4_i1 | Fibrillin-3-like isoform X2 | XP_022837034.1 | 292.17 | 0.13 ± 0.05 | 34.10 ± 5.07 |
| c89066_g1_i1 | Hemolin-like | XP_022819117.1 | 103.67 | 44.45 ± 14.02 | 4234.66 ± 280.31 |
| c90413_g3_i1 | Fibrillin-2-like isoform X4 | XP_022837036.1 | 85.19 | 1.58 ± 0.56 | 120.08 ± 6.16 |
| c85021_g1_i1 | Uncharacterized protein | XP_022835871.1 | 81.68 | 85.69 ± 28.14 | 6287.15 ± 552.43 |
| c85122_g5_i1 | Inducible metalloproteinase inhibitor protein-like | XP_022816597.1 | 80.37 | 0.89 ± 0.44 | 62.23 ± 18.50 |
| c85356_g1_i1 | Uncharacterized protein | XP_022835765.1 | 67.84 | 30.81 ± 10.09 | 1906.59 ± 317.86 |
| c193741_g1_i1 | Acanthoscurrin-1-like | XP_022835230.1 | 52.77 | 0.28 ± 0.17 | 13.32 ± 2.60 |
| c62241_g1_i1 | Low-density lipoprotein receptor-related protein 4 | XP_022829670.1 | 49.34 | 0.02 ± 0.01 | 1.30 ± 0.25 |
| c89830_g1_i4 | Uncharacterized protein | XP_022818011.1 | 47.07 | 0.09 ± 0.04 | 4.03 ± 0.15 |
| c54451_g1_i2 | Lipase member H-like | XP_022821336.1 | 46.21 | 0.39 ± 0.16 | 16.42 ± 4.31 |
| c89700_g1_i1 | Venom carboxylesterase-6-like isoform X2 | XP_022837633.1 | 45.93 | 3.63 ± 1.14 | 151.25 ± 15.95 |
| c88494_g1_i1 | Antennal esterase CXE5 | ADR64702.1 | 45.36 | 1.65 ± 0.50 | 68.99 ± 3.63 |
| c89009_g1_i1 | Moricin-like peptide D | ABQ42580.1 | 40.75 | 0.24 ± 0.10 | 8.28 ± 1.30 |
| c82096_g1_i1 | Modular serine protease-like | XP_022834261.1 | 37.57 | 0.86 ± 0.29 | 30.25 ± 12.13 |
| c88004_g1_i4 | Hypothetical protein | PCG77460.1 | 37.38 | 0.52 ± 0.18 | 17.13 ± 2.31 |
| c90648_g3_i1 | Uncharacterized protein | XP_022817924.1 | 36.30 | 42.54 ± 14.35 | 1,341.92 ± 4.06 |
| c67381_g1_i2 | Myosin heavy chain, muscle isoform X16 | XP_021194473.1 | 33.94 | 0.04 ± 0.00 | 1.37 ± 0.19 |
| c82894_g2_i1 | Uncharacterized protein | XP_022816656.1 | -30.06 | 22.81 ± 6.89 | 0.67 ± 0.22 |
| c79167_g1_i1 | Uncharacterized protein | XP_022828774.1 | -30.71 | 6.64 ± 1.48 | 0.22 ± 0.11 |
| c86307_g1_i2 | Uncharacterized protein | XP_022827715.1 | -31.91 | 1.61 ± 0.45 | 0.04 ± 0.02 |
| c87843_g4_i1 | Endocuticle structural glycoprotein sgabd-8-like | XP_022816372.1 | -32.32 | 74.87 ± 20.51 | 2.22 ± 0.24 |
| c131456_g1_i1 | Uncharacterized protein | XP_022824836.1 | -32.37 | 56.43 ± 14.12 | 1.76 ± 0.43 |
| c84776_g2_i1 | Uncharacterized protein | XP_022834170.1 | -32.58 | 23.56 ± 6.95 | 0.64 ± 0.30 |
| c84458_g2_i1 | Lipase member H-like | XP_022822384.1 | -33.15 | 11.15 ± 2.66 | 0.33 ± 0.02 |
| c79986_g1_i1 | Protein obstructor-E-like | XP_022824511.1 | -34.50 | 73.16 ± 20.03 | 2.06 ± 0.46 |
| c81691_g1_i1 | Uncharacterized protein | XP_022822010.1 | -34.58 | 14.01 ± 3.38 | 0.41 ± 0.19 |
| c82990_g1_i1 | Repat21 | AFH57141.1 | -35.25 | 55.64 ± 18.15 | 1.44 ± 0.50 |
| c79170_g2_i1 | Trypsin, alkaline C-like | XP_022821660.1 | -36.05 | 12.11 ± 3.48 | 0.31 ± 0.03 |
| c78282_g2_i1 | Pancreatic triacylglycerol lipase-like | XP_022822284.1 | -36.09 | 10.91 ± 3.34 | 0.28 ± 0.04 |
| c84381_g1_i4 | Trypsin-like isoform X1 | XP_022825984.1 | -36.15 | 5.42 ± 1.44 | 0.15 ± 0.03 |
| c88041_g1_i1 | Putative serine protease | XP_022828698.1 | -36.20 | 6.84 ± 1.93 | 0.19 ± 0.13 |
| c85804_g1_i2 | Serine proteinase stubble isoform X4 | XP_022817272.1 | -38.02 | 1.09 ± 0.25 | 0.03 ± 0.01 |
| c82894_g1_i1 | Uncharacterized protein | XP_022815540.1 | -38.14 | 29.38 ± 8.25 | 0.75 ± 0.31 |
| c85775_g1_i1 | Protein split ends-like | XP_022837898.1 | -39.12 | 4.63 ± 1.15 | 0.12 ± 0.05 |
| c131946_g1_i1 | Uncharacterized protein | XP_022825069.1 | -39.33 | 17.15 ± 5.20 | 0.41 ± 0.17 |
| c81847_g1_i1 | Trypsin, alkaline C-like | XP_022836703.1 | -39.48 | 10.45 ± 2.81 | 0.25 ± 0.12 |
| c84818_g1_i1 | Serine proteinase stubble | XP_022817110.1 | -40.11 | 3.55 ± 0.93 | 0.08 ± 0.02 |
| c89004_g3_i1 | Uncharacterized protein | XP_022835049.1 | -41.45 | 3.32 ± 0.89 | 0.07 ± 0.02 |
| c59988_g1_i1 | Cuticle protein-like | XP_022824787.1 | -41.52 | 10.99 ± 2.81 | 0.25 ± 0.11 |
| c135224_g1_i1 | Cuticle protein 3-like | XP_022816339.1 | -41.99 | 33.16 ± 9.24 | 0.76 ± 0.13 |
| c89704_g1_i2 | Collagenase-like | XP_022831731.1 | -42.97 | 2.61 ± 0.76 | 0.05 ± 0.02 |
| c69275_g1_i1 | Lactase-phlorizin hydrolase-like | XP_022818655.1 | -43.25 | 4.65 ± 1.26 | 0.09 ± 0.07 |
| c53001_g1_i1 | Uncharacterized protein | XP_022828639.1 | -43.55 | 4.42 ± 1.29 | 0.10 ± 0.08 |
| c92954_g1_i1 | Synaptonemal complex protein 4 isoform X2 | XP_022835391.1 | -43.83 | 55.63 ± 14.86 | 1.23 ± 0.04 |
| c82011_g1_i1 | Trypsin, alkaline C-like | XP_022815740.1 | -44.43 | 3847.54 ± 1179.61 | 82.19 ± 29.36 |
| c173389_g1_i1 | Juvenile hormone esterase-like | XP_022829393.1 | -44.62 | 19.14 ± 5.20 | 0.41 ± 0.10 |
| c87764_g2_i1 | Carboxypeptidase B-like | XP_022832131.1 | -44.99 | 14.27 ± 3.44 | 0.31 ± 0.13 |
| c112566_g1_i1 | Fatty acyl-coa reductase wat-like | XP_022837119.1 | -46.18 | 24.70 ± 6.90 | 0.52 ± 0.14 |
| c86370_g1_i1 | Uncharacterized protein | XP_022832710.1 | -46.88 | 30.00 ± 8.12 | 0.63 ± 0.09 |
| c112321_g1_i1 | Acyl-coa-binding protein-like | XP_022820273.1 | -47.68 | 2945.37 ± 881.88 | 57.34 ± 17.18 |
| c86316_g3_i1 | Protein app1-like | XP_022821523.1 | -48.48 | 16.20 ± 4.45 | 0.31 ± 0.05 |
| c69180_g1_i1 | Uncharacterized protein | XP_022815138.1 | -50.44 | 3.89 ± 1.18 | 0.07 ± 0.01 |
| c52284_g1_i2 | Uncharacterized protein | XP_022826927.1 | -51.02 | 54.84 ± 14.54 | 1.08 ± 0.45 |
| c90910_g2_i3 | Aminopeptidase N5 | AIK27005.1 | -51.32 | 7.51 ± 2.06 | 0.14 ± 0.07 |
| c77464_g1_i1 | Leukocyte surface antigen CD53-like | XP_022823982.1 | -51.69 | 39.13 ± 10.32 | 0.75 ± 0.20 |
| c86248_g1_i1 | Midgut class 1 aminopeptidase N | AAP44964.1 | -52.14 | 88.33 ± 25.00 | 1.68 ± 0.65 |
| c81090_g1_i1 | Fatty acid-binding protein 1-like | XP_022831666.1 | -52.23 | 602.94 ± 178.79 | 10.74 ± 2.04 |
| c88803_g3_i1 | Fatty acid-binding protein | XP_022817848.1 | -53.15 | 1.59 ± 0.40 | 0.03 ± 0.02 |
| c89022_g3_i1 | Glucose dehydrogenase [FAD, quinone]-like | XP_022815022.1 | -53.19 | 2.61 ± 0.73 | 0.05 ± 0.02 |
| c74898_g1_i1 | Uncharacterized protein | XP_022822755.1 | -54.19 | 23.65 ± 5.74 | 0.43 ± 0.22 |
| c72065_g1_i1 | Repat15 | AFH57135.1 | -56.59 | 50.73 ± 15.00 | 0.83 ± 0.18 |
| c16441_g1_i1 | Carboxypeptidase B-like | XP_022838055.1 | -58.57 | 6.88 ± 1.99 | 0.11 ± 0.07 |
| c89914_g1_i1 | Solute carrier family 22 member 13-like | XP_022815828.1 | -62.10 | 11.96 ± 3.66 | 0.18 ± 0.05 |
| c90136_g2_i1 | Uncharacterized protein | XP_022827272.1 | -62.21 | 5.29 ± 1.53 | 0.08 ± 0.03 |
| c82980_g1_i3 | Uncharacterized protein | XP_022837897.1 | -65.33 | 61.86 ± 16.61 | 0.92 ± 0.04 |
| c68272_g1_i1 | Uncharacterized protein | XP_022821214.1 | -66.43 | 99.15 ± 25.74 | 1.49 ± 0.27 |
| c94524_g1_i1 | Uncharacterized protein | XP_022825552.1 | -68.68 | 32.06 ± 8.32 | 0.46 ± 0.02 |
| c85486_g1_i1 | Bll4 | AKP99426.1 | -70.05 | 58.02 ± 18.74 | 0.74 ± 0.13 |
| c89146_g4_i2 | Uncharacterized protein | XP_022835587.1 | -70.56 | 14.76 ± 3.88 | 0.21 ± 0.13 |
| c82950_g1_i1 | Global transcription regulator sge1 | XP_022826910.1 | -71.23 | 3.54 ± 0.83 | 0.05 ± 0.01 |
| c87763_g1_i1 | Midgut class 2 aminopeptidase N | AAP44965.1 | -74.22 | 31.17 ± 8.99 | 0.41 ± 0.18 |
| c87369_g3_i1 | Calphotin-like | XP_022813970.1 | -74.76 | 2122.99 ± 647.18 | 26.89 ± 10.20 |
| c49886_g1_i1 | Laccase-1 | XP_022819652.1 | -74.89 | 7.21 ± 1.96 | 0.09 ± 0.04 |
| c15325_g1_i1 | Hypothetical protein | PCG70881.1 | -80.39 | 7.21 ± 1.94 | 0.08 ± 0.08 |
| c73815_g2_i1 | Glycine-rich protein DOT1-like | XP_022819775.1 | -87.01 | 556.99 ± 146.71 | 6.20 ± 0.69 |
| c80865_g1_i1 | Uncharacterized protein | XP_022820807.1 | -88.00 | 5.42 ± 1.36 | 0.06 ± 0.02 |
| c86001_g1_i1 | Fatty acid synthase-like | XP_022831847.1 | -88.65 | 11.24 ± 2.95 | 0.12 ± 0.04 |
| c40113_g1_i1 | Skin secretory protein xp2-like | XP_022825188.1 | -92.08 | 31.22 ± 8.24 | 0.33 ± 0.03 |
| c85076_g1_i1 | Uncharacterized protein | XP_022821894.1 | -93.28 | 13.35 ± 3.91 | 0.14 ± 0.06 |
| c83086_g1_i1 | Aminopeptidase N | AAT99437.1 | -93.50 | 49.99 ± 14.01 | 0.52 ± 0.16 |
| c92528_g1_i1 | Proline-rich protein 4-like | XP_022825189.1 | -95.19 | 110.07 ± 30.44 | 1.13 ± 0.20 |
| c88143_g2_i3 | Membrane-bound alkaline phosphatase-like | XP_022822920.1 | -97.70 | 14.40 ± 4.23 | 0.13 ± 0.11 |
| c76712_g1_i1 | Luciferin 4-monooxygenase-like | XP_022816435.1 | -98.70 | 5.41 ± 1.46 | 0.05 ± 0.00 |
| c84724_g1_i1 | Aminoacylase-1A-like | XP_022815042.1 | -101.15 | 39.88 ± 12.50 | 0.36 ± 0.16 |
| c81006_g1_i1 | Repat16 | AFH57136.1 | -102.30 | 27.39 ± 7.46 | 0.26 ± 0.14 |
| c12854_g2_i1 | Pollen-specific leucine-rich repeat extensin-like protein 4 | XP_022820303.1 | -103.53 | 63.96 ± 16.31 | 0.59 ± 0.12 |
| c86041_g1_i1 | Phospholipase A1-like | XP_022814428.1 | -107.72 | 13.12 ± 4.82 | 0.10 ± 0.03 |
| c25686_g1_i1 | Lipase 3-like | XP_022830435.1 | -109.95 | 8.55 ± 2.81 | 0.07 ± 0.03 |
| c87574_g1_i3 | Histone acetyltransferase p300-like | XP_022826940.1 | -113.09 | 24.92 ± 6.76 | 0.21 ± 0.07 |
| c70109_g2_i1 | Collagenase-like | XP_022826639.1 | -113.20 | 17.65 ± 4.64 | 0.15 ± 0.02 |
| c72186_g2_i2 | Tetra-peptide repeat homeobox protein 1-like | XP_022826659.1 | -115.51 | 3.11 ± 0.89 | 0.02 ± 0.01 |
| c12887_g1_i1 | Fatty acid-binding protein 1-like | XP_022831716.1 | -116.02 | 266.33 ± 73.54 | 2.20 ± 0.47 |
| c90789_g1_i1 | Peroxidase | XP_022826162.1 | -117.74 | 5.61 ± 1.41 | 0.04 ± 0.01 |
| c80007_g3_i2 | Trypsin, alkaline C-like | XP_022821670.1 | -120.12 | 252.55 ± 64.67 | 2.02 ± 0.50 |
| c28288_g1_i1 | Trypsin-like serine protease precursor | AIR09767.1 | -122.98 | 25.42± 6.68 | 0.19 ± 0.07 |
| c83732_g1_i1 | Protein split ends-like | XP_022837898.1 | -123.54 | 33.38 ± 9.03 | 0.26 ± 0.00 |
| c72952_g1_i1 | Uncharacterized protein | XP_022820339.1 | -123.88 | 43.31 ± 11.55 | 0.34 ± 0.04 |
| c85815_g1_i2 | Probable nuclear hormone receptor HR3 isoform X6 | XP_022816977.1 | -129.73 | 7.81 ± 1.84 | 0.06 ± 0.01 |
| c84177_g1_i1 | PREDICTED: mucin-2-like | XP_013195491.1 | -130.00 | 465.24 ± 145.58 | 3.36 ± 1.19 |
| c83677_g1_i1 | Ejaculatory bulb-specific protein 3-like | XP_021190501.1 | -132.06 | 26.90 ± 6.91 | 0.20 ± 0.12 |
| c85598_g1_i1 | Pancreatic triacylglycerol lipase-like | XP_022835499.1 | -133.01 | 8.31 ± 2.55 | 0.05 ± 0.02 |
| c82205_g2_i1 | Uncharacterized protein | XP_022837598.1 | -136.03 | 69.86 ± 18.59 | 0.51 ± 0.09 |
| c88652_g1_i1 | Polycalin | ANC90402.1 | -157.93 | 253.96 ± 67.03 | 1.61 ± 0.35 |
| c132630_g1_i1 | RNA-binding protein 33-like | XP_022829656.1 | -159.67 | 18.07 ± 4.79 | 0.11 ± 0.03 |
| c131481_g1_i1 | Uncharacterized protein | XP_022821909.1 | -164.90 | 12.65 ± 3.64 | 0.07 ± 0.05 |
| c190293_g1_i1 | Pro-resilin-like | XP_022826991.1 | -170.99 | 81.65 ± 22.35 | 0.47 ± 0.08 |
| c92153_g1_i1 | Testis-specific gene A8 protein-like | XP_022830264.1 | -186.15 | 45.15 ± 12.87 | 0.23 ± 0.05 |
| c79269_g1_i1 | Uncharacterized protein | XP_022831879.1 | -186.90 | 39.99 ± 10.15 | 0.20 ± 0.17 |
| c171177_g1_i1 | Uncharacterized protein | XP_022829990.1 | -201.44 | 17.09 ± 4.44 | 0.08 ± 0.01 |
| c88270_g9_i1 | Trypsin, alkaline C-like | XP_022815733.1 | -204.68 | 1032.77 ± 295.01 | 4.96 ± 2.21 |
| c14273_g1_i1 | Pollen-specific leucine-rich repeat extensin-like protein 1 | XP_022828128.1 | -210.44 | 51.71 ± 13.72 | 0.24 ± 0.04 |
| c40199_g1_i1 | Transmembrane protease serine 9-like | XP_022827086.1 | -218.33 | 38.77 ± 10.58 | 0.18 ± 0.13 |
| c86173_g1_i1 | Fibroin heavy chain-like | XP_022821902.1 | -218.74 | 22.78 ± 5.53 | 0.10 ± 0.05 |
| c89257_g1_i1 | Organic cation transporter protein-like | XP_022828223.1 | -228.19 | 11.86 ± 3.42 | 0.05 ± 0.03 |
| c54184_g1_i1 | Fatty acid-binding protein 1-like | XP_022831663.1 | -251.37 | 912.63 ± 265.76 | 3.42 ± 0.56 |
| c85919_g1_i1 | Uncharacterized family 31 glucosidase KIAA1161-like | XP_022824726.1 | -254.88 | 111.51 ± 31.57 | 0.42 ± 0.07 |
| c88495_g1_i1 | Arylsulfatase J-like | XP_022825369.1 | -258.71 | 12.08 ± 3.83 | 0.04 ± 0.00 |
| c86554_g1_i1 | Midgut class 4 aminopeptidase N | AAP44967.1 | -262.44 | 50.47 ± 13.94 | 0.18 ± 0.02 |
| c85147_g1_i1 | Bile salt-activated lipase-like | XP_022822371.1 | -262.68 | 15.61 ± 4.77 | 0.05 ± 0.02 |
| c87780_g1_i1 | Cuticle protein 8-like | XP_022815933.1 | -268.51 | 14.94 ± 4.08 | 0.05 ± 0.00 |
| c86311_g1_i1 | Acyl-coa Delta(11) desaturase-like | XP_022824753.1 | -288.29 | 57.59 ± 15.55 | 0.19 ± 0.05 |
| c71893_g1_i1 | Trypsin II-P29-like | XP_022824767.1 | -288.45 | 42.47 ± 10.66 | 0.14 ± 0.03 |
| c84179_g1_i1 | Uncharacterized protein | XP_022817989.1 | -301.49 | 61.40 ± 18.37 | 0.19 ± 0.11 |
| c80458_g1_i1 | Uncharacterized protein | XP_022817958.1 | -375.70 | 357.61 ± 104.43 | 0.91 ± 0.26 |
| c80759_g1_i2 | Titin | XP_022837584.1 | -390.32 | 142.54 ± 38.60 | 0.35 ± 0.04 |
| c74000_g1_i1 | Uncharacterized protein | XP_022827721.1 | -410.23 | 143.90 ± 38.95 | 0.34 ± 0.15 |
| c189996_g1_i1 | Uncharacterized protein | XP_022826974.1 | -446.72 | 11.05 ± 2.94 | 0.02 ± 0.01 |
| c190532_g1_i1 | Uncharacterized protein | XP_021194870.1 | -461.95 | 564.42 ± 153.45 | 1.18 ± 0.20 |
| c84831_g1_i1 | Uncharacterized protein | XP_021186568.1 | -467.37 | 84.41 ± 22.88 | 0.17 ± 0.04 |
| c77816_g1_i1 | Uncharacterized protein | XP_022827450.1 | -490.96 | 114.36 ± 31.13 | 0.22 ± 0.04 |
| c77631_g1_i1 | Pancreatic triacylglycerol lipase-like | XP_022822182.1 | -514.27 | 394.59 ± 107.71 | 0.74 ± 0.10 |
| c131378_g1_i1 | Uncharacterized protein | XP_022832977.1 | -517.35 | 86.13 ± 23.00 | 0.16 ± 0.04 |
| c150661_g1_i1 | Glycine-rich cell wall structural protein | XP_022814028.1 | -517.43 | 845.67 ± 222.60 | 1.62 ± 0.25 |
| c174120_g1_i1 | Lipase member I-like | XP_022835360.1 | -556.62 | 61.53 ± 17.16 | 0.10 ± 0.04 |
| c131293_g1_i1 | Trypsin, alkaline C-like | XP_022814591.1 | -573.35 | 168.27 ± 43.33 | 0.28 ± 0.15 |
| c92167_g1_i1 | Uncharacterized protein | XP_022822319.1 | -637.19 | 21.83 ± 6.03 | 0.03 ± 0.02 |
| c72850_g1_i1 | Uncharacterized protein | XP_022818385.1 | -649.90 | 253.61 ± 73.11 | 0.36 ± 0.18 |
| c79703_g1_i1 | Maltase 2-like | XP_022821250.1 | -715.94 | 52.01 ± 15.58 | 0.07 ± 0.03 |
| c85871_g1_i1 | Uncharacterized protein | XP_022827202.1 | -741.61 | 98.45 ± 28.73 | 0.12 ± 0.02 |
| c83093_g2_i1 | Carboxypeptidase B-like | XP_022818071.1 | -907.00 | 630.13 ± 183.92 | 0.66 ± 0.18 |
| c111975_g1_i1 | Uncharacterized protein | XP_022833083.1 | -980.59 | 136.07 ± 37.52 | 0.13 ± 0.07 |
| c150772_g1_i1 | Glycine-rich cell wall structural protein-like | XP_021194678.1 | -1588.57 | 167.99 ± 44.02 | 0.10 ± 0.06 |
| c72018_g1_i1 | Acyl-coa Delta(11) desaturase-like | XP_022814069.1 | -2237.59 | 292.53 ± 75.31 | 0.13 ± 0.10 |
