# Supplementary material for: A PLA2 deletion mutant using CRISPR/Cas9 coupled to RNASeq reveals insect immune genes associated with eicosanoid signaling
Source: PLoS One. 2024 Jul 17;19(7):e0304958. doi: 10.1371/journal.pone.0304958 (PMC11253937; doi:10.1371/journal.pone.0304958)
Supplement: S5 Table — The genes with at least 50 times fold change (up or down) were selected from 8,944 DEGs in G2 of Fig 4B. (DOCX) [file pone.0304958.s006.docx]

**S5 Table**. G**enes associated with sPLA_2_ in *S. exigua*.** The genes with at least 50 times fold change (up or down) were selected from 8,944 DEGs in G2 of Fig 4B.

| **Contig** | **Gene** | **Gene bank accession number** | **Fold change** | **FPKM** | |
| --- | --- | --- | --- | --- | --- |
|  |  |  |  | **ΔsPLA_2_** | **WT** |
| c62241_g1_i1 | Low-density lipoprotein receptor-related protein 4 | XP_022829670.1 | 391.79 | 12.66 ± 0.19 | 0.02 ± 0.01 |
| c15402_g1_i1 | Desaturase | ARD71184.1 | 99.49 | 11.95 ± 0.45 | 0.10 ± 0.03 |
| c91348_g1_i3 | Membrane-associated guanylate kinase, WW and PDZ domain-containing protein 1 isoform X1 | XP_022836433.1 | 97.37 | 1.43 ± 0.08 | 0.01 ± 0.00 |
| c91855_g1_i1 | WD repeat and FYVE domain-containing protein 3 isoform X4 | XP_022814283.1 | 91.97 | 2.91 ± 0.06 | 0.02 ± 0.00 |
| c82355_g1_i3 | Uncharacterized protein | XP_022823070.1 | 86.25 | 5.01 ± 0.06 | 0.05 ± 0.02 |
| c91605_g1_i1 | LOW QUALITY PROTEIN: multiple epidermal growth factor-like domains protein 8 | XP_022820359.1 | 82.68 | 1.46 ± 0.04 | 0.01 ± 0.00 |
| c90580_g1_i2 | Ran-binding protein 9-like isoform X1 | XP_013192639.1 | 82.05 | 0.80 ± 0.02 | 0.01 ± 0.00 |
| c89047_g1_i1 | Neuroglian | XP_022822103.1 | 81.76 | 22.38 ± 0.91 | 0.15 ± 0.03 |
| c91939_g3_i1 | Dmx-like protein 2 isoform X2 | XP_022824456.1 | 80.07 | 5.58 ± 0.54 | 0.05 ± 0.01 |
| c89684_g2_i2 | Hypothetical protein | PCG74070.1 | 77.44 | 1.88 ± 0.11 | 0.01 ± 0.00 |
| c151374_g1_i1 | Nuclear pore membrane glycoprotein 210 | XP_022822764.1 | 75.60 | 4.66 ± 0.11 | 0.05 ± 0.00 |
| c91780_g1_i1 | Uncharacterized protein | XP_022816803.1 | 75.00 | 36.03 ± 1.80 | 0.31 ± 0.06 |
| c86738_g3_i1 | Retrovirus-related Pol polyprotein from transposon 412-like Protein | EFA13518.1 | 73.56 | 1.16 ± 0.06 | 0.01 ± 0.00 |
| c89576_g4_i1 | Protein KIAA0100 | XP_022819487.1 | 70.46 | 1.67 ± 0.11 | 0.01 ± 0.00 |
| c66727_g1_i1 | Uncharacterized protein | XP_022837608.1 | 69.93 | 2.33 ± 0.13 | 0.02 ± 0.00 |
| c80381_g1_i1 | Protein lunapark-B | XP_022816834.1 | 66.77 | 8.15 ± 0.14 | 0.10 ± 0.01 |
| c90741_g1_i1 | Uncharacterized protein | XP_022835363.1 | 64.70 | 2.37 ± 0.11 | 0.02 ± 0.00 |
| c91195_g1_i3 | GATA zinc finger domain-containing protein 14-like isoform X1 | XP_022818144.1 | 62.33 | 1.01 ± 0.10 | 0.01 ± 0.00 |
| c90569_g1_i1 | Talin-2-like | XP_022836614.1 | 59.19 | 4.08 ± 0.25 | 0.03 ± 0.01 |
| c88728_g2_i1 | 5'-3' exoribonuclease 1-like | XP_022815914.1 | 56.97 | 5.35 ± 0.26 | 0.07 ± 0.02 |
| c91808_g2_i1 | Rho GTPase-activating protein 190 | XP_022835664.1 | 56.32 | 1.64 ± 0.03 | 0.02 ± 0.00 |
| c81811_g1_i1 | N(G),N(G)-dimethylarginine dimethylaminohydrolase 1 | XP_022833144.1 | 55.98 | 2.10 ± 0.11 | 0.02 ± 0.00 |
| c91779_g2_i1 | *Spodoptera litura* probable uridine-cytidine kinase | XM_022966966 | 55.24 | 4.40 ± 0.17 | 0.07 ± 0.03 |
| c88269_g1_i1 | Centrosomal protein of 97 kda isoform X2 | XP_022823663.1 | 54.51 | 2.92 ± 0.05 | 0.04 ± 0.01 |
| c21014_g1_i1 | Vacuolar protein sorting-associated protein 13D | XP_022819401.1 | 53.93 | 3.49 ± 0.05 | 0.05± 0.01 |
| c83094_g1_i1 | Dual specificity protein phosphatase CDC14A-like | XP_022834865.1 | 53.84 | 1.17 ± 0.05 | 0.02 ± 0.00 |
| c90768_g3_i3 | Uncharacterized protein | XP_022827228.1 | 52.94 | 1.77 ± 0.07 | 0.02 ± 0.00 |
| c91127_g1_i1 | eIF-2-alpha kinase GCN2 | XP_022835131.1 | 52.80 | 1.31 ± 0.05 | 0.02 ± 0.00 |
| c91690_g1_i2 | Transformation/transcription domain-associated protein isoform X1 | XP_022821036.1 | 52.40 | 3.48 ± 0.09 | 0.04 ± 0.00 |
| c90007_g1_i1 | Uncharacterized protein LOC105391048 | XP_011560756.1 | 52.24 | 0.59 ± 0.01 | 0.01 ± 0.00 |
| c88551_g1_i2 | LOW QUALITY PROTEIN: uncharacterized protein | XP_022834537.1 | 51.68 | 2.00 ± 0.11 | 0.03 ± 0.00 |
| c80775_g1_i1 | Brachyurin-like | XP_022826846.1] | -50.48 | 1.43 ± 0.20 | 57.98 ± 14.46 |
| c91915_g1_i2 | Zonadhesin-like | XP_022816433.1 | -50.51 | 0.03 ± 0.00 | 1.50 ± 0.57 |
| c90116_g1_i1 | Protein-glucosylgalactosylhydroxylysine glucosidase-like isoform X1 | XP_022821189.1 | -50.71 | 1.17± 0.07 | 55.49 ± 19.3 |
| c81829_g1_i1 | Elongation of very long chain fatty acids protein 7-like isoform X1 | XP_022816094.1 | -50.79 | 0.10 ± 0.05 | 4.63 ± 1.50 |
| c131946_g1_i1 | Uncharacterized protein | XP_022825069.1 | -50.80 | 0.38 ± 0.03 | 17.15 ± 5.20 |
| c91016_g1_i1 | Caspase-1-B-like | XP_022818710.1 | -50.85 | 0.18 ± 0.03 | 7.99 ± 2.38 |
| c87403_g2_i1 | Apolipoprotein D-like | XP_022822062.1 | -51.91 | 0.19 ± 0.05 | 9.24± 3.2 |
| c80865_g1_i1 | Uncharacterized protein | XP_022820807.1 | -51.95 | 0.12 ± 0.00 | 5.42 ± 1.36 |
| c85371_g2_i1 | Venom carboxylesterase-6-like | XP_022822321.1 | -51.98 | 0.08 ± 0.01 | 3.47± 0.91 |
| c82990_g1_i1 | Repat21 | AFH57141.1 | -52.31 | 1.21 ± 0.22 | 55.64 ± 18.15 |
| c86362_g6_i5 | Peptidoglycan-recognition protein LB-like | XP_022831609.1 | -52.34 | 3.76 ± 0.14 | 178.36 ± 58.29 |
| c82190_g1_i1 | Uncharacterized protein | XP_022822474.1 | -52.76 | 0.05 ± 0.01 | 2.58 ± 0.87 |
| c81166_g1_i1 | Uncharacterized protein | XP_022835393.1 | -52.95 | 0.17 ± 0.01 | 7.93 ± 2.17 |
| c82968_g1_i1 | Hypothetical protein | PSN40188.1 | -53.26 | 0.25 ± 0.05 | 12.70 ± 4.32 |
| c76712_g1_i1 | Luciferin 4-monooxygenase-like | XP_022816435.1 | -54.13 | 0.11 ± 0.02 | 5.41 ± 1.46 |
| c84646_g1_i1 | 23 kda integral membrane protein-like | XP_022820135.1 | -54.22 | 2.56 ± 0.04 | 123.63 ± 39.02 |
| c72065_g1_i1 | Repat15 | AFH57135.1 | -54.30 | 1.0 ± 0.06 | 50.73 ± 15.00 |
| c88436_g1_i2 | UDP-glucuronosyltransferase 2C1 | XP_022834356.1 | -55.42 | 0.05 ± 0.00 | 2.4 ± 0.60 |
| c83208_g1_i1 | Chemosensory protein 11 | AKT26487.1 | -55.94 | 0.91 ± 0.06 | 44.60 ± 13.53 |
| c70565_g1_i1 | Myophilin-like | XP_022825129.1 | -56.08 | 3.96± 0.19 | 192.28 ± 57.15 |
| c82283_g1_i1 | Uncharacterized protein | XP_022837314.1 | -56.35 | 0.14 ± 0.02 | 6.73 ± 1.82 |
| c90095_g1_i1 | Xanthine dehydrogenase 1-like | XP_022827163.1 | -57.07 | 0.02 ± 0.00 | 0.91 ± 0.31 |
| c87807_g1_i1 | Sugar transporter ERD6-like 5 | XP_022834207.1 | -57.16 | 0.42 ± 0.03 | 21.63 ± 6.76 |
| c75999_g1_i1 | Circadian clock-controlled protein-like | XP_022828311.1 | -57.31 | 32.13 ± 1.32 | 1711.08 ± 602.57 |
| c88254_g3_i1 | Uncharacterized protein | XP_022831959.1 | -57.89 | 0.15 ± 0.04 | 7.67 ± 2.77 |
| c53473_g1_i1 | Glycine-rich cell wall structural protein 1.0-like | XM_022970919 | -58.60 | 0.44 ± 0.04 | 22.56 ± 6.86 |
| c86049_g1_i1 | Uncharacterized protein | XP_022817893.1 | -59.09 | 0.36 ± 0.02 | 18.43 ± 5.31 |
| c88580_g1_i1 | Alpha-tocopherol transfer protein-like | XP_022820774.1 | -59.64 | 0.60 ± 0.09 | 31.83 ± 10.42 |
| c89650_g3_i3 | Lipase 3-like | XP_022821979.1 | -60.19 | 3.10 ± 0.48 | 173.80 ± 61.05 |
| c82263_g2_i4 | Uncharacterized protein | XP_022817891.1 | -60.33 | 0.12 ± 0.01 | 6.21 ± 1.6 |
| c79263_g2_i3 | Sodium/potassium/calcium exchanger 4-like | XP_022832744.1 | -60.38 | 0.04 ± 0.01 | 2.43 ± 0.88 |
| c76172_g1_i1 | H/ACA ribonucleoprotein complex subunit 1-like | XP_022834075.1 | -62.06 | 0.85 ± 0.17 | 47.44 ± 16.52 |
| c85605_g1_i1 | Uncharacterized protein | XP_022816237.1 | -62.31 | 0.29 ± 0.07 | 16.13 ± 5.06 |
| c87177_g3_i2 | Pancreatic lipase-related protein 2-like | XP_022822262.1 | -62.31 | 0.02 ± 0.00 | 1.05 ± 0.34 |
| c20471_g1_i1 | Uncharacterized protein OBRU01_14834 | KOB70863.1 | -62.78 | 0.14 ± 0.03 | 7.64 ± 2.43 |
| c91053_g2_i1 | Gelsolin, cytoplasmic-like | XP_022831340.1 | -63.49 | 0.34 ± 0.01 | 17.98 ± 4.76 |
| c87870_g1_i2 | Phospholipase A1 member A-like | XP_021201376.1 | -63.69 | 0.04 ± 0.01 | 2.35± 0.68 |
| c73850_g1_i1 | Uncharacterized protein | XP_022814617.1 | -64.39 | 0.09 ± 0.02 | 5.46 ± 1.67 |
| c89758_g3_i2 | Hypothetical protein | PCG76268.1 | -64.77 | 1.39 ± 0.02 | 81.15 ± 26.14 |
| c92153_g1_i1 | Testis-specific gene A8 protein-like | XP_022830264.1 | -64.93 | 0.82 ± 0.11 | 45.15 ± 12.87 |
| c85883_g1_i1 | Facilitated trehalose transporter Tret1-like | XP_022835083.1 | -65.35 | 0.04 ± 0.01 | 2.47 ± 0.88 |
| c71893_g1_i1 | Trypsin II-P29-like | XP_022824767.1 | -66.26 | 0.78 ± 0.04 | 42.47 ± 10.66 |
| c88841_g7_i1 | 41B01_habac_fin, Helicoverpa armigera BAC, pupae DNA | FP340437 | -66.39 | 0.88 ± 0.35 | 52.79 ± 18.57 |
| c69275_g1_i1 | Lactase-phlorizin hydrolase-like | XP_022818655.1 | -67.24 | 0.08 ± 0.01 | 4.65 ± 1.26 |
| c89749_g1_i1 | Uncharacterized protein | XP_022815766.1 | -69.29 | 0.65 ± 0.05 | 38.57 ± 10.74 |
| c80152_g1_i1 | Uncharacterized protein | XP_022815078.1 | -69.34 | 2.39 ± 0.50 | 137.43 ± 39.04 |
| c83826_g1_i1 | Uncharacterized protein | XP_022819827.1 | -70.05 | 0.64 ± 0.14 | 36.55 ± 9.58 |
| c88107_g1_i1 | Carbohydrate sulfotransferase 4-like | XP_022829420.1 | -70.12 | 0.05 ± 0.02 | 3.40 ± 0.87 |
| c191473_g1_i1 | Chemosensory protein 20 | AKT26494.1 | -70.77 | 0.61 ± 0.09 | 36.84 ± 10.40 |
| c85594_g1_i1 | Insecticyanin-B-like | XP_022817843.1 | -71.84 | 1.31 ± 0.13 | 83.08 ± 24.70 |
| c75857_g1_i1 | Cuticle protein 6.4-like | XP_022830150.1 | -72.25 | 2.00 ± 0.13 | 123.62 ± 41.14 |
| c88191_g1_i1 | Facilitated trehalose transporter Tret1-like | XP_022815553.1 | -73.44 | 0.08 ± 0.03 | 5.20 ± 1.45 |
| c90757_g2_i1 | Serine protease inhibitor dipetalogastin isoform X1 | XP_022834209.1 | -73.59 | 0.07 ± 0.01 | 4.55 ± 1.09 |
| c79504_g1_i1 | Viral-like chitinase | ABA06504.1 | -73.90 | 0.36 ± 0.04 | 22.88 ± 6.5 |
| c73975_g1_i1 | Endocuticle structural glycoprotein ABD-5-like | XP_021200996.1 | -74.02 | 0.66 ± 0.10 | 43.27 ± 13.49 |
| c83345_g2_i2 | LOW QUALITY PROTEIN: uncharacterized protein | XP_021184237.1 | -74.27 | 0.85 ± 0.03 | 53.56 ± 15.02 |
| c89004_g3_i1 | Uncharacterized protein | XP_022835049.1 | -75.36 | 0.05 ± 0.00 | 3.32 ± 0.89 |
| c75862_g1_i1 | Hypothetical protein | PCG72336.1 | -75.72 | 0.08 ± 0.01 | 5.01 ± 1.27 |
| c81572_g4_i1 | Diapausin B1 | AKJ54517.1 | -76.88 | 65.20 ± 2.82 | 4466.35 ± 1477.63 |
| c87120_g2_i1 | Alpha-tocopherol transfer protein-like | XP_022832150.1 | -78.29 | 0.10 ± 0.02 | 6.82 ± 2.0 |
| c40199_g1_i1 | Transmembrane protease serine 9-like | XP_022827086.1 | -78.42 | 0.59 ± 0.03 | 38.77 ± 10.58 |
| c89952_g3_i1 | Mucin-2-like | XP_022823928.1 | -79.42 | 0.10 ± 0.01 | 7.45 ± 2.71 |
| c72554_g1_i1 | Uncharacterized protein | XP_022826736.1 | -81.22 | 0.06 ± 0.01 | 4.58 ± 1.26 |
| c74251_g1_i1 | Epididymal secretory protein E1-like | XP_022817737.1 | -82.09 | 1.95 ± 0.06 | 144.10 ± 46.53 |
| c88066_g2_i1 | Ll4 | AKP99432.1 | -82.67 | 0.53 ± 0.08 | 38.92 ± 11.76 |
| c89833_g1_i1 | Glucosylceramidase-like isoform X2 | XP_022832098.1 | -82.72 | 0.31 ± 0.02 | 23.37 ± 7.69 |
| c83863_g2_i1 | Repat35 | AFH57155.1 | -84.16 | 1.74 ± 0.14 | 124.02 ± 34.73 |
| c81125_g1_i2 | Repat19 | AFH57139.1 | -84.34 | 0.41 ± 0.04 | 30.46 ± 8.96 |
| c83232_g1_i1 | Uncharacterized | XM_022959770 | -84.36 | 0.09 ± 0.03 | 7.25 ± 2.47 |
| c85897_g2_i1 | Sodium-dependent nutrient amino acid transporter 1-like | XP_022830993.1 | -84.63 | 0.28 ± 0.03 | 20.96 ± 6.10 |
| c86173_g1_i1 | Fibroin heavy chain-like | XP_022821902.1 | -84.87 | 0.32 ± 0.01 | 22.78 ± 5.53 |
| c74815_g1_i1 | Glucosylceramidase-like | XP_013142523.1 | -85.21 | 0.06 ± 0.02 | 5.33± 1.87 |
| c79072_g1_i2 | Uncharacterized protein | XP_022832336.1 | -85.28 | 0.11 ± 0.01 | 7.95 ± 2.44 |
| c113540_g1_i1 | Intestinal mucin | ABW06596.1 | -85.81 | 0.72 ± 0.06 | 53.49 ± 16.41 |
| c89879_g1_i1 | Alpha-N-acetylgalactosaminidase-like | XP_022816887.1 | -86.74 | 0.15 ± 0.0 | 11.49 ± 3.08 |
| c86163_g1_i1 | Uncharacterized protein | XP_022826749.1 | -87.80 | 0.19 ± 0.05 | 15.39± 4.76 |
| c88384_g1_i1 | Alpha-tocopherol transfer protein-like | XP_022832175.1 | -88.42 | 0.08 ± 0.02 | 7.10 ± 2.47 |
| c73458_g1_i1 | Hypothetical protein | PCG79382.1 | -89.53 | 0.94 ± 0.07 | 72.45 ± 20.84 |
| c81088_g1_i1 | Uncharacterized protein | XP_022832512.1 | -89.59 | 0.29 ± 0.07 | 21.74 ± 6.08 |
| c83846_g1_i1 | Uncharacterized protein | XP_022817386.1 | -91.60 | 0.073 ± 0.02 | 6.00 ± 2.23 |
| c82225_g1_i1 | Odorant binding protein | ADY17884.1 | -92.69 | 2.39 ± 0.01 | 198.65 ± 64.31 |
| c86472_g1_i2 | Uncharacterized protein | XP_022834551.1 | -93.45 | 0.07 ± 0.02 | 5.82 ± 1.89 |
| c83294_g1_i1 | Uncharacterized protein | XP_022826995.1 | -93.70 | 0.24 ± 0.03 | 19.37 ± 5.67 |
| c82141_g1_i1 | Androgen-dependent TFPI-regulating protein-like | XP_022815150.1 | -94.45 | 0.36 ± 0.07 | 31.70 ± 11.37 |
| c89779_g3_i1 | Fatty acid-binding protein, liver-like | XP_022830919.1 | -94.64 | 0.40 ± 0.17 | 31.51 ± 8.98 |
| c87523_g2_i1 | Organic cation transporter protein-like | XP_022828133.1 | -95.03 | 0.15 ± 0.04 | 13.39 ± 4.20 |
| c86121_g1_i1 | Uncharacterized protein | XP_022831880.1 | -95.71 | 0.07± 0.02 | 6.34 ± 2.10 |
| c84776_g2_i1 | Uncharacterized protein | XP_022834170.1 | -96.22 | 0.28 ± 0.18 | 23.56 ± 6.95 |
| c85156_g1_i1 | Lactase-phlorizin hydrolase-like | XP_022827201.1 | -96.42 | 0.10 ± 0.03 | 8.71 ± 2.90 |
| c85592_g1_i1 | Uncharacterized protein | XP_022822432.1 | -97.42 | 0.07 ± 0.03 | 6.44 ± 1.70 |
| c86646_g1_i1 | Trypsin, alkaline B-like | XP_022836358.1 | -97.47 | 0.28 ± 0.06 | 22.88 ± 5.96 |
| c84426_g1_i1 | Bromodomain-containing protein DDB_G0280777-like | XP_022815639.1 | -97.71 | 0.10 ± 0.01 | 9.16 ± 2.79 |
| c77762_g1_i2 | Dentin sialophosphoprotein-like | XP_022829288.1 | -99.52 | 0.09 ± 0.00 | 8.10 ± 2.73 |
| c76799_g1_i1 | Collagen alpha-1(IV) chain-like | XP_022828198.1 | -99.53 | 0.68 ± 0.17 | 63.55 ± 22.85 |
| c83355_g1_i1 | Ll7 | AQX37242.1 | -99.67 | 0.53 ± 0.05 | 44.40 ± 11.98 |
| c87748_g11_i1 | LOW QUALITY PROTEIN: signal peptidase complex subunit 3 | XP_021183812.1 | -99.69 | 0.49 ± 0.20 | 42.69 ± 11.94 |
| c90789_g1_i1 | Peroxidase | XP_022826162.1 | -99.91 | 0.06 ± 0.02 | 5.61 ± 1.41 |
| c81979_g1_i1 | Regucalcin-like | XP_022815254.1 | -100.17 | 0.40 ± 0.00 | 33.99 ± 9.07 |
| c86481_g2_i1 | Serpin-Z4-like | XP_022837525.1 | -100.36 | 0.19 ± 0.04 | 17.36 ± 5.09 |
| c85297_g1_i1 | Ctenidin-3-like | XP_022834067.1 | -100.38 | 0.20 ± 0.09 | 18.16 ± 5.79 |
| c87299_g1_i1 | Glucose oxidase | ADL38963.1 | -100.63 | 0.60 ± 0.03 | 51.92 ± 14.61 |
| c77644_g1_i1 | Uncharacterized protein | XP_021181157.1 | -100.88 | 0.08 ± 0.02 | 7.26 ± 2.03 |
| c89285_g1_i1 | Lachesin isoform X1 | XP_022832190.1 | -100.89 | 0.02 ± 0.01 | 1.90 ± 0.62 |
| c86064_g1_i1 | Estrogen sulfotransferase-like | XP_022819760.1 | -101.69 | 0.79 ± 0.12 | 77.54 ± 28.32 |
| c88121_g1_i1 | Sensory neuron membrane protein 2 | AGN52677.1 | -101.93 | 0.51 ± 0.07 | 49.50 ± 17.51 |
| c87780_g1_i1 | Cuticle protein 8-like | XP_022815933.1 | -102.30 | 0.18 ± 0.04 | 14.94 ± 4.08 |
| c171177_g1_i1 | Uncharacterized protein | XP_022829990.1 | -102.54 | 0.19 ± 0.05 | 17.09 ± 4.44 |
| c79269_g1_i1 | Uncharacterized protein | XP_022831879.1 | -102.96 | 0.47 ± 0.05 | 39.99 ± 10.15 |
| c78082_g1_i1 | Fibroin heavy chain-like | XP_022822011.1 | -105.16 | 1.36 ± 0.04 | 119.94 ± 32.42 |
| c59988_g1_i1 | Cuticle protein-like | XP_022824787.1 | -106.18 | 0.12 ± 0.03 | 10.99 ± 2.81 |
| c190293_g1_i1 | Pro-resilin-like | XP_022826991.1 | -106.69 | 0.91 ± 0.07 | 81.65 ± 22.35 |
| c72186_g2_i2 | Tetra-peptide repeat homeobox protein 1-like | XP_022826659.1 | -107.69 | 0.03 ± 0.00 | 3.11 ± 0.89 |
| c61037_g1_i1 | Larval cuticle protein LCP-17-like | XP_022816666.1 | -107.89 | 12.95 ± 0.17 | 1192.36 ± 348.08 |
| c92193_g1_i1 | Uncharacterized protein | XP_022830831.1 | -109.28 | 2.57 ± 0.10 | 248.26 ± 77.79 |
| c86439_g2_i1 | Decaprenyl-diphosphate synthase subunit 2 | XP_022816493.1 | -110.22 | 0.04 ± 0.01 | 3.88 ± 1.05 |
| c88441_g1_i1 | Antennal esterase CXE10 | AEJ38207.1 | -110.41 | 0.07 ± 0.02 | 7.58 ± 2.50 |
| c71544_g1_i1 | Uncharacterized, ncrna | XR_002696828 | -110.96 | 0.944 ± 0.19 | 90.99 ± 31.18 |
| c88652_g1_i1 | Polycalin | ANC90402.1 | -111.13 | 2.73 ± 0.08 | 253.96 ± 67.03 |
| c16441_g1_i1 | Carboxypeptidase B-like | XP_022838055.1 | -111.69 | 0.07 ± 0.00 | 6.88 ± 1.9 |
| c132630_g1_i1 | RNA-binding protein 33-like | XP_022829656.1 | -111.76 | 0.18 ± 0.04 | 18.07 ± 4.79 |
| c84818_g1_i1 | Serine proteinase stubble | XP_022817110.1 | -112.33 | 0.04 ± 0.01 | 3.55 ± 0.93 |
| c72952_g1_i1 | Uncharacterized protein | XP_022820339.1 | -112.72 | 0.46 ± 0.01 | 43.31 ± 11.55 |
| c15325_g1_i1 | Hypothetical protein | PCG70881.1 | -112.95 | 0.07 ± 0.02 | 7.21 ± 1.94 |
| c92545_g1_i1 | Protein obstructor-E | XP_022822561.1 | -113.53 | 0.63 ± 0.12 | 60.51 ± 15.67 |
| c62474_g1_i1 | Hypothetical protein | PCG66042.1 | -113.93 | 0.24 ± 0.08 | 23.83 ± 7.74 |
| c85942_g1_i1 | Alpha-tocopherol transfer protein | XP_022821051.1 | -114.12 | 0.33 ± 0.03 | 31.28 ± 8.45 |
| c87764_g2_i1 | Carboxypeptidase B-like | XP_022832131.1 | -116.28 | 0.15 ± 0.04 | 14.27 ± 3.44 |
| c85537_g1_i1 | Uncharacterized protein | XP_022817879.1 | -116.65 | 2.42 ± 0.07 | 235.99 ± 65.17 |
| c77276_g1_i1 | Spodoptera litura shematrin-like protein 1 | XM_022972603 | -117.94 | 0.05 ± 0.00 | 4.83 ± 1.31 |
| c38238_g1_i1 | Brachyurin-like | XP_022824739.1 | -118.43 | 0.29 ± 0.11 | 31.26 ± 9.61 |
| c83103_g1_i1 | Larval/pupal cuticle protein H1C-like | XP_022830351.1 | -118.67 | 0.56 ± 0.09 | 58.65 ± 18.18 |
| c77464_g1_i1 | Leukocyte surface antigen CD53-like | XP_022823982.1 | -119.40 | 0.40 ± 0.05 | 39.13 ± 10.32 |
| c89646_g1_i1 | Juvenile hormone esterase-like | XP_022827863.1 | -120.83 | 0.11 ± 0.02 | 11.66 ± 3.37 |
| c72018_g1_i1 | Acyl-coa Delta(11) desaturase-like | XP_022814069.1 | -122.63 | 2.91 ± 0.26 | 292.53 ± 75.31 |
| c86402_g5_i1 | Facilitated trehalose transporter Tret1-like | XP_022827346.1 | -123.20 | 0.13 ± 0.02 | 15.61 ± 5.4 |
| c83086_g1_i1 | Aminopeptidase N | AAT99437.1 | -125.88 | 0.46 ± 0.02 | 49.99 ± 14.01 |
| c89005_g1_i1 | Putative fatty acyl-coa reductase CG5065 | XP_022824237.1 | -127.87 | 0.03 ± 0.00 | 3.29 ± 0.86 |
| c37951_g1_i1 | Takeout | ATU07277.1 | -128.36 | 0.05 ± 0.02 | 6.15 ± 1.75 |
| c112566_g1_i1 | Fatty acyl-coa reductase wat-like | XP_022837119.1 | -130.64 | 0.22 ± 0.03 | 24.70 ± 6.90 |
| c85986_g1_i1 | Somatomedin-B and thrombospondin type-1 domain-containing protein-like | XP_022832025.1 | -136.09 | 0.20 ± 0.01 | 23.04 ± 6.04 |
| c185907_g1_i1 | Spodoptera litura cuticle protein 16.5-like | XM_022974499 | -136.38 | 0.10 ± 0.03 | 12.08 ± 3.16 |
| c77112_g1_i1 | Larval cuticle protein 1-like | XP_022816717.1 | -136.62 | 40.37 ± 3.367 | 4817.87 ± 1510.74 |
| c76571_g1_i1 | Revip | AGZ92264.1 | -139.65 | 0.83 ± 0.07 | 106.07 ± 37.54 |
| c89146_g4_i2 | Uncharacterized protein | XP_022835587.1 | -141.68 | 0.12 ± 0.01 | 14.76 ± 3.88 |
| c90753_g1_i1 | Uncharacterized protein | XP_022815519.1 | -142.53 | 0.08 ± 0.01 | 10.17 ± 3.23 |
| c85919_g1_i1 | Uncharacterized protein | XP_022824726.1 | -143.61 | 0.91 ± 0.04 | 111.51 ± 31.57 |
| c52918_g1_i1 | Uncharacterized protein | XP_022826722.1 | -144.10 | 0.10 ± 0.00 | 12.39 ± 3.51 |
| c16743_g1_i1 | Uncharacterized protein | XP_022825567.1 | -145.07 | 0.09 ± 0.03 | 12.31 ± 4.34 |
| c81276_g1_i1 | Inositol oxygenase-like | XP_022832157.1 | -145.15 | 0.50 ± 0.07 | 63.05 ± 19.09 |
| c80128_g1_i1 | Glycine-rich protein 3-like | XP_022834085.1 | -145.41 | 0.43 ± 0.02 | 60.02 ± 22.53 |
| c88277_g1_i1 | Protein takeout-like | XP_022828230.1 | -146.59 | 0.19 ± 0.03 | 24.91 ± 7.80 |
| c49886_g1_i1 | Laccase-1 | XP_022819652.1 | -147.22 | 0.06 ± 0.02 | 7.21 ± 1.96 |
| c94524_g1_i1 | Uncharacterized protein | XP_022825552.1 | -147.46 | 0.260 ± 0.03 | 32.06 ± 8.32 |
| c87397_g1_i3 | Fibrohexamerin-like | XP_022817133.1 | -149.39 | 0.11 ± 0.00 | 15.65 ± 5.47 |
| c88751_g1_i1 | Pro-resilin | XP_022826231.1 | -150.08 | 0.04 ± 0.00 | 5.62 ± 1.47 |
| c83684_g2_i1 | Myrosinase 1-like | XP_022822385.1 | -152.02 | 0.46 ± 0.04 | 59.82 ± 16.88 |
| c86423_g2_i2 | Uncharacterized protein | XP_022823921.1 | -154.04 | 0.10 ± 0.01 | 14.58 ± 4.58 |
| c85147_g1_i1 | Bile salt-activated lipase-like | XP_022822371.1 | -157.24 | 0.11 ± 0.03 | 15.61 ± 4.77 |
| c154405_g1_i1 | Circadian clock-controlled protein-like | XP_021182291.1 | -157.40 | 0.10 ± 0.02 | 13.69 ± 3.80 |
| c82529_g1_i1 | Glutathione S-transferase 1-like | XP_022815917.1 | -157.75 | 0.23 ± 0.03 | 30.81± 9.20 |
| c83210_g1_i2 | Acanthoscurrin-2-like isoform X1 | XP_022822414.1 | -159.57 | 1.13± 0.13 | 167.40± 59.08 |
| c79187_g1_i1 | Uncharacterized protein | XP_022827657.1 | -159.76 | 0.08 ± 0.018 | 11.43 ± 3.35 |
| c131356_g1_i1 | Peroxidase-like | XP_022816507.1 | -162.42 | 0.12 ± 0.01 | 17.63 ± 5.35 |
| c79986_g1_i1 | Protein obstructor-E-like | XP_022824511.1 | -162.63 | 0.54 ± 0.07 | 73.16 ± 20.03 |
| c89914_g1_i1 | Solute carrier family 22 member 13-like | XP_022815828.1 | -164.09 | 0.07 ± 0.02 | 11.96 ± 3.66 |
| c84860_g1_i1 | Odorant binding protein 24 | AKT26501.1 | -164.11 | 0.07 ± 0.02 | 10.83 ± 2.94 |
| c64696_g1_i1 | Protein obstructor-E isoform X1 | XP_021194368.1 | -164.55 | 1.63 ± 0.03 | 225.73 ± 62.39 |
| c135224_g1_i1 | Cuticle protein 3-like | XP_022816339.1 | -168.13 | 0.23 ± 0.04 | 33.16 ± 9.24 |
| c89151_g2_i2 | Facilitated trehalose transporter Tret1-like | XP_022836133.1 | -169.06 | 0.02 ± 0.00 | 3.33 ± 1.17 |
| c90892_g1_i1 | Uncharacterized protein | XP_022816686.1 | -172.32 | 0.02 ± 0.00 | 4.57 ± 1.40 |
| c76852_g1_i1 | TSC22 domain family protein 1-like | XP_022828041.1 | -177.12 | 0.26 ± 0.19 | 36.07 ± 9.59 |
| c88296_g1_i1 | Xanthine dehydrogenase-like | XP_022826530.1 | -177.90 | 0.0 ± 0.01 | 4.95 ± 1.37 |
| c80331_g1_i1 | Uncharacterized protein | XP_022832389.1 | -182.56 | 0.35 ± 0.07 | 55.76 ± 15.71 |
| c86248_g1_i1 | Midgut class 1 aminopeptidase N | AAP44964.1 | -182.64 | 0.56 ± 0.00 | 88.33 ± 25.0 |
| c81925_g1_i1 | Solute carrier family 22 member 1-like | XP_022832068.1 | -186.46 | 0.06 ± 0.01 | 10.10 ± 3.18 |
| c14273_g1_i1 | Pollen-specific leucine-rich repeat extensin-like protein 1 | XP_022828128.1 | -188.01 | 0.32 ± 0.08 | 51.71 ± 13.73 |
| c86333_g1_i1 | Innexin inx7-like | XP_022827477.1 | -189.07 | 0.12 ± 0.02 | 20.68 ± 6.43 |
| c81366_g1_i2 | Uncharacterized protein | XP_022834890.1 | -191.11 | 0.40 ± 0.07 | 64.80 ± 17.54 |
| c87160_g2_i1 | Facilitated trehalose transporter Tret1-like | XP_022837452.1 | -192.62 | 0.05 ± 0.00 | 9.43± 3.16 |
| c83233_g1_i1 | Endocuticle structural glycoprotein sgabd-5-like isoform X1 | XP_022816395.1 | -194.70 | 0.45 ± 0.04 | 76.05 ± 22.99 |
| c79461_g2_i1 | Larval cuticle protein LCP-22-like | XP_022816174.1 | -195.36 | 0.82 ± 0.30 | 129.81 ± 35.44 |
| c86103_g1_i1 | Uncharacterized protein LOC111353287 | XP_022822014.1 | -196.75 | 1.15 ± 0.10 | 204.98 ± 66.48 |
| c131481_g1_i1 | Uncharacterized protein | XP_022821909.1 | -201.63 | 0.07 ± 0.03 | 12.65± 3.64 |
| c81997_g1_i1 | Protein obstructor-E-like | XP_022823095.1 | -202.40 | 0.77 ± 0.09 | 132.74 ± 36.67 |
| c73849_g1_i1 | Uncharacterized protein | XP_022822390.1 | -202.42 | 0.10± 0.03 | 19.49 ± 6.47 |
| c68248_g1_i1 | Uncharacterized, ncrna | XR_002697283 | -207.71 | 0.82 ± 0.11 | 146.79 ± 44.12 |
| c87574_g1_i3 | Histone acetyltransferase p300-like | XP_022826940.1 | -211.78 | 0.14 ± 0.02 | 24.92 ± 6.76 |
| c84894_g1_i1 | Circadian clock-controlled protein-like | XP_022828097.1 | -212.91 | 3.75 ± 0.40 | 747.83 ± 261.93 |
| c71380_g1_i1 | Uncharacterized protein | XP_022830994.1 | -214.09 | 0.05 ± 0.00 | 9.23 ± 3.05 |
| c69668_g1_i1 | Collagen alpha-1(IV) chain-like | XP_022828198.1 | -216.26 | 1.44 ± 0.43 | 266.76 ± 87.50 |
| c9361_g1_i1 | Protein obstructor-E-like | XP_022828486.1 | -219.46 | 0.17 ± 0.04 | 34.07± 9.98 |
| c80007_g3_i2 | Trypsin, alkaline C-like | XP_022821670.1 | -223.73 | 1.37 ± 0.10 | 252.55± 64.67 |
| c86554_g1_i1 | Midgut class 4 aminopeptidase N | AAP44967.1 | -228.58 | 0.25 ± 0.04 | 50.47 ± 13.94 |
| c82813_g1_i1 | Larval/pupal cuticle protein H1C-like | XP_022830129.1 | -232.07 | 0.55 ± 0.12 | 118.47 ± 39.64 |
| c83677_g1_i1 | Ejaculatory bulb-specific protein 3-like | XP_021190501.1 | -236.66 | 0.13 ± 0.02 | 26.90 ± 6.91 |
| c76801_g1_i1 | Endocuticle structural glycoprotein ABD-5-like | XP_022817375.1 | -237.35 | 0.47 ± 0.11 | 95.93 ± 26.15 |
| c81166_g2_i1 | Uncharacterized protein | XP_022835392.1 | -243.61 | 0.39 ± 0.12 | 80.05 ± 20.42 |
| c80037_g1_i1 | Cytochrome b5-like | XP_022823555.1 | -246.53 | 0.03 ± 0.01 | 7.01 ± 1.8 |
| c92954_g1_i1 | Synaptonemal complex protein 4 isoform X2 | XP_022835391.1 | -248.26 | 0.27 ± 0.11 | 55.63 ± 14.86 |
| c79609_g1_i1 | Chemosensory protein 19 | AKT26493.1 | -250.24 | 0.09 ± 0.01 | 20.04 ± 6.37 |
| c86041_g1_i1 | Phospholipase A1-like | XP_022814428.1 | -250.96 | 0.05 ± 0.01 | 13.12 ± 4.82 |
| c92528_g1_i1 | Proline-rich protein 4-like | XP_022825189.1 | -252.10 | 0.52 ± 0.03 | 110.07 ± 30.44 |
| c12854_g1_i1 | Pollen-specific leucine-rich repeat extensin-like protein 4 | XM_022964535 | -256.52 | 0.27 ± 0.08 | 61.92 ± 17.85 |
| c190532_g1_i1 | Uncharacterized protein | XP_021194870.1 | -257.96 | 2.65 ± 0.27 | 564.42 ± 153.45 |
| c81715_g3_i2 | Glucose dehydrogenase [FAD, quinone]-like | XP_022831333.1 | -260.19 | 0.08 ± 0.02 | 18.01± 5.25 |
| c190117_g1_i1 | Seminal metalloprotease 1-like | XP_022825106.1 | -261.17 | 0.34 ± 0.09 | 84.01 ± 27.39 |
| c75587_g1_i1 | TSC22 domain family protein 1-like | XP_022828041.1 | -265.91 | 0.42 ± 0.11 | 93.16 ± 25.83 |
| c73601_g2_i1 | Uncharacterized protein | KOB74345.1 | -274.75 | 0.58 ± 0.13 | 145.31 ± 47.12 |
| c88495_g1_i1 | Arylsulfatase J-like | XP_022825369.1 | -281.27 | 0.04 ± 0.00 | 12.08 ± 3.83 |
| c87097_g2_i1 | Uncharacterized, ncrna | XR_002697285 | -282.17 | 0.33 ± 0.23 | 73.63 ± 21.85 |
| c85535_g1_i1 | Lipase 1-like | XP_022831142.1 | -282.99 | 0.39 ± 0.02 | 101.64 ± 33.94 |
| c12854_g2_i1 | Pollen-specific leucine-rich repeat extensin-like protein 4 | XP_022820303.1 | -289.62 | 0.27 ± 0.11 | 63.96 ± 16.31 |
| c84716_g3_i1 | Dentin sialophosphoprotein-like | XP_022829288.1 | -289.63 | 0.29± 0.08 | 74.67 ± 22.39 |
| c83464_g1_i1 | Uncharacterized protein | XP_022824824.1 | -291.40 | 0.03 ± 0.01 | 7.52 ± 1.86 |
| c40113_g1_i1 | Skin secretory protein xp2-like | XP_022825188.1 | -294.78 | 0.13 ± 0.03 | 31.22 ± 8.24 |
| c88143_g2_i3 | Membrane-bound alkaline phosphatase-like | XP_022822920.1 | -297.15 | 0.05 ± 0.02 | 14.4 ± 4.20 |
| c79958_g1_i1 | Flexible cuticle protein 12-like | XP_022825649.1 | -298.32 | 1.6± 0.32 | 401.82 ± 117.19 |
| c86001_g1_i1 | Fatty acid synthase-like | XP_022831847.1 | -302.15 | 0.04 ± 0.01 | 11.24 ± 2.95 |
| c86370_g1_i1 | Uncharacterized protein | XP_022832710.1 | -302.74 | 0.11 ± 0.00 | 30.00 ± 8.12 |
| c114645_g1_i1 | Organic cation transporter protein-like | XP_022828278.1 | -317.07 | 0.03 ± 0.00 | 9.26 ± 3.06 |
| c84724_g1_i1 | Aminoacylase-1A-like | XP_022815042.1 | -331.14 | 0.13 ± 0.01 | 39.88 ± 12.5 |
| c87210_g1_i1 | Myrosinase 1-like isoform X1 | XP_022818244.1 | -354.07 | 0.02 ± 0.00 | 5.55 ± 1.72 |
| c84831_g1_i1 | Uncharacterized protein | XP_021186568.1 | -360.32 | 0.28 ± 0.03 | 84.41 ± 22.88 |
| c82205_g2_i1 | Uncharacterized protein | XP_022837598.1 | -366.17 | 0.22± 0.05 | 69.86 ± 18.59 |
| c67349_g1_i1 | Glycine-rich cell wall structural protein-like | XM_022978304 | -377.49 | 0.43 ± 0.13 | 133.12 ± 35.85 |
| c87843_g4_i1 | Endocuticle structural glycoprotein sgabd-8-like | XP_022816372.1 | -398.95 | 0.22 ± 0.03 | 74.87 ± 20.51 |
| c84846_g1_i1 | Uncharacterized protein | XP_022822932.1 | -401.32 | 1.36 ± 0.07 | 480.08 ± 145.42 |
| c84511_g1_i1 | 23 kda integral membrane protein-like | XP_022837609.1 | -430.25 | 0.27 ± 0.10 | 95.61 ± 27.96 |
| c79703_g1_i1 | Maltase 2-like | XP_022821250.1 | -445.79 | 0.13 ± 0.06 | 52.01 ± 15.58 |
| c78983_g2_i1 | Uncharacterized protein | XP_022832989.1 | -449.04 | 0.34 ± 0.05 | 127.93± 36.00 |
| c68876_g1_i1 | Uncharacterized, ncrna | XR_002697108 | -456.29 | 1.32 ± 0.06 | 508.38 ± 161.44 |
| c76912_g1_i1 | Larval cuticle protein LCP-17-like | XP_022814059.1 | -466.32 | 12.54 ± 0.34 | 5016.24 ± 1530.78 |
| c174120_g1_i1 | Lipase member I-like | XP_022835360.1 | -480.21 | 0.14± 0.05 | 61.53 ± 17.16 |
| c81090_g1_i1 | Fatty acid-binding protein 1-like | XP_022831666.1 | -493.10 | 1.42 ± 0.19 | 602.94 ± 178.79 |
| c86311_g1_i1 | Acyl-coa Delta(11) desaturase-like | XP_022824753.1 | -504.26 | 0.13 ± 0.01 | 57.59 ± 15.55 |
| c89257_g1_i1 | Organic cation transporter protein-like | XP_022828223.1 | -510.81 | 0.03 ± 0.01 | 11.87 ± 3.42 |
| c54184_g1_i1 | Fatty acid-binding protein 1-like | XP_022831663.1 | -521.10 | 2.06 ± 0.09 | 912.63 ± 265.75 |
| c81523_g1_i1 | Uncharacterized protein | XP_022824823.1 | -524.48 | 0.04 ± 0.00 | 19.67 ± 5.87 |
| c86523_g5_i1 | Glycine-rich protein DOT1-like | XP_022837431.1 | -544.31 | 0.22± 0.00 | 101.13 ± 25.72 |
| c83732_g1_i1 | Protein split ends-like | XP_022837898.1 | -566.43 | 0.06 ± 0.02 | 33.38 ± 9.03 |
| c85234_g1_i1 | Uncharacterized protein | XP_022828370.1 | -595.43 | 0.22 ± 0.03 | 126.23 ± 46.43 |
| c78116_g1_i1 | Uncharacterized protein | KOB64398.1 | -612.51 | 0.13 ± 0.02 | 73.45 ± 22.78 |
| c81169_g1_i1 | Circadian clock-controlled protein-like | XP_022828332.1 | -660.22 | 0.13 ± 0.04 | 77.91 ± 24.77 |
| c131293_g1_i1 | Trypsin, alkaline C-like | XP_022814591.1 | -709.64 | 0.28 ± 0.03 | 168.27 ± 43.33 |
| c11650_g1_i1 | Glucose dehydrogenase [FAD, quinone]-like | XP_022828848.1 | -715.45 | 0.02 ± 0.00 | 16.04 ± 4.38 |
| c86041_g2_i2 | Phospholipase A1-like | XP_022814428.1 | -764.43 | 0.06 ± 0.02 | 46.16 ± 15.92 |
| c83674_g1_i1 | Odorant binding protein 9 | AGH70105.1 | -767.29 | 0.25 ± 0.06 | 179.63 ± 59.55 |
| c150772_g1_i1 | Glycine-rich cell wall structural protein-like | XP_021194678.1 | -811.27 | 0.24 ± 0.09 | 167.9 ± 44.02 |
| c76641_g1_i1 | Repat33 | AFH57153.1 | -818.65 | 0.05± 0.00 | 37.29 ± 12.44 |
| c83322_g1_i1 | Circadian clock-controlled protein-like | XP_022829654.1 | -829.42 | 0.45 ± 0.04 | 345.33 ± 121.34 |
| c88081_g2_i1 | Uncharacterized protein | XP_022828345.1 | -830.53 | 0.15 ± 0.00 | 109.66 ± 31.24 |
| c74746_g1_i1 | Endocuticle structural glycoprotein sgabd-2-like | XP_022816544.1 | -836.17 | 0.29± 0.07 | 212.64 ± 61.53 |
| c79176_g1_i1 | Uncharacterized protein | XP_022821338.1 | -842.17 | 0.07 ± 0.02 | 52.69 ± 17.52 |
| c85335_g1_i1 | Hypothetical protein | PCG63726.1 | -929.62 | 0.23 ± 0.01 | 183.61 ± 52.75 |
| c87122_g1_i1 | Serine protease inhibitor dipetalogastin-like | XP_022829250.1 | -930.37 | 0.180 ± 0.01 | 158.10 ± 55.94 |
| c84179_g1_i1 | Uncharacterized protein | XP_022817989.1 | -955.92 | 0.07 ± 0.02 | 61.40 ± 18.37 |
| c67156_g1_i1 | Hypothetical protein | PCG62835.1 | -1005.70 | 0.27 ± 0.05 | 247.08 ± 73.68 |
| c68272_g1_i1 | Uncharacterized protein | XP_022821214.1 | -1128.22 | 0.10 ± 0.04 | 99.15 ± 25.74 |
| c77631_g1_i1 | Pancreatic triacylglycerol lipase-like | XP_022822182.1 | -1128.61 | 0.42 ± 0.04 | 394.59 ± 107.71 |
| c85150_g1_i1 | Larval cuticle protein 16/17-like | XP_022816379.1 | -1304.83 | 1.61 ± 0.25 | 1808.20 ± 544.34 |
| c92167_g1_i1 | Uncharacterized protein DDB_G0290587 | XP_022822319.1 | -1338.49 | 0.01 ± 0.00 | 21.83 ± 6.03 |
| c150661_g1_i1 | Glycine-rich cell wall structural protein | XP_022814028.1 | -1348.32 | 0.74 ± 0.11 | 845.60 ± 222.60 |
| c77816_g1_i1 | Uncharacterized protein | XP_022827450.1 | -1372.55 | 0.09 ± 0.00 | 114.36 ± 31.10 |
| c76574_g1_i1 | Cuticle protein CP14.6-like | XP_022814060.1 | -1392.71 | 0.49 ± 0.13 | 589.5 ± 160.7 |
| c86953_g1_i1 | Uncharacterized protein | XP_022822012.1 | -1394.41 | 0.06 ± 0.02 | 74.5 ± 2.67 |
| c74000_g1_i1 | Uncharacterized protein | XP_022827721.1 | -1470.18 | 0.11 ± 0.01 | 143.9 ± 38.95 |
| c82495_g1_i1 | Larval cuticle protein LCP-30-like | XP_021201007.1 | -1482.08 | 0.06 ± 0.02 | 80.3 ± 22.2 |
| c61763_g2_i1 | Larval cuticle protein LCP-14-like | XP_022814231.1 | -1578.75 | 1.98 ± 0.26 | 2712.07 ± 833.86 |
| c84177_g1_i1 | PREDICTED: mucin-2-like | XP_013195491.1 | -1721.84 | 0.31 ± 0.03 | 465.24 ± 145.58 |
| c82011_g1_i1 | Trypsin, alkaline C-like | XP_022815740.1 | -1760.40 | 2.50 ± 0.03 | 3847.54 ± 1179.61 |
| c12887_g1_i1 | Fatty acid-binding protein 1-like | XP_022831716.1 | -1851.47 | 0.17 ± 0.03 | 266.33 ± 73.54 |
| c88104_g2_i1 | Clone 2J unknown mrna | GU983914 | -1905.45 | 1.96 ± 0.26 | 3152.25 ± 900.41 |
| c77443_g1_i1 | Uncharacterized protein | XP_022817942.1 | -1997.81 | 0.59 ± 0.11 | 996.68 ± 282.19 |
| c131378_g1_i1 | Uncharacterized protein | XP_022832977.1 | -2087.29 | 0.05 ± 0.01 | 86.13 ± 23.0 |
| c80759_g1_i2 | Titin | XP_022837584.1 | -2181.11 | 0.08 ± 0.03 | 142.54 ± 38.60 |
| c85871_g1_i1 | Uncharacterized protein | XP_022827202.1 | -2204.49 | 0.05 ± 0.01 | 98.45 ± 28.73 |
| c151202_g1_i1 | Hypothetical protein | PCG75674.1 | -2341.37 | 0.32 ± 0.05 | 628.38 ± 167.55 |
| c112396_g1_i1 | Flexible cuticle protein 12-like | XP_022825251.1 | -2450.23 | 0.19 ± 0.06 | 421.82 ± 127.06 |
| c72850_g1_i1 | Uncharacterized protein | XP_022818385.1 | -2579.22 | 0.11 ± 0.02 | 253.61 ± 73.11 |
| c112321_g1_i1 | Acyl-coa-binding protein-like | XP_022820273.1 | -2914.96 | 1.17 ± 0.18 | 2945.37 ± 881.88 |
| c80458_g1_i1 | Uncharacterized protein | XP_022817958.1 | -3086.46 | 0.13 ± 0.05 | 357.61 ± 104.43 |
| c73815_g2_i1 | Glycine-rich protein DOT1-like | XP_022819775.1 | -4569.36 | 0.15 ± 0.06 | 556.99 ± 146.71 |
| c111975_g1_i1 | Uncharacterized protein | XP_022833083.1 | -5481.80 | 0.03 ± 0.00 | 136.07 ± 37.52 |
| c87369_g2_i1 | Calphotin-like | XP_022817201.1 | -5933.21 | 0.57 ± 0.07 | 2830.11 ± 789.69 |
| c88270_g9_i1 | Trypsin, alkaline C-like | XP_022815733.1 | -6457.90 | 0.19 ± 0.04 | 1032.77 ± 295.01 |
| c87369_g3_i1 | Calphotin-like | XP_022813970.1 | -8504.72 | 0.28 ± 0.07 | 2122.99 ± 647.18 |
| c83093_g2_i1 | Carboxypeptidase B-like | XP_022818071.1 | -19464.04 | 0.04 ± 0.02 | 630.13 ± 183.92 |
