# Supplementary material for: A PLA2 deletion mutant using CRISPR/Cas9 coupled to RNASeq reveals insect immune genes associated with eicosanoid signaling
Source: PLoS One. 2024 Jul 17;19(7):e0304958. doi: 10.1371/journal.pone.0304958 (PMC11253937; doi:10.1371/journal.pone.0304958)
Supplement: S6 Table — The genes with at least ten times fold change (up or down) were selected from 2,216 DEGs common in both G1 and G2 in Fig 4B. (DOCX) [file pone.0304958.s007.docx]

**S6 Table**. **Immune genes associated with sPLA_2_ in *S. exigua*.** The genes with at least ten times fold change (up or down) were selected from 2,216 DEGs common in both G1 and G2 in Fig 4B.

| **Contig** | **Gene** | **Gene bank accession number** | **Fold change** | **FPKM** | |
| --- | --- | --- | --- | --- | --- |
|  |  |  |  | **WT+ EC** | **ΔsPLA2 + EC** |
| c81246_g1_i1 | Uncharacterized protein | XP_022814982.1 | 72.50 | 19.53 ± 1.05 | 0.17 ± 0.14 |
| c60732_g1_i1 | Anionic antimicrobial peptide 2-like | XP_022826185.1 | 58.86 | 159.03 ± 3.21 | 1.72 ± 0.48 |
| c91348_g1_i3 | Membrane-associated guanylate kinase | XP_022836433.1 | 52.32 | 3.02 ± 0.26 | 0.03 ± 0.02 |
| c72018_g1_i1 | Acyl-CoA Δ11-desaturase-like | XP_022814069.1 | 37.85 | 7.39 ± 0.22 | 0.13 ± 0.10 |
| c91688_g3_i2 | Hypothetical protein | PCG75468.1 | 33.22 | 2.97 ± 0.30 | 0.06 ± 0.03 |
| c16493_g1_i1 | Mucin-2-like | XP_013195491.1 | 32.49 | 24.10 ± 2.06 | 0.50 ± 0.07 |
| c90591_g4_i2 | Uncharacterized protein | XP_022819582.1 | 30.17 | 2.32 ± 0.26 | 0.05 ± 0.00 |
| c89395_g1_i3 | Uncharacterized protein | XP_022816309.1 | 26.59 | 0.48 ± 0.02 | 0.01 ± 0.00 |
| c78930_g1_i2 | Ribonuclease H2 subunit C | XP_022833983.1 | 20.54 | 24.32 ± 0.67 | 0.78 ± 0.15 |
| c37896_g1_i1 | Spidroin-2-like | XP_022823349.1 | 20.43 | 81.17 ± 4.50 | 2.64 ± 0.71 |
| c78055_g2_i2 | Inositol hexakisphosphate and diphosphoinositol-pentakisphosphate kinase isoform X1 | XP_013146159.1 | 20.12 | 0.71 ± 0.00 | 0.02 ± 0.01 |
| c91198_g5_i1 | Uncharacterized protein | XP_014369329.1 | 17.17 | 0.44 ± 0.05 | 0.01 ± 0.01 |
| c66727_g1_i1 | Uncharacterized protein | XP_022837608.1 | 16.22 | 2.36 ± 0.11 | 0.09 ± 0.02 |
| c84398_g1_i2 | Uncharacterized protein | XP_022833960.1 | 15.73 | 0.24 ± 0.03 | 0.01 ± 0.00 |
| c13934_g1_i1 | Uncharacterized protein | XP_022814606.1 | 14.97 | 58.16 ± 2.22 | 2.52 ± 0.08 |
| c88083_g1_i2 | Histone-lysine N-methyltransferase 2C isoform X1 | XP_022825139.1 | 14.09 | 6.00 ± 0.58 | 0.28 ± 0.07 |
| c90671_g1_i1 | Endoribonuclease Dicer-1 | XP_022832341.1 | 13.38 | 1.41 ± 0.07 | 0.07 ± 0.02 |
| c87592_g4_i1 | EH domain-binding protein 1 isoform X1 | XP_022828571.1 | 13.01 | 18.33 ± 0.52 | 0.93 ± 0.09 |
| c151374_g1_i1 | Nuclear pore membrane glycoprotein | XP_022822764.1 | 12.26 | 5.90 ± 0.41 | 0.32 ± 0.04 |
| c90598_g1_i2 | Protein diaphanous (actin-associated) | XP_021193468.1 | 11.74 | 10.57 ± 0.79 | 0.60 ± 0.08 |
| c8787_g1_i1 | LETM1 domain-containing protein 1 | XP_022832788.1 | 11.47 | 2.47 ± 0.29 | 0.14 ± 0.02 |
| c91127_g1_i1 | eIF-2-alpha kinase GCN2 | XP_022835131.1 | 11.46 | 1.96 ± 0.07 | 0.11 ± 0.03 |
| c90828_g2_i1 | Uncharacterized protein | XP_022196351.1 | 11.29 | 0.76 ± 0.07 | 0.04 ± 0.03 |
| c86411_g7_i2 | Tudor domain-containing protein 7A | XP_02119970D25 | -10.01 | 0.17 ± 0.06 | 1.06 ± 0.14 |
| c75642_g1_i1 | Pupal cuticle protein 36-like | XP_022814447.1 | -10.02 | 0.42 ± 0.08 | 2.70 ± 0.38 |
| c88315_g1_i1 | Uncharacterized protein | XP_022815068.1 | -10.13 | 0.79 ± 0.04 | 5.34 ± 0.18 |
| c77679_g1_i1 | Uncharacterized protein | XP_022817721.1 | -10.19 | 83.26 ± 0.95 | 549.33 ± 6.10 |
| c72990_g1_i2 | Uncharacterized protein | XP_022828369.1 | -10.26 | 0.36 ± 0.12 | 2.29 ± 0.45 |
| c82283_g1_i1 | Uncharacterized protein | XP_022837314.1 | -10.30 | 0.09 ± 0.04 | 0.70 ± 0.05 |
| c82655_g1_i1 | Neuropeptide cchamide-2 receptor-like | XP_022822771.1 | -10.36 | 0.36 ± 0.15 | 2.39 ± 0.32 |
| c79048_g1_i1 | Uncharacterized protein | XP_021196394.1 | -10.47 | 0.33 ± 0.04 | 2.14 ± 0.42 |
| c82543_g1_i1 | Uncharacterized protein | XP_022837740.1 | -10.52 | 0.62 ± 0.05 | 4.37 ± 0.30 |
| c80775_g1_i1 | Brachyurin-like | XP_022826846.1 | -10.53 | 0.35 ± 0.05 | 2.50 ± 0.15 |
| c79654_g1_i1 | Uncharacterized protein | XP_022814034.1 | -10.53 | 1.61 ± 0.14 | 11.24 ± 1.40 |
| c84646_g1_i1 | Integral membrane protein-like | XP_022820135.1 | -10.61 | 3.66 ± 0.16 | 25.96 ± 1.92 |
| c87444_g1_i1 | Probable uridine nucleosidase 2 isoform X1 | XP_022826762.1 | -10.62 | 16.95 ± 0.55 | 119.51 ± 7.57 |
| c84692_g2_i1 | Repat30 | AFH57150.1 | -10.63 | 38.74 ± 0.97 | 271.05 ± 7.69 |
| c81090_g1_i1 | Fatty acid-binding protein 1-like | XP_022831666.1 | -10.73 | 1.53 ± 0.16 | 10.74 ± 1.18 |
| c86568_g2_i1 | Hypothetical protein | PCG78639.1 | -10.73 | 6.24 ± 0.25 | 44.43 ± 0.86 |
| c85156_g1_i1 | Lactase-phlorizin hydrolase-like | XP_022827201.1 | -10.80 | 0.16 ± 0.03 | 1.15 ± 0.22 |
| c89281_g2_i1 | Putative inorganic phosphate cotransporter | XP_022837725.1 | -10.82 | 1.31 ± 0.04 | 9.38 ± 0.31 |
| c77371_g1_i3 | Multidrug resistance protein homolog 49-like | XP_022833220.1 | -10.84 | 1.04 ± 0.04 | 7.42 ± 0.77 |
| c83345_g2_i2 | Uncharacterized protein | XP_021184237.1 | -10.84 | 1.47 ± 0.07 | 10.61 ± 0.42 |
| c88254_g3_i1 | Uncharacterized protein | XP_022831959.1 | -10.89 | 0.20 ± 0.05 | 1.39 ± 0.38 |
| c90752_g1_i1 | Triokinase/FMN cyclase-like | XP_022814974.1 | -10.90 | 5.40 ± 0.15 | 38.91 ± 0.87 |
| c91067_g2_i1 | L-Dopachrome tautomerase yellow-f2-like | XP_022831259.1 | -10.93 | 1.61 ± 0.20 | 11.94 ± 1.17 |
| c131356_g1_i1 | Peroxidase-like | XP_022816507.1 | -10.95 | 7.66 ± 0.21 | 57.40 ± 12.05 |
| c89005_g1_i1 | Putative fatty acyl-coa reductase | XP_022824237.1 | -10.97 | 0.10 ± 0.03 | 0.72 ± 0.07 |
| c88514_g1_i2 | Facilitated trehalose transporter Tret1-like | XP_022827728.1 | -10.99 | 24.90 ± 0.33 | 179.95 ± 9.83 |
| c76571_g1_i1 | Revip | AGZ92264.1 | -11.08 | 1.53 ± 0.034 | 11.34 ± 2.43 |
| c89994_g3_i1 | Flavin-dependent monooxygenase 1 | ASV48991.1 | -11.09 | 1.86 ± 0.11 | 13.44 ± 0.78 |
| c69308_g1_i1 | Elongation of very long chain fatty acids protein | XP_022816464.1 | -11.13 | 0.63 ± 0.13 | 4.75 ± 0.17 |
| c86711_g1_i2 | Trypsin-like | XP_022818570.1 | -11.23 | 0.16 ± 0.027 | 1.17 ± 0.22 |
| c87748_g11_i1 | Signal peptidase complex subunit 3 | XP_021183812.1 | -11.28 | 0.57 ± 0.12 | 4.25 ± 0.1 |
| c90041_g2_i4 | Uncharacterized protein | XP_022825603.1 | -11.33 | 0.72 ± 0.05 | 5.40 ± 0.05 |
| c89224_g2_i1 | SET and MYND domain-containing protein 4 isoform X1 | XP_022833263.1 | -11.44 | 0.45 ± 0.04 | 3.40 ± 0.16 |
| c72861_g1_i1 | Thyroglobulin-like | XP_022830146.1 | -11.47 | 0.31 ± 0.02 | 2.34 ± 0.10 |
| c77644_g1_i1 | Uncharacterized protein | XP_021181157.1 | -11.51 | 0.05 ± 0.01 | 0.45 ± 0.14 |
| c91080_g4_i4 | Cytochrome P450 | ACL77781.1 | -11.52 | 7.74 ± 0.29 | 59.45 ± 3.60 |
| c90167_g1_i1 | Chymotrypsin-like elastase family member 2A | XP_022837673.1 | -11.54 | 1.55 ± 0.10 | 11.97 ± 0.33 |
| c82873_g1_i1 | Transcription factor SUM-1 | XP_022824203.1 | -11.58 | 0.31 ± 0.07 | 2.43 ± 0.17 |
| c70065_g1_i1 | Arginine kinase isoform X1 | XP_023950045.1 | -11.75 | 292.54 ± 6.45 | 2258.93 ± 70.34 |
| c151373_g1_i1 | Cysteine proteinase-like | XP_022831095.1 | -11.81 | 48.35 ± 0.24 | 369.94 ± 14.91 |
| c84511_g1_i1 | Integral membrane protein-like | XP_022837609.1 | -11.85 | 1.04 ± 0.06 | 8.31 ± 1.51 |
| c88441_g1_i1 | Antennal esterase CXE10 | AEJ38207.1 | -11.91 | 2.08 ± 0.12 | 16.60 ± 1.33 |
| c84570_g1_i4 | Uncharacterized protein | XP_022817417.1 | -12.00 | 26.36 ± 0.53 | 208.35 ± 9.96 |
| c89436_g4_i3 | Sarcalumenin | XP_021191092.1 | -12.20 | 7.81 ± 0.17 | 63.68 ± 2.43 |
| c89833_g1_i1 | Glucosylceramidase-like isoform X2 | XP_022832098.1 | -12.31 | 0.38 ± 0.07 | 3.09 ± 0.10 |
| c89467_g2_i1 | Ammonium transporter Rh type B | XP_022826946.1 | -12.32 | 0.92 ± 0.06 | 7.52 ± 0.28 |
| c82323_g1_i1 | Uncharacterized protein | XP_022816630.1 | -12.36 | 0.90 ± 0.15 | 7.28 ± 0.28 |
| c83826_g1_i1 | Uncharacterized protein | XP_022819827.1 | -12.36 | 0.31 ± 0.05 | 2.55 ± 0.02 |
| c87843_g4_i1 | Endocuticle structural glycoprotein sgabd-8-like | XP_022816372.1 | -12.52 | 0.26 ± 0.03 | 2.22 ± 0.14 |
| c90257_g2_i1 | 4-coumarate--coa ligase 1-like | XP_022817243.1 | -12.68 | 1.86 ± 0.08 | 15.69 ± 0.45 |
| c85371_g2_i1 | Venom carboxylesterase-6-like | XP_022822321.1 | -12.74 | 0.01 ± 0.00 | 0.14 ± 0.06 |
| c81196_g1_i2 | Formin-like protein 14 | XP_022824759.1 | -12.84 | 2.91 ± 0.05 | 24.47 ± 1.07 |
| c91091_g3_i3 | Proton-coupled folate transporter-like | XP_022817996.1 | -12.86 | 0.58 ± 0.03 | 5.01 ± 0.32 |
| c81997_g1_i1 | Protein obstructor-E-like | XP_022823095.1 | -13.11 | 6.21 ± 0.14 | 53.25 ± 2.02 |
| c81556_g1_i4 | Uncharacterized protein | XP_022828271.1 | -13.13 | 8.67 ± 0.30 | 70.60 ± 11.53 |
| c90756_g1_i1 | Uncharacterized protein | XP_022828880.1 | -13.27 | 0.55 ± 0.03 | 4.87 ± 0.03 |
| c26907_g1_i1 | Lysyl oxidase homolog 3A | XP_022827733.1 | -13.28 | 0.40 ± 0.06 | 3.53 ± 0.03 |
| c81127_g1_i3 | Gamma-aminobutyric acid receptor subunit beta-like | XP_022829486.1 | -13.36 | 0.03 ± 0.00 | 0.31 ± 0.01 |
| c82764_g2_i1 | Putative defense protein Hdd11 | XP_022820832.1 | -13.53 | 106.60 ± 1.5 | 943.88 ± 9.96 |
| c90143_g4_i4 | Tyrosine aminotransferase-like | XP_022834390.1 | -13.58 | 0.43 ± 0.03 | 3.97 ± 0.17 |
| c88796_g1_i1 | Nose resistant to fluoxetine protein 6 | XP_022829304.1 | -13.59 | 0.59 ± 0.04 | 5.34 ± 0.20 |
| c88665_g3_i2 | Glycerophosphodiester phosphodiesterase GDPD6-like | XP_022823191.1 | -13.63 | 0.09 ± 0.02 | 0.87 ± 0.08 |
| c84075_g2_i1 | Uncharacterized protein | XP_022829910.1 | -13.71 | 0.55 ± 0.04 | 4.98 ± 0.12 |
| c65796_g1_i1 | Uncharacterized protein | XP_022833361.1 | -13.72 | 1.69 ± 0.13 | 14.86 ± 2.34 |
| c89850_g1_i1 | Transferrin-like | XP_022819010.1 | -13.73 | 5.45 ± 0.21 | 49.54 ± 0.90 |
| c81794_g2_i1 | Muscle LIM protein Mlp84B isoform X2 | XP_022829413.1 | -13.83 | 14.83 ± 0.20 | 136.52 ± 5.05 |
| c135224_g1_i1 | Cuticle protein 3-like | XP_022816339.1 | -13.96 | 0.08 ± 0.02 | 0.76 ± 0.07 |
| c91307_g2_i1 | Cholinesterase 1-like | XP_022833710.1 | -14.01 | 15.89 ± 0.50 | 148.34 ± 4.44 |
| c90969_g1_i1 | Cadherin-like protein | AFH96949.1 | -14.12 | 0.14 ± 0.03 | 1.42 ± 0.23 |
| c85842_g1_i1 | UNC93-like protein | XP_022814180.1 | -14.23 | 0.75 ± 0.05 | 7.11 ± 0.24 |
| c84850_g2_i2 | Glutathione S-transferase 1-like isoform X1 | XP_022815496.1 | -14.48 | 15.76 ± 0.65 | 152.47 ± 17.38 |
| c7186_g1_i1 | Neurotrimin-like isoform X2 | XP_022828704.1 | -14.61 | 0.50 ± 0.05 | 4.81 ± 0.17 |
| c74834_g1_i1 | Hypothetical protein | PCG74580.1 | -14.63 | 10.15 ± 0.25 | 97.16 ± 4.32 |
| c87373_g1_i2 | Venom serine carboxypeptidase-like | XP_022821107.1 | -14.64 | 0.45 ± 0.10 | 4.31 ± 0.20 |
| c81459_g1_i1 | Uncharacterized protein | XP_022837318.1 | -14.67 | 66.94 ± 0.65 | 648.08 ± 18.71 |
| c62170_g1_i1 | Lens fiber major intrinsic protein-like | XP_022820243.1 | -14.67 | 14.52 ± 0.26 | 140.33 ± 2.81 |
| c80870_g1_i1 | Troponin T isoform A | AFZ93876.1 | -14.68 | 0.29 ± 0.02 | 2.74 ± 0.42 |
| c84879_g1_i4 | Organic cation transporter protein-like isoform X1 | XP_022828155.1 | -14.88 | 0.32 ± 0.02 | 3.20 ± 0.32 |
| c84887_g2_i1 | Putative nuclease HARBI1 | XP_022828419.1 | -15.07 | 0.11 ± 0.05 | 1.03 ± 0.12 |
| c74514_g1_i1 | Uncharacterized protein | XP_021181377.1 | -15.16 | 11.57 ± 0.40 | 114.44 ± 1.96 |
| c90624_g7_i1 | Hypothetical protein | CCQ19275.1 | -15.34 | 200.73 ± 0.20 | 1990.69 ± 126.04 |
| c131378_g1_i1 | Uncharacterized protein | XP_022832977.1 | -15.35 | 0.01 ± 0.00 | 0.16 ± 0.02 |
| c84037_g2_i1 | General binding protein 1 | ACY78412.1 | -15.36 | 0.46 ± 0.08 | 4.67 ± 0.26 |
| c80956_g1_i1 | Uncharacterized protein | XP_022814955.1 | -15.39 | 2.77 ± 0.15 | 27.56 ± 0.53 |
| c86648_g5_i1 | Hypothetical protein | PCG75468.1 | -15.40 | 0.05 ± 0.00 | 0.57 ± 0.08 |
| c91301_g1_i3 | Protein anon-37Cs-like isoform X4 | XP_022833972.1 | -15.57 | 9.84 ± 0.15 | 100.68 ± 3.26 |
| c86898_g1_i2 | Uncharacterized protein | XP_022825603.1 | -15.57 | 6.06 ± 0.17 | 62.17 ± 3.76 |
| c79668_g1_i1 | Uncharacterized protein | XP_022837660.1 | -15.63 | 0.29 ± 0.09 | 2.84 ± 0.33 |
| c89526_g1_i1 | Glucose dehydrogenase | XP_022830845.1 | -15.74 | 0.83 ± 0.00 | 8.74 ± 0.07 |
| c87214_g2_i1 | Putative inorganic phosphate cotransporter | XP_022837716.1 | -15.92 | 1.00 ± 0.06 | 10.46 ± 1.21 |
| c85905_g4_i1 | Mitochondrial enolase superfamily member 1-like | XP_022821602.1 | -16.02 | 19.07 ± 0.65 | 200.84 ± 7.43 |
| c84809_g2_i1 | Tubulin beta chain-like | XP_022833858.1 | -16.09 | 0.58 ± 0.10 | 5.97 ± 0.59 |
| c53937_g1_i1 | Flightin isoform X2 | XP_022832756.1 | -16.21 | 0.55 ± 0.07 | 5.73 ± 0.11 |
| c82867_g1_i2 | Integral membrane protein-like | XP_022819939.1 | -16.23 | 0.15 ± 0.01 | 1.61 ± 0.21 |
| c80974_g1_i1 | Uncharacterized protein | XP_022828202.1 | -16.31 | 141.96 ± 12.35 | 1495.76 ± 76.83 |
| c86439_g2_i1 | Decaprenyl-diphosphate synthase subunit 2 | XP_022816493.1 | -16.33 | 0.10 ± 0.01 | 1.15 ± 0.16 |
| c85304_g1_i2 | Aquaporin-11 isoform X2 | XP_022822820.1 | -16.33 | 0.75 ± 0.05 | 8.28 ± 0.18 |
| c154405_g1_i1 | Circadian clock-controlled protein-like | XP_021182291.1 | -16.39 | 0.16 ± 0.03 | 1.82 ± 0.31 |
| c79450_g1_i1 | Tropomyosin-1 | KOB78933.1 | -16.67 | 0.15 ± 0.05 | 1.69 ± 0.03 |
| c76801_g1_i1 | Endocuticle structural glycoprotein ABD-5-like | XP_022817375.1 | -16.68 | 0.44 ± 0.08 | 4.59 ± 0.53 |
| c90334_g4_i1 | Sodium-coupled monocarboxylate transporter 2-like | XP_022829913.1 | -16.73 | 0.27 ± 0.05 | 3.06 ± 0.14 |
| c86908_g1_i1 | Four and a half LIM domains protein 2 isoform X5 | XP_021187969.1 | -16.91 | 43.48 ± 1.30 | 480.44 ± 48.71 |
| c37951_g1_i1 | Takeout | ATU07277.1 | -17.52 | 0.05 ± 0.01 | 0.64 ± 0.10 |
| c91951_g2_i1 | Ryanodine receptor | ALL55469.1 | -17.62 | 0.43 ± 0.01 | 5.07 ± 0.26 |
| c86523_g5_i1 | Glycine-rich protein DOT1-like | XP_022837431.1 | -17.64 | 1.75 ± 0.13 | 20.23 ± 1.41 |
| c88924_g2_i2 | Uncharacterized protein | XP_022829606.1 | -17.86 | 0.78 ± 0.01 | 9.49 ± 1.70 |
| c83086_g1_i1 | Aminopeptidase N | AAT99437.1 | -17.97 | 0.04 ± 0.00 | 0.52 ± 0.09 |
| c83704_g1_i1 | Uncharacterized protein | XP_022826807.1 | -18.23 | 0.31 ± 0.02 | 3.66 ± 0.15 |
| c77762_g1_i2 | Dentin sialophosphoprotein-like | XP_022829288.1 | -18.45 | 2.96 ± 0.07 | 34.52 ± 6.30 |
| c80458_g1_i1 | Uncharacterized protein | XP_022817958.1 | -18.74 | 0.07 ± 0.02 | 0.91 ± 0.15 |
| c90139_g1_i1 | Prostacyclin receptor | XP_022815197.1 | -19.08 | 0.19 ± 0.02 | 2.44 ± 0.24 |
| c86319_g1_i1 | Clavesin-2-like | XP_022832976.1 | -19.30 | 0.86 ± 0.04 | 11.18 ± 0.60 |
| c84878_g1_i2 | Odorant binding protein 19 | AKT26497.1 | -19.39 | 0.64 ± 0.06 | 8.32 ± 0.94 |
| c89867_g2_i1 | UDP-glycosyltransferase 40D3 | ANI22000.1 | -19.43 | 3.96 ± 0.20 | 51.12 ± 2.40 |
| c78043_g1_i1 | Paramyosin, long form-like | XP_022832505.1 | -19.71 | 30.84 ± 0.30 | 398.95 ± 30.27 |
| c87203_g1_i1 | Uncharacterized protein | XP_022834103.1 | -19.81 | 0.18 ± 0.015 | 2.46 ± 0.30 |
| c114645_g1_i1 | Organic cation transporter protein-like | XP_022828278.1 | -19.82 | 0.06 ± 0.01 | 0.83 ± 0.06 |
| c90231_g1_i1 | Putative GPI-anchored protein pfl2 | XP_022821793.1 | -19.87 | 4.07 ± 0.06 | 53.73 ± 0.55 |
| c83863_g1_i1 | Uncharacterized protein | XP_022834158.1 | -19.99 | 2.03 ± 0.60 | 24.74 ± 3.22 |
| c82221_g1_i1 | Solute carrier family 22 member 13-like | XP_021194638.1 | -20.29 | 0.10 ± 0.04 | 1.38 ± 0.33 |
| c87705_g1_i1 | Cuticle protein 16.5 | XP_022830400.1 | -20.61 | 1.73 ± 0.13 | 23.05 ± 1.59 |
| c88610_g1_i1 | Uncharacterized protein | XP_022830389.1 | -20.65 | 4.77 ± 0.20 | 66.02 ± 6.00 |
| c79518_g1_i1 | Endocuticle structural glycoprotein ABD-4-like | XP_022819178.1 | -20.76 | 0.29 ± 0.30 | 3.84 ± 0.42 |
| c89813_g1_i1 | Phosphoenolpyruvate carboxykinase [GTP]-like isoform X2 | XP_022818284.1 | -20.90 | 14.96 ± 0.20 | 205.12 ± 16.06 |
| c91253_g2_i5 | Filamin-A isoform X1 | XP_022834668.1 | -20.90 | 0.68 ± 0.07 | 9.42 ± 0.80 |
| c91318_g1_i1 | Putative aminopeptidase W07G4.4 | XP_022831659.1 | -21.05 | 7.45 ± 0.10 | 104.33 ± 1.91 |
| c86614_g2_i1 | Phytanoyl-coa dioxygenase domain-containing protein 1-like | XP_022832954.1 | -21.34 | 0.04 ± 0.00 | 0.50 ± 0.01 |
| c90753_g1_i1 | Uncharacterized protein | XP_022815519.1 | -21.36 | 0.07 ± 0.01 | 1.17 ± 0.31 |
| c90872_g1_i1 | Retroelement polyprotein | ACE75273.1 | -21.97 | 0.06 ± 0.00 | 0.97 ± 0.02 |
| c86646_g1_i1 | Trypsin, alkaline B-like | XP_022836358.1 | -21.98 | 0.05 ± 0.02 | 0.80 ± 0.09 |
| c62474_g1_i1 | Hypothetical protein | PCG66042.1 | -22.15 | 0.65 ± 0.09 | 9.81 ± 2.95 |
| c87507_g1_i1 | Facilitated trehalose transporter Tret1-2 homolog | XP_022826132.1 | -22.16 | 0.14 ± 0.05 | 2.07 ± 0.05 |
| c88814_g1_i1 | Uncharacterized protein | XP_022816392.1 | -22.50 | 0.22 ± 0.07 | 3.31 ± 0.28 |
| c84868_g1_i1 | Uncharacterized protein | XP_022829627.1 | -22.60 | 138.59 ± 0.28 | 2074.64 ± 65.05 |
| c83093_g2_i1 | Carboxypeptidase B-like | XP_022818071.1 | -22.66 | 0.04 ± 0.02 | 0.66 ± 0.10 |
| c73755_g1_i1 | Paxillin isoform X4 | XP_022819965.1 | -22.83 | 11.81 ± 0.30 | 177.87 ± 5.12 |
| c81523_g1_i1 | Uncharacterized protein | XP_022824823.1 | -23.05 | 0.04 ± 0.00 | 0.64 ± 0.08 |
| c20471_g1_i1 | Uncharacterized protein | KOB70863.1 | -23.13 | 1.10 ± 0.10 | 16.51 ± 1.03 |
| c87388_g2_i4 | Hypothetical protein | PCG75564.1 | -23.19 | 0.05 ± 0.01 | 0.76 ± 0.04 |
| c85113_g1_i1 | Cytochrome P450 4C1-like isoform X1 | XP_022827530.1 | -23.20 | 0.19 ± 0.03 | 2.99 ± 0.21 |
| c84485_g1_i1 | Uncharacterized protein | XP_022831758.1 | -23.39 | 0.41 ± 0.04 | 6.53 ± 0.07 |
| c85122_g5_i1 | Inducible metalloproteinase inhibitor protein-like | XP_022816597.1 | -23.43 | 3.96 ± 0.40 | 62.23 ± 10.68 |
| c66492_g1_i1 | Uncharacterized protein | XP_022825557.1 | -24.03 | 1.87 ± 0.04 | 29.87 ± 2.80 |
| c90134_g1_i1 | Alpha-N-acetylgalactosaminidase isoform X2 | XP_022823580.1 | -24.38 | 3.09 ± 0.05 | 49.98 ± 0.31 |
| c57946_g1_i1 | Intestinal mucin, partial | ABW06596.1 | -24.52 | 0.19 ± 0.04 | 3.18 ± 0.08 |
| c87445_g1_i1 | Myosin heavy chain, muscle isoform X1 | XP_021194457.1 | -24.58 | 38.26 ± 0.6 | 618.89 ± 48.32 |
| c81906_g1_i1 | Takeout | ATU07284.1 | -25.53 | 1.63 ± 0.13 | 27.20 ± 1.13 |
| c87993_g1_i2 | Cytochrome p450 CYP367B1 | ASO98048.1 | -26.54 | 0.15 ± 0.02 | 2.84 ± 0.32 |
| c75827_g1_i1 | Uncharacterized protein | XP_022817954.1 | -26.73 | 1.40 ± 0.09 | 24.38 ± 1.42 |
| c85592_g1_i1 | Uncharacterized protein | XP_022822432.1 | -26.75 | 0.14 ± 0.01 | 2.51 ± 0.04 |
| c88703_g1_i1 | Protein lethal(3)malignant blood neoplasm 1 | XP_022816605.1 | -26.97 | 0.23 ± 0.01 | 4.24 ± 0.13 |
| c79092_g1_i3 | Diapausin precursor | ABU96713.1 | -27.05 | 333.53 ± 6.1 | 5634.98 ± 435.61 |
| c73849_g1_i1 | Uncharacterized protein | XP_022822390.1 | -27.67 | 2.11 ± 0.05 | 38.52 ± 1.02 |
| c84343_g1_i1 | Protein lethal(2)essential for life-like | XP_021194612.1 | -28.02 | 0.56 ± 0.03 | 10.55 ± 1.16 |
| c12887_g1_i1 | Fatty acid-binding protein 1-like | XP_022831716.1 | -28.34 | 0.11 ± 0.02 | 2.20 ± 0.27 |
| c84157_g1_i1 | Uncharacterized protein | XP_022830099.1 | -28.53 | 0.04 ± 0.01 | 0.78 ± 0.14 |
| c86248_g1_i1 | Midgut class 1 aminopeptidase N | AAP44964.1 | -29.01 | 0.08 ± 0.00 | 1.68 ± 0.37 |
| c91462_g1_i1 | Very high density lipoprotein | ABQ23674.1 | -29.27 | 63.19 ± 0.24 | 1218.62 ± 70.32 |
| c89307_g1_i1 | Serine protease 7-like | XP_022837507.1 | -29.79 | 0.52 ± 0.01 | 10.33 ± 0.60 |
| c77196_g1_i1 | Venom peptide bmkapi-like | XP_022826199.1 | -29.81 | 0.71 ± 0.35 | 14.51 ± 1.80 |
| c89636_g1_i1 | Uncharacterized protein | XP_022824038.1 | -30.46 | 1.41 ± 0.08 | 28.46 ± 1.26 |
| c85605_g1_i1 | Uncharacterized protein | XP_022816237.1 | -30.93 | 1.53 ± 0.16 | 30.87 ± 2.35 |
| c86935_g2_i1 | Uncharacterized protein | XP_022827220.1 | -31.18 | 0.23 ± 0.01 | 4.79 ± 0.25 |
| c80561_g1_i1 | Uncharacterized protein | XP_022824813.1 | -31.18 | 0.46 ± 0.02 | 9.61 ± 0.24 |
| c83464_g1_i1 | Uncharacterized protein | XP_022824824.1 | -31.19 | 0.05 ± 0.01 | 1.14 ± 0.04 |
| c84894_g1_i1 | Circadian clock-controlled protein-like | XP_022828097.1 | -31.99 | 12.70 ± 0.50 | 271.94 ± 29.94 |
| c87907_g2_i1 | Uncharacterized protein | XP_022819923.1 | -32.20 | 0.07 ± 0.01 | 1.75 ± 0.51 |
| c65560_g1_i1 | Tyrosine hydroxylase | AFG25778.1 | -32.67 | 2.58 ± 0.06 | 55.82 ± 1.37 |
| c76852_g1_i1 | TSC22 domain family protein 1-like | XP_022828041.1 | -32.83 | 0.35 ± 0.1 | 7.54 ± 0.05 |
| c87118_g1_i1 | Uncharacterized protein | XP_022817797.1 | -32.97 | 0.53 ± 0.05 | 11.73 ± 0.18 |
| c84667_g1_i1 | Odorant binding protein 25 | AKT26502.1 | -33.57 | 12.93 ± 0.06 | 284.66 ± 6.28 |
| c79263_g2_i3 | Sodium/potassium/calcium exchanger 4-like | XP_022832744.1 | -33.79 | 0.03 ± 0.00 | 0.68 ± 0.07 |
| c56513_g1_i1 | Hypothetical protein | PCG78433.1 | -33.98 | 20.41 ± 0.50 | 440.68 ± 15.98 |
| c85017_g2_i1 | Cuticle protein 7-like isoform X1 | XP_022816468.1 | -34.68 | 0.12 ± 0.02 | 2.61 ± 0.36 |
| c82225_g1_i1 | Odorant binding protein | ADY17884.1 | -34.70 | 26.09 ± 0.21 | 599.66 ± 52.90 |
| c86064_g1_i1 | Estrogen sulfotransferase-like | XP_022819760.1 | -34.96 | 0.96 ± 0.08 | 23.02 ± 6.10 |
| c88235_g1_i1 | Uncharacterized protein | XP_022837495.1 | -35.21 | 0.26 ± 0.04 | 6.32 ± 0.10 |
| c88984_g2_i1 | Delta-9 desaturase 14-26 | AFO38465.1 | -35.91 | 0.52 ± 0.03 | 12.44 ± 0.86 |
| c81489_g1_i1 | Nuclear pore complex protein DDB_G0274915-like | XP_022820193.1 | -35.95 | 0.50 ± 0.08 | 11.86 ± 0.63 |
| c76782_g3_i1 | Myosin light chain alkali isoform X1 | XP_022826084.1 | -36.09 | 48.40 ± 1.00 | 1150.85 ± 44.69 |
| c88107_g1_i1 | Carbohydrate sulfotransferase 4-like | XP_022829420.1 | -36.14 | 0.03 ± 0.01 | 0.74 ± 0.00 |
| c80037_g1_i1 | Cytochrome b5-like | XP_022823555.1 | -36.40 | 0.07 ± 0.02 | 1.74 ± 0.34 |
| c85234_g1_i1 | Uncharacterized protein | XP_022828370.1 | -39.45 | 0.95 ± 0.06 | 25.67 ± 6.32 |
| c89996_g1_i1 | Synaptic vesicle glycoprotein 2B-like | XP_022826968.1 | -40.65 | 0.75 ± 0.03 | 20.23 ± 0.65 |
| c89066_g1_i1 | Hemolin-like | XP_022819117.1 | -40.93 | 156.15 ± 2.00 | 4234.66 ± 161.84 |
| c86705_g1_i1 | Mediator of RNA polymerase II transcription subunit 15-like | XP_022828744.1 | -42.26 | 0.27 ± 0.05 | 7.65 ± 0.35 |
| c75587_g1_i1 | TSC22 domain family protein 1-like | XP_022828041.1 | -43.01 | 0.65 ± 0.01 | 18.44 ± 0.63 |
| c83058_g1_i2 | Uncharacterized protein | XP_022815538.1 | -43.42 | 1.17 ± 0.10 | 33.50 ± 0.25 |
| c65485_g1_i1 | Actin, muscle-type A2 | NP_001119725.1 | -44.34 | 1.16 ± 0.30 | 32.21 ± 2.51 |
| c85594_g1_i1 | Insecticyanin-B-like | XP_022817843.1 | -44.38 | 6.31 ± 0.30 | 183.62 ± 1.81 |
| c89285_g1_i1 | Lachesin isoform X1 | XP_022832190.1 | -44.66 | 0.13 ± 0.02 | 3.79 ± 0.09 |
| c86430_g1_i1 | Counting factor associated protein D-like | XP_022824876.1 | -45.05 | 1.15 ± 0.10 | 33.78 ± 3.67 |
| c86772_g1_i1 | Monocarboxylate transporter 2-like | XP_022829653.1 | -45.95 | 0.11 ± 0.02 | 3.49 ± 0.77 |
| c85248_g1_i1 | Serine protease snake-like | XP_022824733.1 | -46.85 | 0.21 ± 0.02 | 6.56 ± 0.03 |
| c89952_g3_i1 | Mucin-2-like | XP_022823928.1 | -47.88 | 0.68 ± 0.08 | 21.03 ± 1.17 |
| c45151_g1_i1 | Uncharacterized protein | XP_022824375.1 | -53.41 | 0.31 ± 0.08 | 11.50 ± 2.65 |
| c90196_g2_i1 | Luciferin 4-monooxygenase-like | XP_022831803.1 | -54.32 | 0.23 ± 0.01 | 8.62 ± 0.39 |
| c76799_g1_i1 | Collagen alpha-1(IV) chain-like | XP_022828198.1 | -56.37 | 3.14 ± 0.40 | 114.87 ± 7.01 |
| c80423_g1_i1 | Uncharacterized protein | XP_021195335.1 | -56.68 | 0.88 ± 0.17 | 32.09 ± 2.14 |
| c89006_g2_i3 | Luciferin 4-monooxygenase-like | XP_022831950.1 | -57.64 | 0.90 ± 0.10 | 35.33 ± 6.77 |
| c81196_g2_i1 | Proline-rich extensin-like protein EPR1 | XP_022816193.1 | -57.74 | 1.07 ± 0.10 | 41.03 ± 1.08 |
| c52918_g1_i1 | Uncharacterized protein | XP_022826722.1 | -58.25 | 0.13 ± 0.05 | 5.60 ± 0.16 |
| c84177_g1_i1 | PREDICTED: mucin-2-like | XP_013195491.1 | -58.35 | 0.08 ± 0.02 | 3.36 ± 0.69 |
| c90872_g2_i3 | Retroelement polyprotein | OWR54321.1 | -59.37 | 0.09 ± 0.02 | 3.62 ± 0.11 |
| c88378_g1_i2 | Luciferin 4-monooxygenase-like | XP_022831919.1 | -60.41 | 0.13 ± 0.02 | 5.12 ± 0.29 |
| c88384_g1_i1 | Alpha-tocopherol transfer protein-like | XP_022832175.1 | -61.69 | 0.30 ± 0.04 | 12.47 ± 0.73 |
| c73815_g2_i1 | Glycine-rich protein DOT1-like | XP_022819775.1 | -61.93 | 0.15 ± 0.02 | 6.20 ± 0.39 |
| c83863_g2_i1 | Repat35 | AFH57155.1 | -67.69 | 0.41 ± 0.05 | 18.03 ± 2.85 |
| c88270_g9_i1 | Trypsin, alkaline C-like | XP_022815733.1 | -70.00 | 0.10 ± 0.02 | 4.96 ± 1.27 |
| c12902_g1_i1 | 4-hydroxyphenylpyruvate dioxygenase-like | XP_022837711.1 | -71.15 | 2.74 ± 0.12 | 127.24 ± 5.40 |
| c77112_g1_i1 | LOW QUALITY PROTEIN: larval cuticle protein 1-like | XP_022816717.1 | -86.40 | 169.74 ± 2.30 | 9530.93 ± 176.65 |
| c86472_g1_i2 | Uncharacterized protein | XP_022834551.1 | -92.41 | 0.26 ± 0.03 | 16.47 ± 1.85 |
| c81880_g1_i2 | Uncharacterized protein | XP_022816303.1 | -98.71 | 0.07 ± 0.02 | 5.07 ± 0.19 |
| c113540_g1_i1 | Intestinal mucin, partial | ABW06596.1 | -101.41 | 0.10 ± 0.02 | 6.66 ± 0.68 |
| c85883_g1_i1 | Facilitated trehalose transporter Tret1-like | XP_022835083.1 | -107.25 | 0.01 ± 0.00 | 1.07 ± 0.06 |
| c82096_g1_i1 | Modular serine protease-like, partial | XP_022834261.1 | -109.47 | 0.40 ± 0.03 | 30.25 ± 7.00 |
| c76641_g1_i1 | Repat33 | AFH57153.1 | -110.72 | 0.10 ± 0.02 | 7.69 ± 0.91 |
| c69668_g1_i1 | Collagen alpha-1(IV) chain-like | XP_022828198.1 | -120.66 | 7.59 ± 0.30 | 564.42 ± 27.83 |
| c56658_g1_i1 | Phytanoyl-coa dioxygenase, peroxisomal-like | XP_022826869.1 | -122.19 | 3.13 ± 0.20 | 252.51 ± 16.92 |
| c151202_g1_i1 | Hypothetical protein | PCG75674.1 | -125.07 | 0.33 ± 0.10 | 27.31 ± 0.10 |
| c87369_g3_i1 | Calphotin-like | XP_022813970.1 | -127.03 | 0.32 ± 0.06 | 26.89 ± 5.89 |
| c69780_g1_i1 | Hypothetical protein | PCG78433.1 | -128.90 | 0.66 ± 0.20 | 53.81 ± 5.17 |
| c79609_g1_i1 | Chemosensory protein 19 | AKT26493.1 | -141.29 | 0.46 ± 0.06 | 42.85 ± 3.58 |
| c85946_g2_i1 | Chemosensory protein 13 | AKT26489.1 | -166.89 | 1.71 ± 0.13 | 186.86 ± 3.97 |
| c81366_g1_i2 | Uncharacterized protein | XP_022834890.1 | -175.06 | 0.14 ± 0.01 | 16.11 ± 2.26 |
| c85535_g1_i1 | Lipase 1-like | XP_022831142.1 | -212.08 | 0.16 ± 0.00 | 23.22 ± 5.13 |
| c112321_g1_i1 | Acyl-coa-binding protein-like | XP_022820273.1 | -225.94 | 0.39 ± 0.03 | 57.34 ± 9.92 |
| c81979_g1_i1 | Regucalcin-like | XP_022815254.1 | -233.67 | 1.30 ± 0.08 | 198.07 ± 13.43 |
| c83233_g1_i1 | Endocuticle structural glycoprotein sgabd-5-like isoform X1 | XP_022816395.1 | -247.46 | 1.60 ± 0.17 | 258.94 ± 2.10 |
| c81169_g1_i1 | Circadian clock-controlled protein-like | XP_022828332.1 | -311.88 | 0.75 ± 0.06 | 158.10 ± 17.56 |
| c67156_g1_i1 | Hypothetical protein | PCG62835.1 | -315.48 | 0.09 ± 0.00 | 19.43 ± 3.68 |
| c87369_g2_i1 | Calphotin-like | XP_022817201.1 | -402.42 | 0.48 ± 0.14 | 135.13 ± 27.86 |
| c112396_g1_i1 | Flexible cuticle protein 12-like | XP_022825251.1 | -509.57 | 0.58 ± 0.20 | 183.68 ± 12.39 |
| c82011_g1_i1 | Trypsin, alkaline C-like | XP_022815740.1 | -669.78 | 0.18 ± 0.04 | 82.19 ± 16.95 |
| c86481_g2_i1 | Serpin-Z4-like | XP_022837525.1 | -695.09 | 0.14 ± 0.04 | 68.64 ± 2.01 |
| c84846_g1_i1 | Uncharacterized protein | XP_022822932.1 | -722.34 | 0.08 ± 0.02 | 43.81 ± 8.58 |
| c85150_g1_i1 | Larval cuticle protein 16/17-like | XP_022816379.1 | -973.92 | 6.37 ± 0.46 | 3971.57 ± 208.70 |
| c82495_g1_i1 | Larval cuticle protein LCP-30-like | XP_021201007.1 | -1100.97 | 0.25 ± 0.05 | 187.91 ± 6.93 |
| c73601_g2_i1 | Uncharacterized protein | KOB74345.1 | -1500.72 | 0.27 ± 0.07 | 257.65 ± 20.21 |
