# Supplementary material for: A PLA2 deletion mutant using CRISPR/Cas9 coupled to RNASeq reveals insect immune genes associated with eicosanoid signaling
Source: PLoS One. 2024 Jul 17;19(7):e0304958. doi: 10.1371/journal.pone.0304958 (PMC11253937; doi:10.1371/journal.pone.0304958)
Supplement: S1 Raw images — (PDF) [file pone.0304958.s008.pdf]

Fig 1A Relative sPLA2 expression

	Egg	L1	L2	L3	L4	L5	Pupa	Male	Female
R1	1.029373	5.6383	8.783629	20.12086	57.58079	44.47749	1.546695	7.834616	10.39034
R2	1.031251	5.158252	7.850467	19.8529	72.53469	62.74265	1.100114	5.721395	9.888737
R3	0.939376	4.713091	5.696074	21.26075	73.12367	64.87689	1.289261	7.650568	15.52017

Fig 1B Specific enzyme activity

	Egg	L1	L2	L3	L4	L5	Pupa	Male	Female
R1	0.00038	0.00163	0.002237	0.002208	0.007759	0.005686	0.001451	0.001733	0.001375
R2	0.000544	0.002018	0.002553	0.002225	0.007922	0.005576	0.001214	0.002384	0.002404
R3	0.00095	0.001818	0.001972	0.001996	0.007706	0.005991	0.001235	0.001487	0.001009

Fig 1C Specific enzyme activity

	Naïve	Chalenge
R1	0.001644	0.005094
R2	0.001674	0.005198
R3	0.001549	0.005519

Fig 4D

		Naïve	Immune	DsPLA2	Immune+dsPLA2	
Reco	PGRP-S1	1	5.359223	0.407767	1.563107	PRP
	PGRP-S5	1	3.244066	0.392392	0	
	PGRP-S2	1	2.239623	0.041509	1.216981	
	galectin-5-l	1	3.089905	0.370137	0	
	C-type lecti	1	3.408163	0	0	
	C-type lecti	1	6.6	0.066667	0.226667	
	C-type lecti	1	2.96	0.333333	0.186667	
	BmCTL10	1	7.837838	3.094595	0.432432	
	C-type lecti	1	10.5	0.216667	2.833333	
	lectin 5	1	3.454545	0.227273	0.318182	
	50kDa lecti	1	2.25	2.5	0	
	scavenger	1	1.961538	0.423077	1.038462	
	nimrod B	1	3.159841	0	0	
Medi	spatzle-like	1	0.191781	0.064384	0	Toll
	toll-like rec	1	0.035211	0.042254	0	
	protein toll-	1	9.102564	3.512821	5.948718	
	protein toll-	1	7.272727	3.506494	4.545455	
	toll-like rec	1	2.333333	0.333333	0	
	toll-like rec	1	2	2.714286	0	
	Pelle	1	2.333333	0	0	
	Cactus	1	2.248062	0	0	
	Relish	1	5.664336	0.615385	0	IMD
	Fadd	1	2.180602	0.501672	0	
	ERK	1	2.316265	12.1988	8.945783	
	Hopscotch	1	1.906977	0	0	JAK/STAT
	JAK	1	2.746715	0	0	
	STAT	1	3.098976	14.23208	21.22526	

	NOS	1	10.0443	0	0	NOS
Effec	Apolipopho	1	4.581543	0.459944	0	AMP
	Attacin	1	4.407984	0.183539	2.161646	
	cecropin-D·	1	11.74992	0	0	
	cecropin-B	1	3.854732	0.095529	0.094843	
	lysozyme	1	2.927928	0.105105	1.6997	
	lysozyme-li	1	4.02963	0	0	
	lysozyme 2	1	1.96875	0.40625	0.675	
	lysozyme 1	1	9.181124	0.191505	2.382142	
	moricin-1-li	1	16.65252	0.483491	2.044811	
	Moricin	1	14.58333	6.333333	12.625	
	Transferrin	1	5.554305	0	0	
	Gallerimyci	1	8.902225	0	0	
	Cecropin-C	1	11.74992	0	0	
	Gloverin	1	12.19565	0	0	
	Hemolin	1	60.25381	0.067032	0.350904	
	Serpin 1	1	6.409179	0	0	PO
	Serpin 3	1	2.704078	0	0	
	Serpin 10	1	0.161929	0.01685	0.067572	
	Serpin 27	1	0.347997	0.321875	0	
	Serpin 9	1	2.051627	0.364433	0.689409	
	Serpin 6	1	2.442611	0	0	
	Serpin 32	1	2.011646	0	0	
	Serpin B4-l	1	2.475463	0	0	
	Serpin B5-l	1	3.952392	1	0.008447	
	Serpin B8-l	1	2.977134	0.528746	0	
	Antichymot	1	10.24574	0	0	
	modular se	1	4.510756	0.478834	0	
	PO	1	0.638091	0.461818	0.488095	
	PAP	1	2.175328	0.716994	1.147238	
	Dual oxidas	1	2.744681	0	0	ROS
	NADPH oxi	1	1.842105	0	0	
	Caspase	1	2.078927	0	0	Apoptosis
	TNF recept	1	0.193333	0.326667	0	

Fig 5A Nodule pe

plicati	ddH2o	WT	ΔsPLA2	ΔsPLA2+AA
1	2	65	10	60
2	3	73	11	28
3	2	75	27	22
4	4	51	12	34
5	5	62	25	32
6	2	68	42	39
7	0	83	38	65
8	2	88	11	68
9	4	61	32	71
10	4	57	8	75

Fig 5B

' expression-Fat b

	Treatments	R1	R2	R3
	WT-Naïve	0.405653	0.611095	0.408199
Apo	WT-Immun	1.814547	1.58353	1.323445
	ΔsPLA2-Imı	0.009882	0.008945	0.012158
	WT-Naïve	0.2707	0.259016	0.203967
Att1	WT-Immun	1.803874	1.968843	1.635593
	ΔsPLA2-Imı	0.147399	0.138321	0.288956
	WT-Naïve	0.337676	0.415756	0.513942
Att 2	WT-Immun	1.674673	1.326117	1.512612
	ΔsPLA2-Imı	0.369954	0.230887	0.158941
	WT-Naïve	0.358231	0.417927	0.308066
Def	WT-Immun	1.859641	1.271949	1.193074
	ΔsPLA2-Imı	0.010292	0.006599	0.009703
	WT-Naïve	0.479655	0.361417	0.450045
Gal	WT-Immun	0.955691	1.086218	0.713753
	ΔsPLA2-Imı	0.033516	0.015622	0.045631
	WT-Naïve	0.414581	0.506902	0.32052
Tf1	WT-Immun	1.303256	1.452833	1.205027
	ΔsPLA2-Imı	0.012284	0.011445	0.017515
	WT-Naïve	0.610852	0.719316	0.550664
Tf2	WT-Immun	1.58894	2.145235	1.353088
	ΔsPLA2-Imı	0.567308	0.525196	0.463955
	WT-Naïve	0.491725	0.418486	0.317697
Hem	WT-Immun	1.890011	1.636651	2.060342
	ΔsPLA2-Imı	0.487028	0.30224	0.434776

expression-Hem

	Treatments	R1	R2	R3
	WT-Naïve	0.483647	0.401179	0.385789
Apo	WT-Immun	1.276387	0.9074	1.469175
	ΔsPLA2-Imı	0.052941	0.057908	0.057884

	WT-Naïve	0.202386	0.254721	0.226058
Att1	WT-Immun	1.009255	0.720531	0.85039
	ΔsPLA2-Imı	0.049378	0.06354	0.058119
	WT-Naïve	0.450852	0.359316	0.350664
Att 2	WT-Immun	1.153129	1.312535	0.949821
	ΔsPLA2-Imı	0.281316	0.27392	0.294699
	WT-Naïve	0.302743	0.281937	0.240143
Def	WT-Immun	0.954399	1.650346	1.274514
	ΔsPLA2-Imı	0.028527	0.029667	0.023006
	WT-Naïve	0.284581	0.196902	0.31052
Gal	WT-Immun	0.573122	0.669015	0.458172
	ΔsPLA2-Imı	0.065186	0.07895	0.050239
	WT-Naïve	0.237773	0.17763	0.203156
Tf1	WT-Immun	0.986675	1.080759	0.762566
	ΔsPLA2-Imı	0.051606	0.057407	0.058824
	WT-Naïve	0.342469	0.314049	0.284275
Tf2	WT-Immun	0.972645	1.552886	1.083778
	ΔsPLA2-Imı	0.004223	0.004514	0.004232
	WT-Naïve	0.225298	0.216072	0.213727
Hem	WT-Immun	1.454084	1.170199	1.675654
	ΔsPLA2-Imı	0.19414	0.243922	0.220165

Fig 6B

Pupal weight					
ΔsPLA2		WT			
eatment No.	Weight(mg)	CON No.	Weight (mg)		
2	74		Replication #	eplication #	eplication #3
91	37	1	123	105	119
67	48	2	119	107	111
54	90	3	122	109	128
59	88	4	122	108	125
48	79	5	103	125	101
77	30	6	105	88	85
94	59	7	96	73	90
52	44	8	122	75	92
53	58	9	108	107	84
13	57	10	114	125	117

56	70
1	33
49	68
107	53
140	61
81	37
74	49
98	86
95	46
114	39
93	45
139	57
66	55
50	76
65	87

pupation rate

	percent of pupated insect in WT		
	Replication #eplication #eplication #3		
r of p	26/30	24/30	30/30

	Jumber of pupated insect in ΔsPLA		
	Replication #eplication #eplication #3		
r of p	10 of 30	9 of 30	9 of 30

Fig 6C

	Number of laid eggs/female			
	WT (virigin)	sPLA2 (virigi	WT (mated)	PLA2 (mated)
1	75	59	752	495
2	62	41	655	415
3	58	22	221	225
4	90	35	565	45
5	51	24	480	39
6	85	13	520	85
7	37	4	212	73
8	88	22	480	382
9	102	22	158	59
10	48	8	620	34

Egg hatching

Jumber of eggs were hatched in W		
Replication #eplication #eplication #3		
377 of 400	400 of 400	400 of 400

mber of eggs were hatched in ΔsPL		
Replication #	eplication #	eplication #3
48 of 200	59 of 200	65 of 200
